# Exploring Experimental Models of Colorectal Cancer: A Critical Appraisal from 2D Cell Systems to Organoids, Humanized Mouse Avatars, Organ-on-Chip, CRISPR Engineering, and AI-Driven Platforms—Challenges and Opportunities for Translational Precision Oncology

**DOI:** 10.3390/cancers17132163

**Published:** 2025-06-26

**Authors:** Ahad Al-Kabani, Bintul Huda, Jewel Haddad, Maryam Yousuf, Farida Bhurka, Faika Ajaz, Rajashree Patnaik, Shirin Jannati, Yajnavalka Banerjee

**Affiliations:** Department of Basic Medical Sciences, College of Medicine and Health Sciences, Mohammed Bin Rashid University of Medicine and Health Sciences (MBRU), Dubai Health, AMC Building 14, Dubai 505055, United Arab Emiratesfarida.bhurka@students.mbru.ac.ae (F.B.);

**Keywords:** colorectal cancer models, in vivo CRC models, humanized mouse models, tumor-on-a-chip, artificial intelligence, patient-derived xenografts, CRC translational research, organoid models, preclinical CRC modeling, experimental oncology

## Abstract

*Background/Objectives:* Colorectal cancer (CRC) remains a major global health burden, marked by complex tumor–microenvironment interactions, genetic heterogeneity, and varied treatment responses. Effective preclinical models are essential for dissecting CRC biology and guiding personalized therapeutic strategies. This review aims to critically evaluate current experimental CRC models, assessing their translational relevance, limitations, and potential for integration into precision oncology. *Methods:* A systematic literature search was conducted across PubMed, Scopus, and Web of Science, focusing on studies employing defined in vitro, in vivo, and emerging integrative CRC models. Studies were included based on experimental rigor and relevance to therapeutic or mechanistic investigation. Models were compared based on molecular fidelity, tumorigenic capacity, immune interactions, and predictive utility. *Results:* CRC models were classified into in vitro (2D cell lines, spheroids, patient-derived organoids), in vivo (murine, zebrafish, porcine, canine), and integrative platforms (tumor-on-chip systems, humanized mice, AI-augmented simulations). Traditional models offer accessibility and mechanistic insight, while advanced systems better mimic human tumor complexity, immune landscapes, and treatment response. Tumor-on-chip and AI-driven models show promise in simulating dynamic tumor behavior and predicting clinical outcomes. Cross-platform integration enhances translational validity and enables iterative model refinement. *Conclusions:* Strategic deployment of complementary CRC models is critical for advancing translational research. This review provides a roadmap for aligning model capabilities with specific research goals, advocating for integrated, patient-relevant systems to improve therapeutic development. Enhancing model fidelity and interoperability is key to accelerating the bench-to-bedside translation in colorectal cancer care.

## Simple Summary

Colorectal cancer, which affects the large intestine, is the second leading cause of cancer deaths worldwide, making it crucial to develop better treatments. However, researchers face a major challenge: the experimental models they use in laboratories to study this disease and test new treatments often do not accurately represent what happens in real patients. This comprehensive review examines the various laboratory methods scientists use to study colorectal cancer, including growing cancer cells in flat dishes, creating three-dimensional mini-tumors from patient samples, and testing treatments in laboratory mice. The study found that traditional flat cell cultures are convenient and inexpensive but fail to capture the complexity of actual tumors. Three-dimensional models and mini-tumors grown from patient tissue better mimic real cancer behavior, while animal studies provide valuable insights despite species differences. Importantly, no single experimental approach perfectly represents human colorectal cancer. The research concludes that combining multiple experimental methods provides the most comprehensive understanding of the disease. This work will help scientists choose the most appropriate laboratory models for their research, ultimately leading to more effective treatments that could save countless lives and improve outcomes for colorectal cancer patients worldwide.

## 1. Introduction

Colorectal cancer (CRC), encompassing malignancies of the colon and rectum, represents one of the most lethal neoplasms worldwide, ranking as the second leading cause of cancer-related mortality and the third most commonly diagnosed cancer globally, according to World Health Organization estimates [1].The American Cancer Association (ACS) estimates 152,810 new cases of colorectal cancer in 2024, with an estimated 53,010 deaths [2]; hence, highlighting the severity of the Cancer.

Although CRC is the second most fatal cancer, the rate of CRC does vary by region, with there being an eight-fold variation between countries. Interestingly, researchers have noted that the incidence and mortality of CRC are highest and have augmented severity in developed regions. With increased industrialization and development in countries, the CRC risk was observed to increase uniformly [3]. The highest incidence of CRC is in countries such as Australia, New Zealand, and Southern Europe, with age-standardized incidence rates (ASR) reaching up to 48.1 per 100,000 in Denmark [4]. Furthermore, CRC constitutes the leading cause of cancer-related mortality among males in several Middle Eastern nations, including Saudi Arabia, Oman, and the United Arab Emirates—a pattern attributable to a convergence of factors such as rapid urbanization, dietary westernization, sedentary lifestyles, and limited population-wide implementation of early screening programs, all of which amplify CRC incidence and delay detection [5]. These countries, while geographically situated in the Middle East, exhibit socioeconomic and lifestyle characteristics increasingly aligned with developed nations, thereby mirroring the epidemiological transition in CRC risk factors.

Individuals in developed countries tend to be accustomed to sedentary lifestyles and Western diets, and this has increased the age of the population. A sedentary lifestyle, which is characterized by a lifestyle with increased time spent sitting, has been linked as a risk factor for CRC. Physical activity reduces the risk for CRC in a dose-dependent manner, with a significant decrease of 20–25% noted in both men and women. However, this protective effect decreases in individuals accustomed to a sedentary lifestyle, typically observed in developed countries. In fact, there is a 54% increase in the risk of CRC and CRC survivors having a poor quality of life due to this sedentary lifestyle. Although the precise mechanistic pathways underlying this association remain to be fully elucidated, it is hypothesized that a constellation of interrelated factors—including visceral adiposity, chronic low-grade inflammation, insulin resistance, and psychosocial stress, may synergistically promote tumorigenesis. Collectively, these data underscore a robust and biologically plausible link between sedentary behavior and increased colorectal cancer burden [6].

In tandem with physical inactivity, adherence to a Western dietary pattern—characterized by high consumption of red and processed meats, refined sugars, and saturated fats—has been strongly implicated in the rising incidence of CRC across the high-income Middle Eastern regions. One of the most comprehensive studies to date, conducted by Mehta et al. within the Health Professionals Follow-up Study (HPFS) and the Nurses’ Health Study (NHS), analyzed dietary patterns and molecular tumor subtypes over three decades and found that high adherence to the Western diet significantly increased the risk of overall CRC (RR 1.31, 95% CI: 1.15–1.48; *p* < 0.0001), with particularly strong associations observed for tumors arising in the distal colon and rectum. Intriguingly, this dietary pattern appeared more strongly linked to molecular subtypes defined by the absence of mutations in KRAS and BRAF, low levels of CpG island methylation (CIMP-low/negative), and microsatellite stability (MSS)—hallmarks of the conventional adenoma-carcinoma pathway [7]. While formal statistical heterogeneity was not uniformly significant in this cohort, follow-up analyses, within the same population, reported significantly stronger protective associations between healthy plant-based diets and KRAS-wildtype tumors (HR = 0.74, 95% CI: 0.57–0.96; *p*-trend = 0.004; *p*-heterogeneity = 0.003), suggesting molecular subtype-specific dietary modulation [8]. The carcinogenicity of the Western diet is believed to be driven by a confluence of biological mechanisms. These include diet-induced gut microbiota dysbiosis—marked by reduced microbial diversity and diminished production of protective short-chain fatty acids (notably butyrate)—alongside increased microbial metabolism of proteins and bile acids into pro-inflammatory and genotoxic metabolites such as ammonia, hydrogen sulfide, and secondary bile acids like deoxycholic acid [9]. Additionally, cooking methods and preservatives inherent to the Western diet generate mutagenic compounds such as heterocyclic amines, polycyclic aromatic hydrocarbons, and N-nitroso compounds, which have been implicated in DNA alkylation, oxidative damage, and mutagenesis of driver genes including APC and TP53 [10]. These factors collectively promote chronic colonic inflammation, oxidative stress, mucosal barrier dysfunction, and a tumor-permissive immune microenvironment, setting the stage for neoplastic transformation and progression. Moreover, adherence to a Western diet often clusters with other adverse lifestyle factors—such as physical inactivity, tobacco use, central obesity, insulin resistance, and low uptake of CRC screening—thereby compounding cancer risk through both independent and synergistic mechanisms [11]. Studies using latent class analysis and behavior clustering models consistently demonstrate non-random co-occurrence of these risk factors, particularly in males. This aggregation of behaviors suggests that the Western lifestyle functions not as a single risk entity but as a systemic, multi-faceted pro-carcinogenic framework, exerting cumulative biological stress through converging pathways of inflammation, metabolic dysregulation, and immune evasion. This concept is further supported by gene—environment interaction analyses indicating a disproportionately amplified CRC risk in individuals adhering to a Western diet who also have a positive family history of CRC (RR up to 14-fold in individuals ≤ 55 years old). Taken together, these findings strongly implicate the Western dietary and behavioral profile as a central, modifiable determinant of CRC risk, with evidence suggesting preferential promotion of KRAS/BRAF-wildtype tumors. This underscores the urgent need for integrated public health strategies targeting dietary reform, physical activity, and lifestyle modification to interrupt this carcinogenic cascade and reduce the global burden of colorectal cancer.

Additionally, age is a well-established and significant risk factor for CRC, with the incidence traditionally increasing substantially as people get older. Many diagnoses occur in individuals over the age of 65 [3]. However, a concerning trend observed globally in recent decades is the significant and rapid rise in the incidence of early-onset colorectal cancer (EO-CRC), which is defined as CRC diagnosed in individuals younger than 50 years old [12]. This increase has been documented in numerous countries, and projections estimate further substantial increases in EO-CRC rates in the coming years. EO-CRC often presents differently than CRC diagnosed at older ages. It is more frequently located in the distal colon or rectum and may exhibit more aggressive features, often being diagnosed at a later, more advanced stage. The reasons for the rise in EO-CRC are still under investigation but are thought to be largely driven by changes in environmental exposures and lifestyle factors, including diet, starting earlier in life. Additionally, certain molecular characteristics of CRC can be associated with age. For instance, tumors with BRAF mutations are often found more commonly in older patients.

In addition to dietary patterns and advancing age, CRC risk is modulated by a constellation of non-dietary biological and sociodemographic factors, among which genetic and epigenetic alterations play a pivotal role. Hereditary syndromes account for 5–10% of CRC cases. The most common of these is Lynch syndrome (LS), also known as hereditary nonpolyposis colorectal cancer (HNPCC), which accounts for approximately 2–5% of all CRC cases. Caused by germline mutations in DNA mismatch repair (MMR) genes (most commonly MLH1, MSH2, MSH6, or PMS2), LS confers a dramatically elevated lifetime CRC risk, estimated at up to 80%, often leading to early-onset disease (average age 45–61). LS is also associated with increased risks for endometrial (up to 60% lifetime risk), ovarian, gastric, small bowel, urinary tract, and other cancers. Tumors in LS typically exhibit microsatellite instability (MSI) due to the MMR deficiency and often arise from an accelerated adenoma-to-carcinoma sequence. Familial adenomatous polyposis (FAP), responsible for about 1% of CRC cases, results from germline mutations in the APC gene. Classic FAP is characterized by the development of hundreds to thousands of colorectal adenomas, usually starting in adolescence, leading to a nearly 100% lifetime risk of CRC by age 40–45 if prophylactic colectomy is not performed. Attenuated FAP (AFAP) presents with fewer polyps (<100) and a later onset of CRC (mean age 50–58), carrying a lifetime risk of approximately 70%. Beyond these high-penetrance syndromes, common low-risk single nucleotide polymorphisms (SNPs) identified through genome-wide association studies (GWAS) have been associated with modest but cumulative increases in CRC susceptibility. Numerous susceptibility loci have been identified, including regions such as 8q24, 18q21 (SMAD7), 15q13.3 (GREM1), 20p12.3 (BMP2), 11q23.1, 14q22.2 (BMP4), and 16q22.1 (CDH1), often implicating pathways like TGF-β signaling. Epigenetic dysregulation, encompassing heritable changes in gene expression without altering the DNA sequence, also significantly contributes to CRC pathogenesis. Key mechanisms include global DNA hypomethylation, aberrant hypermethylation of CpG islands leading to silencing of tumor suppressor genes (e.g., MLH1 promoter silencing in sporadic MSI tumors), alterations in histone modifications (like acetylation and methylation), chromatin remodeling, and changes in non-coding RNA (e.g., microRNA) expression. A distinct molecular subtype, CIMP-high (CpG island methylator phenotype), characterized by extensive promoter methylation, is found in a subset of CRCs and is particularly associated with BRAF mutations, MSI-high status (often via MLH1 silencing), and proximal (right-sided) tumor location. Importantly, unlike genetic mutations, epigenetic alterations are potentially reversible, offering therapeutic targets.

Furthermore obesity, particularly visceral adiposity (excess fat stored around abdominal organs), is another well-established independent and modifiable risk factor. Recent meta-analyses confirm that obesity significantly increases CRC risk, potentially by 25–57% or more. Some research suggests that the contribution of excess weight to the CRC burden may be substantially underestimated (potentially accounting for over 20% of cases) if factors like pre-diagnostic weight loss and screening history are not considered. Visceral adipose tissue is metabolically active, acting as an endocrine organ that releases pro-inflammatory cytokines (e.g., TNF-α, IL-6, IL-8, MCP-1) and adipokines (e.g., leptin, resistin), while often showing reduced secretion of protective adiponectin. This contributes to a state of chronic low-grade systemic inflammation and insulin resistance, leading to elevated circulating levels of insulin and insulin-like growth factor-1 (IGF-1). These factors collectively promote cellular proliferation, angiogenesis (new blood vessel formation), and inhibition of apoptosis in colonic epithelial cells, creating a pro-tumorigenic environment. The adverse effects of obesity are further amplified by increased oxidative stress and alterations in bile acid metabolism, with mounting evidence supporting a role for obesity-driven gut dysbiosis in CRC pathogenesis. Specific signaling pathways implicated include STAT3, NF-κB, WNT/β-catenin, and PI3K/AKT/mTOR. While BMI is commonly used, measures of visceral fat may be more strongly correlated with CRC risk and outcomes.

Racial and ethnic disparities significantly influence CRC incidence, molecular characteristics, and clinical outcomes. In the United States, African Americans (or Black individuals) experience the highest CRC incidence and mortality rates compared to other major racial/ethnic groups. They are often diagnosed at more advanced stages and exhibit worse stage-specific survival. While disparities in access to screening and timely, high-quality care are major contributors to these outcomes, differences in tumor biology may also play a role. Tumors in Black patients show higher rates of proximal location, potentially lower rates of MSI-high status, higher frequency of KRAS mutations, and lower frequency of BRAF mutations compared to White patients. American Indian/Alaska Native (AIAN) populations also face disproportionately high CRC incidence and mortality. Hispanic and Asian/Pacific Islander (API) populations generally have lower rates than White individuals, but significant heterogeneity exists within these broad categories (e.g., Southeast Asians and Pacific Islanders may have higher risks or worse outcomes than East or South Asians). Emerging evidence also suggests that Arab populations may exhibit unique CRC molecular profiles, such as lower frequencies of SMAD4 mutations and higher frequencies of FBXW7 mutations compared to Western populations, although rates for key genes like KRAS, NRAS, BRAF, TP53, and PIK3CA appear largely similar. The increasing CRC incidence in many Arab nations is often linked to the adoption of Western lifestyles. The causes of these disparities are multifactorial, involving a complex interplay of socioeconomic determinants, access to care, environmental exposures, lifestyle factors, genetic variation, and epigenetic signatures.

Together, these diverse non-dietary factors—spanning inherited genetic mutations, somatic and epigenetic alterations, metabolic dysregulation due to obesity, and racial/ethnic disparities influenced by both biological and societal factors—underscore the multifaceted and personalized nature of colorectal cancer risk and pathogenesis. This highlights the critical need for stratified prevention strategies, equitable access to screening and care, and tailored surveillance and therapeutic approaches. Against this complex and evolving landscape, there is a pressing need to re-examine the experimental models used to investigate CRC biology, therapeutic vulnerabilities, and resistance mechanisms. This review critically appraises existing preclinical CRC models and offers a forward-facing roadmap for their refinement and translational optimization.

While these diverse factors shape CRC incidence and molecular profiles, they also profoundly influence treatment response and resistance—underscoring the need for models that can faithfully capture this heterogeneity. Despite substantial progress in screening and surgical management, CRC continues to pose significant therapeutic challenges, particularly in the metastatic setting, where long-term survival remains elusive. First-line treatment for advanced CRC typically involves combination chemotherapy regimens, including FOLFOX (5-fluorouracil, leucovorin, and oxaliplatin) and FOLFIRI (5-fluorouracil, leucovorin, and irinotecan), often administered with targeted biologics such as the VEGF-A inhibitor bevacizumab or the EGFR inhibitors cetuximab and panitumumab, the latter restricted to RAS wild-type tumors. More recently, immune checkpoint inhibitors (e.g., pembrolizumab, nivolumab) have shown durable efficacy in MSI-high or MMR-deficient tumors, yet this molecular subtype constitutes only a small proportion of sporadic CRC. Patients with KRAS, NRAS, or BRAF mutations often exhibit primary resistance to EGFR-targeted therapies, while those with CMS4 (mesenchymal) tumors remain largely refractory to most existing agents. Additionally, the therapeutic landscape is further complicated by adaptive resistance mechanisms, tumor heterogeneity, and the immunosuppressive tumor microenvironment (TME), which collectively blunt therapeutic responses. Although promising avenues such as gene therapy, antisense oligonucleotides, oncolytic virotherapy, and tumor-agnostic immunotherapies are under investigation, their clinical translation is hindered by the lack of experimental systems that faithfully replicate human CRC biology, particularly its molecular evolution, immune landscape, and metastatic behavior.

This complex clinical and molecular milieu underscores the critical need for refined and stratified preclinical models of CRC. However, current models—ranging from long-established 2D monolayer cell lines to patient-derived xenografts (PDXs), genetically engineered mouse models (GEMMs), organoids, and alternative in vivo systems such as zebrafish and porcine models—present substantial limitations. Traditional 2D cell lines, while accessible and high-throughput, often lack in vivo-like tissue architecture, stromal interaction, and intratumoural heterogeneity. PDXs and GEMMs offer more faithful recapitulation of native tumor biology but are constrained by cost, time, low throughput, species-specific immune incompatibilities, and limited capacity to model dynamic tumor-immune interactions or treatment-induced clonal evolution. Organoids capture patient-specific genetic and phenotypic traits but often fall short in mimicking the immune microenvironment and metastatic potential. Most importantly, across all these platforms, there is a widespread lack of standardization, poor integration of multi-omics data, and limited scalability for drug screening, especially for combinatorial or immunotherapeutic strategies.

To address these deficiencies, there is an urgent imperative to redefine CRC model systems through multidimensional enhancements. This includes incorporating patient-matched stromal and immune components via co-culture platforms, employing humanized mouse systems for immuno-oncology studies, and leveraging high-throughput CRISPR/Cas9-based genome editing to replicate specific molecular lesions across distinct subtypes. Furthermore, the application of artificial intelligence (AI) and machine learning to CRC model development offers powerful avenues for pattern recognition, multi-omic integration, and prediction of therapeutic response. AI-driven platforms can help optimize experimental design, identify novel biomarkers, and even simulate tumor evolution under therapeutic pressure, thereby reducing experimental redundancy and accelerating translational relevance. Additionally, coupling spatial transcriptomics and single-cell sequencing with organoid and PDX models may allow for deeper insights into cellular plasticity, metastatic niche colonization, and therapy-induced resistance.

In this review, we present a critical, translationally anchored evaluation of existing colorectal cancer model systems, highlighting both their contributions and constraints in addressing current therapeutic challenges. We map these models onto the evolving clinical landscape, identify methodological gaps, and outline strategies to enhance their biological fidelity and translational utility. In doing so, we seek to provide a roadmap for researchers and clinician-scientists striving to bridge the preclinical–clinical divide, enabling more precise, immune-aware, and mechanism-driven interventions for one of the world’s most formidable malignancies.

### Classification of Colorectal Cancer Models

Experimental models of CRC have evolved significantly over the past decades, ranging from immortalized 2D cell lines to sophisticated organoid cultures, patient-derived xenografts (PDXs), genetically engineered mouse models (GEMMs), and emerging in vivo systems such as zebrafish and porcine models. These models vary substantially in terms of their complexity, fidelity to human tumor biology, capacity for genetic manipulation, suitability for high-throughput drug screening, and ability to recapitulate tumor–microenvironment interactions. For the purposes of this review, CRC models may be broadly classified into: (i) **in vitro models**, including two-dimensional cell lines, three-dimensional spheroids, and patient-derived organoids; (ii) **in vivo models**, encompassing, animal, xenografts, syngeneic models, and transgenic animals; and (iii) **emerging integrative platforms**, which include humanized models, microfluidic tumor-on-chip systems, AI-augmented computational frameworks and cross-scale integrated models. A detailed comparative analysis of each category—**in vitro**, **in vivo**, and **emerging integrative platforms**—is presented in Table 1, Table 2, and Table 3, respectively, highlighting their relative strengths, limitations, and translational utility in colorectal cancer research. This classification allows for a systematic and comparative evaluation of their respective advantages, limitations, and applications in translational research and therapeutic development.

## 2. Methodology

To ensure a comprehensive and analytically robust evaluation of preclinical CRC models, a systematic literature review was conducted in accordance with established best practices for biomedical reviews. The review aimed to capture the landscape of in vitro and in vivo CRC models—including two-dimensional (2D) cell lines, three-dimensional (3D) spheroids, patient-derived organoids (PDOs), and murine systems such as patient-derived xenografts (PDXs) and genetically engineered mouse models (GEMMs)—and assess their utility in translational oncology.

The literature search was executed across three major biomedical databases: PubMed/MEDLINE, Scopus, and Web of Science. The search strategy incorporated controlled vocabulary (e.g., MeSH terms) and free-text keywords to maximize both sensitivity and specificity. Primary search terms included: “colorectal cancer models,” “CRC cell lines,” “colorectal organoids,” “CRC spheroids,” “patient-derived xenografts,” “GEMMs,” and “tumor microenvironment.” These were strategically combined with mechanistic and translational terms such as “drug resistance,” “chemotherapy response,” “Wnt/β-catenin pathway,” “epithelial–mesenchymal transition,” “precision medicine,” and “immune-oncology,” using Boolean operators (AND, OR, NOT) to optimize query performance. The final search encompassed publications indexed up to January 2025 and was limited to English-language, peer-reviewed research articles.

Study selection followed a rigorous multi-step filtration process. Titles and abstracts were initially screened to exclude irrelevant records. Full-text articles were subsequently retrieved for comprehensive eligibility assessment. Inclusion criteria mandated that studies utilize authentic CRC models with defined experimental protocols—either in vitro (2D or 3D systems) or in vivo (PDX, xenograft, or GEMM). Only articles demonstrating mechanistic investigation, therapeutic interrogation, or biomarker analysis were retained. Exclusion criteria encompassed non-English publications, case reports, editorials, conference abstracts, and studies lacking experimental validation.

For data extraction and synthesis, studies were stratified into thematic categories: (i) classical 2D cell lines, (ii) 3D spheroids, (iii) PDOs, and (iv) in vivo models. Each model type was assessed for its derivation, molecular fidelity, growth kinetics, tumorigenic potential, and fidelity to the parent tumor microenvironment. Additional emphasis was placed on genetic alterations (e.g., MSI status, APC/KRAS/TP53 mutations), pharmacologic response profiles, and phenotypic plasticity. Data from high-throughput drug screens, CRISPR/Cas9 perturbation assays, RNA interference studies, and co-culture experiments were also extracted where applicable.

Furthermore, emerging domains such as microfluidic tumor-on-chip systems, AI-enhanced pharmacogenomics, and integrative omics platforms were appraised to delineate the evolving frontier of CRC modeling. The analytical framework employed a narrative synthesis approach, supported by comparative data matrices and schematic representations to enhance interpretability. Visual elements, including curated tables and illustrative figures, were used to juxtapose features across model systems and highlight translational applicability.

All included studies were critically appraised for methodological robustness, reproducibility, and clinical relevance. Priority was given to recent publications employing next-generation sequencing, high-content imaging, and systems biology approaches. Particular attention was paid to model fidelity, capacity to recapitulate intratumoural heterogeneity, and their utility in guiding patient-specific therapeutic strategies.

This methodological framework ensured a high-resolution mapping of the CRC model ecosystem, enabling a critical appraisal of current limitations, knowledge gaps, and future opportunities in precision oncology.

All figures presented in this manuscript are original creations developed by the authors using BioRender.com (BioRender Version 2024.3), adhering to institutional licensing agreements and figure preparation guidelines. None of the figures presented in this manuscript are copyrighted or have been replicated from external sources.

### 2.1. In Vitro Models

#### Two-Dimensional (2D) Colorectal Cancer Cell Line Models: Clinical Utility, Molecular Features, and Limitations

**Figure 1:**
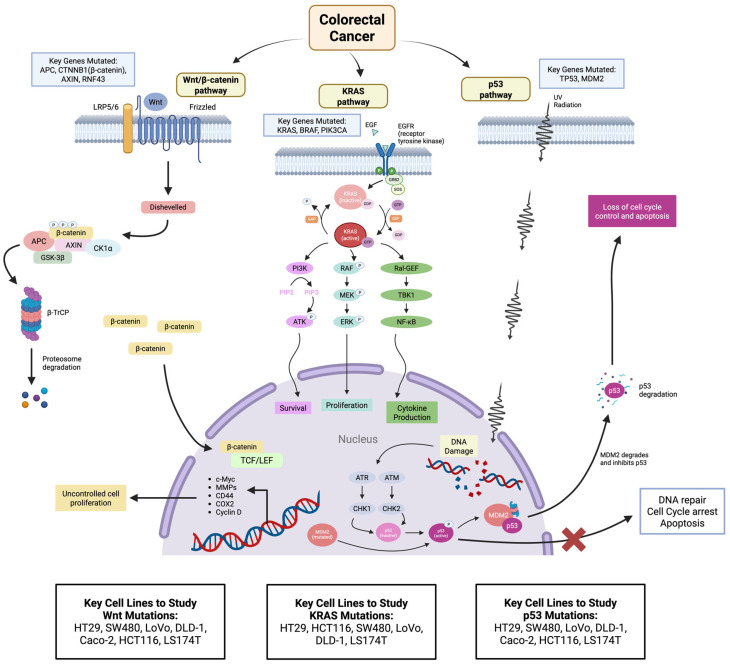
**Molecular Pathways Dysregulated by Recurrent Colorectal Cancer Driver Mutations**. This schematic highlights the mechanistic dysregulation of three core CRC pathways—Wnt/β-catenin, KRAS/MAPK, and p53 and emphasizes the key cell lines that best model mutations in these pathways. These cell lines serve as essential tools for studying the downstream effects of recurrent driver mutations and enable pathway-specific experimental investigation.

***Molecular stratification***: The seven commonly used CRC cell lines—HT29, HCT116, SW480, LoVo, DLD-1, Caco-2, and LS174T—collectively encompass the major molecular subtypes of CRC (Table 4). Each line carries a distinct constellation of driver mutations and microsatellite stability (MSS) or instability (MSI) status that stratify them into different CRC molecular categories (Figure 1). For example, HT29 carries a BRAF^V600E^ mutation along with mutated TP53, whereas HCT116 harbors an activating KRAS^G13D^ mutation and an MLH1 mismatch repair gene defect (MSI-high phenotype) [7]. DLD-1 is similarly MSI-high with mutant KRAS, while SW480 (derived from a primary tumor) is MSS with mutant KRAS and APC and a loss-of-function TP53 mutation. Caco-2, in contrast, is MSS and KRAS/BRAF wild-type, characterized by an APC mutation and intact TP53 LS174T features mutations in KRAS and APC and is MSS. These genetic differences align each cell line with specific molecular stratifications of CRC. Notably, the MSI-high lines (HCT116, DLD-1, LoVo) model hypermutated tumors akin to those in the CMS1 (MSI-Immune) Consensus Molecular Subtype, which are often proximal colon cancers with high immunogenicity. In contrast, the MSS lines like SW480 and Caco-2 represent the chromosomally unstable, Wnt-driven subtype common in sporadic left-sided CRC (overlapping with CMS2/3). HT29 is an interesting case: despite being MSS, its BRAF mutation links it to the poor-prognosis subgroup of BRAF-mutant tumors (often associated with MSI in patients)—thus HT29 serves as a surrogate for that molecular class in an MSS setting.

Each cell line’s molecular profile also confers unique biomarkers; for instance, HCT116 is mismatch repair-deficient and MLH1-null but retains wild-type p53, whereas HT29 and DLD-1 have mutated TP53, reflecting the general observation that MSI CRCs often have intact p53 while MSS lines typically harbor p53 loss-of-function mutations. This spectrum of oncogenic alterations and stability statuses across the seven lines allows researchers to molecularly stratify experimental models to mirror the heterogeneity of CRC. Furthermore, gene expression-based subtyping confirms that these lines span the Consensus Molecular Subtypes: MSI-high lines like HCT116 and LoVo tend toward an “undifferentiated/mesenchymal” expression profile (CMS1/CMS4) while Caco-2 and similar MSS lines display “epithelial/colon-like” features (CMS2/CMS3). Such stratification is crucial, as it correlates with pathway activations (e.g., WNT, EGFR/MAPK, PI3K) and allows selection of appropriate in vitro models for a given molecular inquiry.

***Consensus molecular subtypes (CMS) relevance***: The diversity of these cell lines enables alignment with CMS classifications, which has implications for research translation. *CMS1 (MSI-Immune)* tumors are represented by HCT116, LoVo, and DLD-1, which are dMMR (defective DNA mismatch repair) and display hypermutation. Consistent with patient tumors in CMS1, these lines harbour numerous mutations (including frameshifts) and typically present with an “inflamed” gene signature; in fact, LoVo and HCT116 tend to exhibit a more mesenchymal, undifferentiated morphology in culture, mirroring the immune-associated but EMT-prone phenotype of CMS1 cancers.

*CMS2 (Canonical)*, characterized by Wnt/MYC activation and chromosomal instability, is exemplified by lines like SW480 and Caco-2. SW480 is APC-mutant and highly aneuploid, with a strongly epithelial morphology, reflecting the prototypical CMS2 tumor that is microsatellite-stable and driven by Wnt signaling. Caco-2, although derived from a primary tumor, also fits a colon epithelial (CMS2/CMS3) profile, evidenced by its ability to spontaneously differentiate.

*CMS3 (Metabolic) subtype*, often KRAS-mutant and displaying a mixed differentiation pattern, is typified by LS174T and DLD-1. LS174T is a mucinous CRC line (producing copious mucus in culture) with mutant KRAS—features that align with the mucinous, metabolically reprogrammed CMS3 tumors which frequently harbour KRAS mutations. DLD-1, with mutant KRAS and MSI, straddles CMS1 and CMS3 features, representing an overlap (it arises from a hypermutated tumor but with a metabolic gene expression tilt due to KRAS mutation).

*CMS4 (Mesenchymal)*, characterized by EMT and stromal infiltration in tumors, is partially represented in vitro by the propensity of certain lines to undergo mesenchymal transition. While no single 2D line fully recapitulates CMS4 (since stromal components are absent), SW480 can acquire mesenchymal traits in subpopulations, and LoVo has a fibroblast-like appearance and invasive behavior in culture, resonating with CMS4 features. By mapping these cell lines to CMS and related classifications, researchers can select appropriate models—for instance, using HCT116 or DLD-1 for studies on immunogenic microsatellite-unstable CRC versus SW480 or HT29 for canonical pathway-driven CRC—thereby improving the clinical relevance of in vitro findings.

**Figure 2:**
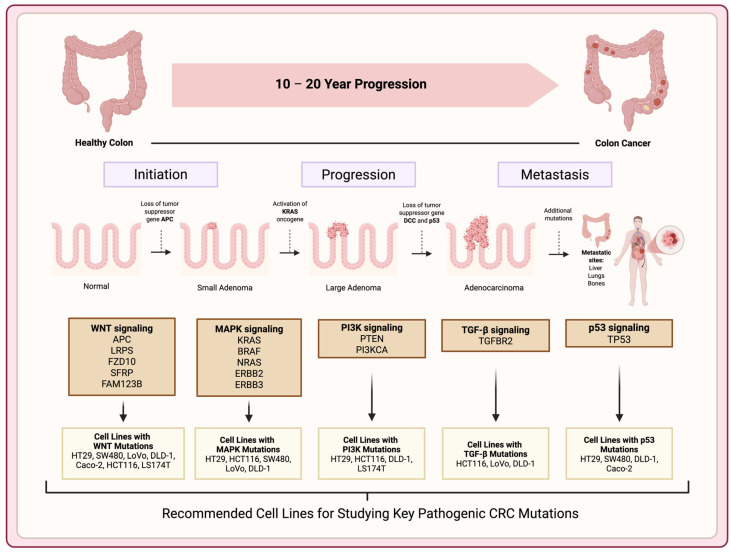
**Cell Line Mapping of Colorectal Cancer Progression Through Driver Mutations.** This diagram illustrates the progression of CRC from healthy colon epithelium to metastasis, highlighting key driver mutations and the cell lines best suited to model each stage. It connects specific mutations to pathway activations described in Figure 1. Notably, the signaling pathways detailed in Figure 1 are central to this progression, as mutations in genes like APC initiate the process via Wnt/β-catenin activation, followed by KRAS mutations driving further progression through MAPK signaling. Accumulation of mutations, including TP53 loss, ultimately leads to metastasis, demonstrating the interconnected roles of these pathways in the multistep development of CRC. The figure provides a resource for selecting appropriate cell lines when studying the chronological molecular events in CRC tumorigenesis.

***Clinical correlation***: Each of these 2D models bears a relationship to specific clinical subsets of CRC, enhancing their utility as patient proxies. This evolutionary framework of colorectal tumorigenesis—from normal epithelium through adenoma to carcinoma and metastasis—can be visually mapped using representative CRC cell lines that model key genetic milestones (Figure 2), thereby providing a functional scaffold for experimental studies of cancer progression.

*Tumor origin and stage*: Some lines derive from primary tumors, while others come from metastatic lesions, mirroring the progression of disease (Table 4). SW480 was established from a primary colon carcinoma (Dukes’ B stage), whereas LoVo and DLD-1 originate from metastatic sites (LoVo from a lymph node metastasis, DLD-1 from a Duke’s D stage tumor). LS174T is also derived from a metastatic ascites tumor and is notable for its production of carcinoembryonic antigen and mucin, features often seen in advanced, mucus-rich colon cancers. These origins correlate with the phenotypes: SW480, from an early-stage tumor, has an epithelial morphology and forms cohesive colonies, modeling early-stage carcinomas, while LoVo, from an advanced metastasis, is highly invasive and migratory in vitro, making it a suitable model for studying metastatic colon cancer. LoVo’s aggressive behavior (e.g., high anchorage-independent growth and invasion through matrices) correlates with its clinical background as a rapidly progressive tumor. Similarly, HT29 and Caco-2 are from primary colorectal tumors; HT29 is a grade II adenocarcinoma of the colon, and Caco-2 comes from a primary colon adenocarcinoma. Caco-2’s propensity to differentiate into mature enterocyte-like cells (forming tight junctions and microvilli in culture) reflects the well-differentiated status of the tumor it came from. In contrast, HT29’s original tumor was a moderately differentiated, mucin-producing colon cancer; accordingly, HT29 cells secrete mucin and display partial goblet cell features in vitro. Clinically, mucinous colorectal tumors (like the one HT29 models) often have distinct behavior and drug responses, and HT29’s mucin production and MUC gene expression make it a valuable in vitro surrogate for this subtype. The microsatellite status of these lines also has clinical parallels: HCT116, DLD-1, and LoVo are MSI-high, corresponding to the ~15% of CRC patients with MSI tumors (often right-sided and carrying better prognosis and responsiveness to immunotherapy). These lines exhibit high mutation burden and impaired DNA mismatch repair, just as patient MSI tumors do, and they are used to explore the unique features of MSI CRC (e.g., sensitivity to immune checkpoint blockade or resistance to certain chemotherapies). On the other hand, MSS lines like SW480, HT29, and LS174T model the more common chromosomally unstable CRCs, which typically have worse baseline prognosis but can respond to cytotoxic chemotherapy; indeed, HT29 and LS174T are both known to be relatively chemoresistant in vitro [30], echoing how many MSS tumors require aggressive therapy.

*Tumorigenicity and growth*: In vivo tumorigenic potential of these cell lines correlates with clinical aggressiveness. HCT116 and HT29 form tumors efficiently in xenografts (classified as highly tumorigenic [31]), reflecting the aggressive growth of the cancers they model, whereas Caco-2 is essentially non-tumorigenic in mice (it rarely forms tumors), corresponding to its origin as a less aggressive tumor and its strong differentiation in vitro. LoVo and LS174T have intermediate tumorigenicity [32]—they will form tumors in mice but with moderate latency—aligning with their moderate clinical malignancy (mucinous and MSI tumors can be locally aggressive yet somewhat less prone to forming large masses). These correlations bolster confidence that such cell lines can approximate the patient experience: for instance, therapies that suppress tumor growth in HT29 xenografts (a highly tumorigenic, chemoresistant line) might hold promise for treating refractory, aggressive CRC in patients [33]. Additionally, the lines’ tissue of origin (all colon adenocarcinomas) and, in some cases, site specificity, allow disease-relevant modeling—e.g., Caco-2 and HT29 are often used as in vitro models of colon epithelium in inflammatory bowel disease-associated CRC studies because they maintain features of colonic epithelial differentiation and barrier function. Notably, in our recent investigations, we utilized both HT29 and Caco-2 cells to explore whether atorvastatin and rosuvastatin, as well as the nutraceuticals oleocanthal and curcumin, can attenuate protease-activated receptor-2 (PAR-2)–initiated pro-inflammatory signaling. These cell lines provided a robust and reproducible platform to assess drug- and nutraceutical-mediated modulation of epithelial inflammation, allowing interrogation of molecular mediators under inflammatory stress conditions [34]. In summary, by matching cell line characteristics with clinical tumor subtypes (primary vs. metastasis, MSI vs. MSS, mucinous vs. non-mucinous, etc.), researchers achieve a clinical correlation that makes experimental findings more translatable to patient contexts.

**Figure 3:**
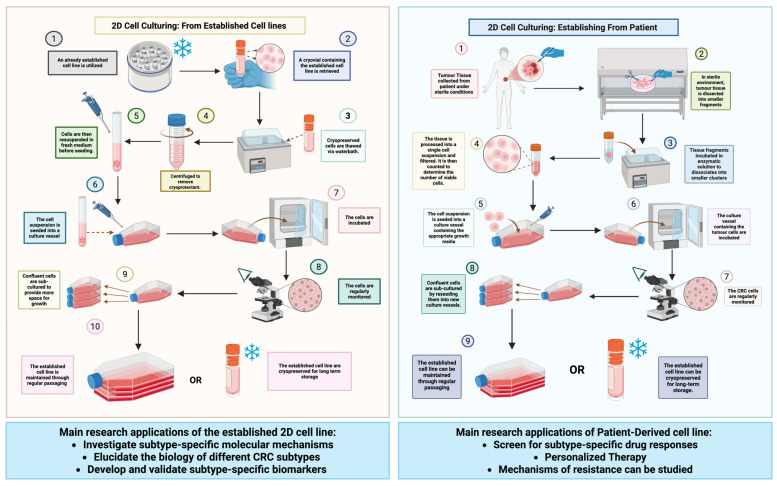
**Culturing Workflow and Applications of Two-Dimensional Colorectal Cancer Cell Lines and Patient-Derived Cell Lines.** This figure presents a stepwise overview of culturing techniques for both established colorectal cancer cell lines and patient-derived cell lines, highlighting key differences in sourcing, handling, and scalability. Major scientific applications are also described, including the development of biomarkers, the investigation of subtype-specific molecular mechanisms, and the testing of personalized therapies. For the investigation of CRC subtypes and treatment targeting, these models provide easily accessible and reproducible platforms.

***Therapeutic applications:*** The genomic and phenotypic diversity of these 2D CRC models underpins their extensive use in testing therapeutic strategies and identifying drug vulnerabilities. A stepwise overview of culturing approaches for both established and patient-derived CRC cell lines, along with their translational applications in biomarker discovery and therapy development, is presented in Figure 3. In preclinical drug development, panels of these cell lines are employed to represent the spectrum of CRC and to evaluate treatment efficacy across different molecular backgrounds [35]. For example, anti-EGFR monoclonal antibody therapies (like cetuximab and panitumumab) are only effective in CRCs without RAS or BRAF mutations [36]. Among these lines, Caco-2 (KRAS/BRAF wild-type) serves as a model predicted to be sensitive to EGFR inhibition, whereas lines with KRAS mutations (HCT116, DLD-1, SW480, LS174T) or a BRAF mutation (HT29) inherently resist EGFR-targeted treatment, mirroring clinical outcomes where KRAS/NRAS or BRAF mutations confer resistance to EGFR inhibitors [37]. Indeed, drug response assays have shown that KRAS-mutant lines like DLD-1 do not respond to EGFR blockade, validating these cells as robust models of primary resistance. HT29, with BRAF^V600E^, has been pivotal in testing BRAF-targeted therapies; studies using HT29 revealed that single-agent BRAF inhibitors have limited effect due to feedback activation of EGFR, leading to the development of combination therapies (EGFR/BRAF or MEK/BRAF inhibitors) for BRAF-mutant CRC in the clinic [38]. Likewise, HCT116 and DLD-1 (both KRAS^G13D^, MSI-high) are frequently used to trial MEK or ERK inhibitors and to explore combinations that might overcome Ras-mediated drug resistance. These models have also been instrumental in studying cytotoxic chemotherapy responses: for instance, a panel including HCT116, DLD-1, and HT29 helped establish that mismatch repair-deficient lines are relatively resistant to fluorouracil-based chemotherapy, whereas proficient lines undergo more apoptosis—a finding that aligned with clinical observations that MSI-high tumors have distinct 5-FU response profiles [39]. The unique vulnerabilities of each line can be exploited to discover targeted treatments. HCT116, LoVo, and DLD-1 share a dependency on the WRN helicase for survival due to their MSI status—large-scale CRISPR screens identified WRN as a synthetic lethal target in MSI CRC cells [40]. This discovery, made in vitro, is now informing the development of therapies specifically for dMMR tumors. Similarly, lines with specific pathway activations are used to test pathway inhibitors: SW480 (APC and KRAS mutant) is used to probe Wnt and MAPK pathway inhibitors, while LS174T (which produces high levels of mucin and expresses immune checkpoint molecules) is used to evaluate interventions targeting the tumor–immune interface, such as CAR-T cell targeting of tumor antigens or modulators of immune checkpoints [41]. The value of these cell lines is further amplified when they are used in combination as a composite panel. By testing a new drug across all seven lines (and often additional CRC lines), researchers can determine if its efficacy is broad-based or restricted to certain genetic contexts. For example, a hypothetic AKT inhibitor might show potent growth suppression in PIK3CA-mutant lines (HT29, HCT116) but not in others, indicating a genotype-specific effect. Such differential responses guide patient stratification in clinical trials. Indeed, high-impact studies have screened dozens of CRC cell lines (including the ones discussed) to correlate mutations with drug sensitivities (As an illustration, the NCI-60 panel (which includes HT29 and HCT116) was used to identify histone deacetylase inhibitors as broadly active in colon cancer models, leading to clinical exploration of these agents [42]. In continuation with this translational framework, our studies employing atorvastatin, rosuvastatin, oleocanthal, and curcumin utilized a dual pro-inflammatory epithelial model comprising HT29 and Caco-2 cells. These lines, chosen for their preserved epithelial characteristics and responsiveness to cytokine-driven inflammation, served as robust platforms to interrogate Protease-Activated Receptor (PAR) signaling. Notably, these therapeutic agents selectively attenuated the expression of PAR-2 while sparing PAR-1, highlighting a degree of target specificity that underscores their potential in modulating PAR-2–dependent inflammatory cascades [43]. This observation carries significant biomarker implications, particularly in contexts where PAR-2 serves as a sentinel of chronic epithelial inflammation. Moreover, it illustrates the versatility of the HT29–Caco-2 dual model for dissecting receptor-level pharmacodynamics and for screening agents with selective anti-inflammatory potential in colonic epithelial settings. In practice, these 2D models are often the first-line screening platforms for drug discovery in CRC: they are easy to culture, reproducible, and amenable to high-throughput assays. Promising therapies (e.g., kinase inhibitors, oncolytic viruses, immunotoxins) can be tested on this panel to gather initial efficacy and selectivity data before moving into more complex 3D organoids or in vivo models [44]. In summary, the therapeutic applications of these cell lines range from validating targeted therapy strategies (such as the need for dual EGFR/BRAF inhibition in BRAF-mutant HT29 models) to uncovering novel drug targets (such as WRN in MSI models) and establishing pharmacogenomic relationships that inform precision medicine in CRC.

***Use in inflammation and resistance research***: Beyond standard drug efficacy studies, 2D CRC cell lines are invaluable for mechanistic studies of tumor biology—notably, investigating how tumors interact with inflammatory signals and how they develop drug resistance. Inflammation is a known driver of colorectal carcinogenesis (e.g., colitis-associated cancer), and certain cell lines are particularly suited to model this interface. HT29 and Caco-2 stand out for studies of tumor–immune interactions and inflammatory responses because they express relatively high levels of Toll-like receptor 4 (TLR4) and other innate immune receptors [45]. HT29, in particular, has been used to model the effect of bacterial lipopolysaccharide (LPS) on colonic epithelial cells: upon LPS exposure, HT29 mounts a robust NF-κB-mediated inflammatory response, mirroring the behavior of CRC cells in a pro-inflammatory microenvironment. Caco-2, which differentiates into an enterocyte-like monolayer, is also used to study barrier function and how inflammatory cytokines (like TNF-α or IL-1β) disrupt epithelial tight junctions—processes relevant to both inflammatory bowel disease and CRC. Additionally, the high mitochondrial content observed in HT29 and Caco-2 [46] is leveraged in metabolic-inflammation research, as these lines can demonstrate how inflammatory conditions alter cancer cell metabolism (e.g., reactive oxygen species production, Warburg effect modulation). On the other hand, lines like HCT116 have slightly lower TLR4 and inflammatory cytokine expression, serving as a comparison to delineate line-specific responses to immune stimuli [47]. Using these cell models, researchers have shown that chronic exposure to inflammatory signals can induce epithelial–mesenchymal transition (EMT) and invasive behavior in vitro, connecting inflammation to metastasis—for instance, long-term TNF-α treatment of SW480 or HT29 cells elevates mesenchymal markers, simulating how cancer cells adapt in an inflamed tumor microenvironment. The panel of cell lines also enables dissection of drug resistance mechanisms in CRC. Reproducing acquired resistance in patients (which often emerges through selection of resistant clones or additional mutations) can be achieved in vitro by continuous drug exposure. Investigators have developed oxaliplatin- or 5FU-resistant sublines of HCT116 and DLD-1 to identify genetic changes conferring resistance (e.g., upregulation of thymidylate synthase or mutations in drug targets). Intrinsic resistance mechanisms can likewise be studied: HT29’s noted chemoresistance (it is relatively refractory to apoptosis by many standard agents [48] has been attributed to its high expression of anti-apoptotic proteins (BCL-2, BCL-XL) and mucus layer hindering drug penetration, making it a model for testing combination treatments that sensitize tumors to apoptosis. Another example is LS174T, which carries a mutation in B2M (beta-2-microglobulin) resulting in loss of surface MHC class I expression—immune evasion mechanism tumors use to escape cytotoxic T lymphocytes. LS174T’s B2M-null status and resultant inability to present antigens have made it a valuable tool for immune resistance studies, such as evaluating the efficacy of T cell-mediated therapies or interferon-γ induced upregulation of alternative antigen presentation pathways [49]. Co-culture experiments with immune cells (like CTLs or CAR-T cells) against LS174T help researchers understand how MHC-I–deficient cancers may still be targeted, informing strategies to overcome immune escape in MSI/MSS tumors (for instance, via NK cell activation or bispecific antibodies). Similarly, the MSI-high lines (HCT116, DLD-1, LoVo) are models for analyzing resistance to immune checkpoint inhibitors. While these drugs don’t act directly on the cancer cells in vitro, treatment of MSI line xenografts with PD-1/PD-L1 blockade in humanized mouse models has shown tumor regression, reflecting the clinical responsiveness of MSI CRC—thus these cell lines underpin in vivo studies of immunotherapy resistance when factors like loss of antigen presentation (e.g., via B2M mutations, as in LS174T) occur. In the realm of targeted therapy resistance, SW480 has been illustrative: it contains subclones with varying KRAS pathway activation [50], and upon exposure to EGFR inhibitors, SW480 readily selects for clones with EGFR pathway-independent growth, mirroring acquired resistance seen in patients with EGFR blockade. By comparing SW480 to its isogenic metastatic derivative SW620 (not among the seven, but closely related), researchers also study how additional alterations (SW620, from a metastasis, has further mesenchymal traits) confer resistance to therapies that SW480 (primary) is initially sensitive to, recapitulating the stepwise resistance that can occur as CRC progresses. In summary, these 2D models are indispensable for interrogating how CRC cells interact with inflammatory environments and how they evade therapeutic pressures. They allow controlled experiments to unravel pathways of immune evasion (like antigen loss or cytokine signaling loops) and drug resistance (via mutation, amplification, or phenotypic shifts), thereby providing mechanistic insights that inform the development of combination therapies to pre-empt or overcome resistance.

***Limitations of two-dimensional colorectal cancer models***: Despite their widespread utility, conventional 2D CRC cell lines have well-recognized limitations that must be acknowledged.

Foremost, they fail to fully recapitulate the complexity of actual tumors. Tumors in vivo are three-dimensional, heterogeneous tissues composed not only of cancer cells but also stromal fibroblasts, immune infiltrates, extracellular matrix, and gradients of nutrients and oxygen—factors largely absent in 2D monolayer cultures. As a result, drug responses observed in cell lines do not always predict clinical efficacy [51]. CRC cell lines grown on plastic may overestimate the impact of a cytotoxic drug (since real tumors have regions of limited drug penetration and quiescent cells) or conversely may not reflect drug activation by stroma or immune-mediated cell killing. The simplistic microenvironment in vitro means that crucial tumor–stroma interactions, which can affect drug sensitivity and metastasis, are missing [52]. For example, a MEK inhibitor might completely suppress ERK signaling in a cell line, but in a tumor, cancer-associated fibroblasts might provide alternative survival signals—such context is not captured in a monoculture.

Another limitation is the genetic drift and clonal selection that cell lines undergo during prolonged culture. Over years in vitro, cell lines can acquire new mutations or epigenetic changes that diverge from the original patient tumor genotype [53]. This evolution can lead to discrepancies: a cell line might become less differentiated or more proliferative than the tumor it was derived from. Studies have noted that aneuploid CRC lines like HT29 and SW480 continue to genetically evolve in culture, generating subclonal diversity [54]. While this can be viewed as a model of tumor heterogeneity, it also means that different labs might observe slightly different behaviors from what is ostensibly the “same” cell line. Cross-contamination and misidentification have historically been an issue as well (though improved authentication has mitigated this).

Furthermore, as clonal populations, cell lines lack the intratumoral heterogeneity of patient cancers—the minor subpopulations with distinct drug sensitivities or metastatic propensities are not present unless deliberately co-cultured. This can make a drug seem uniformly effective in vitro, whereas in a patient, a small resistant clone could survive and cause relapse.

Lack of differentiation and architecture is another shortcoming. Most CRC cell lines (with the notable exception of Caco-2) do not undergo significant differentiation in standard 2D culture; they often represent a more dedifferentiated state than the primary tumor. They grow as immortalized, often hyper-proliferative monolayers that do not form the glandular structures characteristic of colorectal tumors. Thus, processes that depend on 3D architecture—like cell polarity, lumen formation, and gradient-dependent drug diffusion—are not accurately modeled. Additionally, 2D cultures exhibit artificial accessibility of nutrients and drugs (every cell is equally bathed in medium), which can overestimate efficacy and underestimate the importance of penetration barriers.

The absence of a functional tumor microenvironment (TME) in 2D cultures is particularly pertinent for studying immunotherapies and angiogenesis inhibitors, which require the presence of immune cells and blood vessels, respectively [55]. Without TME components, 2D cell lines cannot capture immune checkpoint regulation (beyond intrinsic cancer cell expression of PD-L1) or the role of tumor vasculature in drug delivery.

Despite these limitations, researchers address some of these gaps by using complementary models: for instance, findings in 2D cell lines are increasingly validated in 3D patient-derived organoids or spheroids, which better preserve tumor architecture, and in xenograft models, which reintroduce a stromal context [56]. The 2D lines often serve as a necessary first filter—given their ease of use and low cost—but not the final evidence for translational research. It is now common to test a hypothesis or drug in a panel of cell lines, then confirm key results in organoids or in vivo. Another way to mitigate the limitations is by co-culturing cell lines with stromal cells or immune cells (for example, mixing CRC cell lines with cancer-associated fibroblasts or T cells in transwell systems), to partially recreate tumor microenvironment conditions. Still, even with such measures, 2D models cannot fully emulate tumor physiology. Thus, while the clinical utility of these CRC cell lines is unquestionable for hypothesis generation and mechanistic insight, their predictive power for clinical efficacy is limited. This is exemplified by the high attrition of drugs that showed promise in cell lines but failed in patients [57]. Recognizing these limitations, the field has moved toward integrating 2D cell line work with more advanced models. In contemporary CRC research, these cell lines are used in concert with organoids (which retain patient-specific architecture and heterogeneity) and in vivo models like patient-derived xenografts or genetically engineered mice, to build a more complete preclinical picture. In effect, 2D cell lines have become preliminary models—invaluable for dissecting molecular pathways and performing high-throughput screens—but they are typically complemented by 3D and in vivo studies before clinical translation. Despite their shortcomings, the enduring use of 2D CRC lines attests to their robustness and experimental convenience. They allow extensive replicates, genetic manipulations (siRNA/CRISPR knockouts can be readily carried out, as compiled in Table 5 for targeted genes), and long-term observation that would be challenging in more complex systems. By combining multiple cell lines, researchers can partially simulate tumor heterogeneity (each line representing a different tumor subtype) and ensure that discovered phenomena are not isolated to a single genetic background. In conclusion, these 2D CRC cell line models—HT29, HCT116, SW480, LoVo, DLD-1, Caco-2, and LS174T—provide a foundational platform for CRC research, offering a balance of clinical relevance, molecular diversity, and experimental tractability. When used judiciously alongside advanced models, they greatly accelerate our understanding of CRC biology and the development of effective therapies.

### 2.2. Three-Dimensional (3D) Spheroid Models in Colorectal Cancer Research

**Figure 4:**
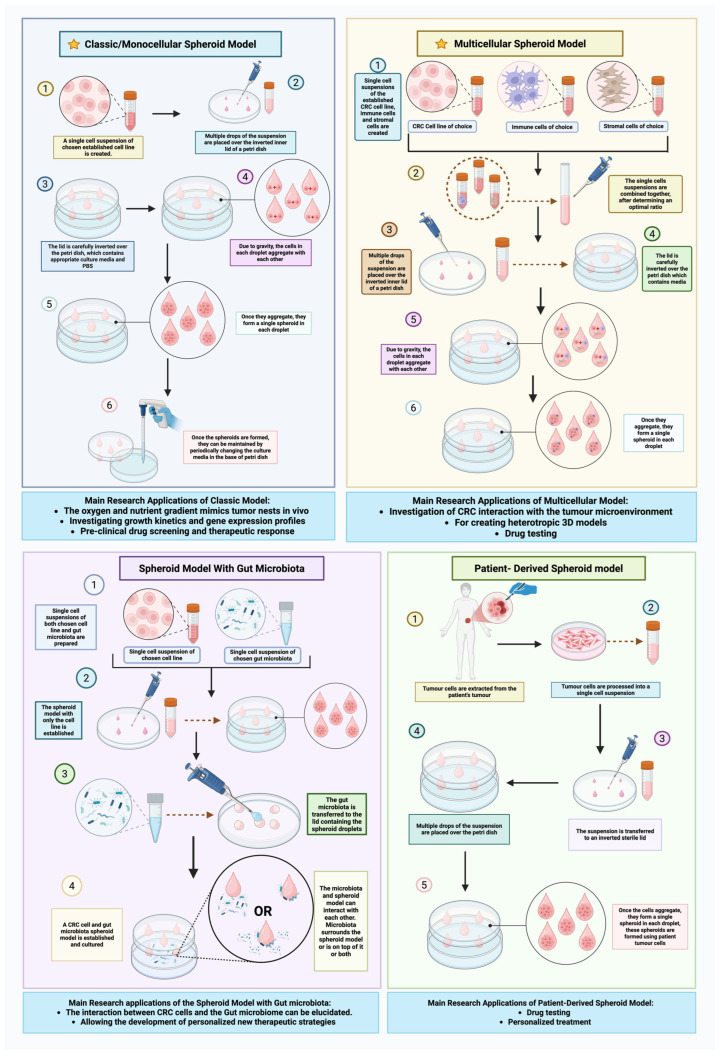
**Protocols and Functional Uses of Colorectal Cancer Spheroid Models.** This figure shows the culture procedures for four different kinds of 3D CRC spheroid models: classic spheroids, multicellular tumor spheroids, gut microbiota-integrated spheroids, and patient-derived spheroids. In addition to highlighting their application in simulating the tumor microenvironment, researching host–microbiota interactions, assessing drug responses, and customizing patient-specific therapy, the figure also demonstrates how culture complexity rises with model accuracy.

3D spheroid models culture colorectal cancer cells as spherical microtumors, providing an in vitro architecture closer to solid tumors than flat monolayers [58]. Unlike 2D cultures, spheroids allow cancer cells to grow in all dimensions and form cell–cell contacts, gradients of oxygen and nutrients, and even central necrosis [59]. Classic multicellular spheroids often develop an outer proliferating layer and inner quiescent core; the limited diffusion in larger spheroids creates hypoxic, nutrient-deprived zones, mimicking conditions of tumor nests in vivo [60]. These structural and microenvironmental features make spheroids more physiologically relevant: for example, CRC cells grown in 3D show markedly different growth kinetics, gene expression profiles, and drug responses compared to 2D monolayers, aligning more closely with patient tumor samples in epigenetic patterns (DNA methylation, microRNA expression) [61]. By preserving a three-dimensional tumor architecture and some aspects of the tumor niche, spheroids represent a critical bridge between traditional cell lines and in vivo models [59]. The protocols and functional uses of these 3D spheroid systems—including patient-derived, multicellular, and microbiota-integrated variants—are summarized in Figure 4.

***Applications and strengths***: 3D CRC spheroids are widely used for preclinical drug screening and therapeutic evaluation. Studies demonstrate that spheroids often reproduce clinically relevant drug responses that 2D cultures miss [62]. For instance, CRC spheroids typically exhibit greater chemoresistance than 2D cultures—an effect attributed to their multi-layered structure and microenvironment [61]. Drugs like 5-fluorouracil, oxaliplatin, and doxorubicin show reduced efficacy in CRC spheroid models relative to 2D, reflecting the diminished penetration and the influence of hypoxia on drug response [63]. Importantly, spheroids can be co-cultured with stromal or immune cells to create heterotypic 3D models that incorporate the TME. An increasing number of CRC spheroid systems include CAFs, endothelial cells, and immune cells (e.g., macrophages, T cells) to mirror the complex TME signaling in colorectal tumors [59]. The presence of these non-tumor components profoundly influences tumor behavior—diverse stromal and immune cells in 3D spheroids secrete cytokines and growth factors that can activate survival pathways in the cancer cells, modulating proliferation, invasion, and drug resistance [64]. For example, co-culturing CRC spheroids with CAFs leads to abundant fibronectin deposition in the spheroid matrix, coinciding with significantly reduced doxorubicin potency [65]. Similarly, adding tumor-associated macrophages has been shown to induce chemotherapy tolerance through paracrine signaling, and spheroids with gut microbiota components (such as Fusobacterium or other bacteria) reveal how microbial metabolites can drive cancer progression and drug resistance [66,67]. These multicellular spheroids provide a powerful translational platform for testing novel treatments: studies report that 3D CRC spheroids (both monoculture and co-culture) often recapitulate in vivo drug response phenotypes [59], supporting their use in high-throughput screens for chemotherapeutics, targeted agents, and nanomedicines. In short, spheroids better capture tumor heterogeneity and pathophysiology than 2D models—enabling insights into tumor–stroma interactions, penetration of drugs through tumor tissue, and the survival of therapy-resistant cell subpopulations (including stem-like, quiescent cells in hypoxic cores) [56].

***Mechanistic insights and illustrative examples***: The tumor-like architecture of spheroids gives rise to phenomena that illuminate CRC biology. As noted, oxygen and nutrient gradients form across spheroid layers [56]. Cells in the spheroid interior experience oxygen deprivation and accumulate metabolic waste, leading to a hypoxic, acidic microenvironment similar to that in poorly perfused tumor regions [68]. Mechanistically, the outer cell layers of the spheroid act as a physical barrier that impedes drug penetration and nutrient diffusion, creating inner zones of low pH and O_2_ that stabilize hypoxia-inducible factors (HIFs) in the cancer cells [69]. This hypoxic core upregulates survival pathways and confers resistance to chemo-radiotherapy, while the acidic milieu can reduce uptake of certain drugs—thereby explaining why spheroids often survive treatments that kill 2D-cultured cells [70]. These microenvironment-induced adaptations mirror in vivo tumor behavior and help identify mechanisms of therapy resistance. For example, spheroids of HCT116 CRC cells develop a core of CD44^high^ slow-cycling cells (a stem-like, therapy-tolerant fraction) under chronic hypoxia [71], recapitulating how cancer stem-cell niches in tumors evade chemotherapy. Co-culture spheroids provide additional insight: a study showed that CRC spheroids grown with macrophages exhibited upregulated PD-L1 expression and an immunosuppressive gene signature [72], suggesting how macrophage-rich tumors might resist immune checkpoint therapy (a hypothesis now testable in vitro with these models). Multicellular spheroids have also been used to study tumor invasion and metastasis; for instance, CRC spheroids embedded in 3D collagen gels invade the matrix with collective cell migration, allowing dissection of pathways driving local invasion [73]. In one recent report, patient-derived CRC spheroids containing CAFs and endothelial cells were implanted in mice and shown to vascularize and invade surrounding tissue, validating that the spheroid’s invasive behavior in vitro reflected true metastatic potential [74]. Such examples underscore that 3D spheroids can faithfully model complex cancer processes—from clonal competition (as drug-resistant clones emerge in the spheroid core) to microenvironmental suppression of immune attack—which are difficult to capture in traditional cultures.

***Limitations and technical challenges***: Despite their advantages, CRC spheroid models face important limitations. Most spheroids are avascular, relying on passive diffusion for nutrients and drug delivery [56]. While this creates useful gradients, it also means they lack the dynamic blood flow and shear forces present in real tumors. The absence of functional vasculature and lymphatics can perturb cell functions—e.g., prolonged interior hypoxia may oversensitize spheroids to HIF-targeted drugs or cause non-physiological cell death that wouldn’t occur in a perfused tumor [71]. Another challenge is standardization and heterogeneity; spheroids often form with varying sizes and shapes, even when derived from the same cell line, which introduces variability in experimental readouts [75]. Different 3D culture methods (ultra-low attachment plates, hanging drops, spinner culture, etc.) yield spheroids that can differ in diameter, cell density and necrotic fraction, complicating comparisons [76]. This lack of uniformity can strongly influence drug response measurements—for example, a 500 µm spheroid may have a large necrotic core and survive a drug that completely penetrates and kills cells in a 200 µm spheroid. Additionally, assessing treatment effects in multi-cell-type spheroids is technically difficult: when a CAF/tumor/immune cell spheroid shrinks or changes viability after treatment, it is challenging to deconvolute which cell population was affected without sophisticated imaging or flow cytometry [59]. Most routine assays (e.g., ATP or acid phosphatase viability assays) measure the spheroid as a whole, so they cannot easily distinguish whether a drug directly killed cancer cells or merely affected supporting cells. A related issue is the lack of standardized endpoints for drug responses in spheroids [77]. Unlike 2D cell viability (which often uses IC_50 values on cell lines), 3D spheroid drug assays vary—some measure spheroid size reduction, others cell proliferation or metabolic activity, and there is no universal “response” criterion for spheroids in the literature [78]. This inconsistency makes it harder to compare results across studies [79]. Finally, although 3D spheroids can include select microenvironmental components, they do not fully recapitulate the systemic environment of a tumor. Key players like the vasculature (for immune cell trafficking and drug pharmacokinetics), neuroendocrine factors, and multi-organ interactions (e.g., liver metabolism of drugs) are absent. Spheroids also tend to be short-term models; they are usually grown for days to weeks and are not passaged long-term (cell lines can be re-plated anew to form spheroids, but the spheroid itself isn’t a self-renewing tissue). Thus, they may not capture long-term clonal evolution or genetic instability as a tumor does over months or years.

***Addressing the limitations***: Researchers are actively developing innovations to improve 3D spheroid models. One approach to compensate for the lack of vasculature is integrating spheroids into microfluidic “organ-on-a-chip” platforms. By embedding CRC spheroids in microfluidic devices with perfused channels, one can supply nutrients and drugs under flow, creating stable concentration gradients and even introducing shear stress akin to blood flow [80,81]. Such spheroid-on-chip systems have demonstrated more uniform drug delivery and can incorporate endothelial-lined channels to mimic rudimentary vasculature, partially alleviating diffusion limitations. In one design, a single CRC spheroid was embedded in a collagen matrix inside a microfluidic chamber; continuous perfusion of media prevented extreme hypoxia and allowed immune cells (added to the flow) to infiltrate the spheroid, modeling a pseudo-vascularized tumor [82]. Parallel advances in bioreactors and 3D printing aim to generate uniformly sized spheroids and even vascularized spheroid constructs. Microwell arrays and hanging-drop systems now produce spheroids of highly reproducible size, which improves the consistency of drug response data [83]. To dissect multi-cellular effects, researchers employ fluorescent tagging or single-cell analysis within spheroids—for example, using distinct reporters for tumor vs. stromal cells, or optical sectioning (confocal microscopy) to visualize cell-specific apoptosis inside treated spheroids. These technologies help determine which cell types are targeted by a therapy [70]. Furthermore, spheroid models are increasingly combined with immune components to approximate immunotherapy scenarios. Co-cultures with cytotoxic T cells or natural killer (NK) cells have been used to measure immune cell infiltration and killing of CRC spheroids [84]. In one study, NK cell addition led to spheroid shrinkage correlating with NK infiltration rate, measurable by a novel flow-based assay [75]. Such setups can evaluate the efficacy of immunotherapies (e.g., bispecific antibodies that recruit T cells to tumor spheroids). Another frontier is incorporating the colorectal microbiome: for instance, spheroids grown in the presence of *Fusobacterium nucleatum* (an oncogenic bacterium in CRC) exhibit increased chemoresistance and invasive behavior, suggesting a path to study microbe–tumor interactions in vitro [85]. By broadening the cellular and biophysical context of spheroids—through microfluidics, co-culture, and controlled engineering—scientists aim to overcome the current shortcomings and make spheroid assays even more predictive of clinical outcomes.

However, despite the sophisticated engineering behind these hybrid spheroid-chip systems, their broader translational impact remains constrained by a series of unresolved regulatory and standardization challenges. OoC technologies, by virtue of their hybrid identity—incorporating aspects of microfluidics, living tissue constructs, and biosensing—fall outside the scope of conventional regulatory definitions applied to either in vitro diagnostics or medical devices. Regulatory bodies such as the FDA and EMA have yet to establish harmonized, dedicated approval pathways, leading to case-by-case adjudication and ambiguity in clinical validation pipelines. Furthermore, the lack of standardized protocols for device fabrication, cell sourcing, perfusion conditions, and endpoint measurements severely limits inter-laboratory reproducibility, which is a cornerstone of regulatory acceptance. The predictive validity of OoC models—particularly in replicating ADME parameters, immune–tumor interactions, and dynamic stromal remodeling—remains under scrutiny, largely due to a paucity of comparative longitudinal human data. Ethical complexities arise from the reliance on primary human tissues and iPSC-derived organoids, necessitating stringent donor consent, biospecimen traceability, and compliance with trans-jurisdictional biobanking laws. As many next-generation spheroid-chip platforms incorporate real-time imaging, multi-omics profiling, and AI-driven analytics, they also face regulatory gaps in algorithmic transparency and digital health governance. Compounding these challenges are intellectual property restrictions stemming from proprietary chip designs and closed-source analytical platforms, which hinder open validation. Moreover, the scalability of OoC systems for use in GLP- or GMP-compliant drug development is hampered by the complexity of manufacturing microfluidic devices with batch consistency and functional stability. Consequently, while OoC innovations continue to expand the biological fidelity of in vitro models, their integration into formal regulatory frameworks for drug screening or therapeutic decision-making will require concerted efforts in standard setting, performance benchmarking, and cross-disciplinary validation consortia.

***Comparative insights***: Compared to traditional 2D cell lines, 3D spheroids clearly offer a more in vivo-like tumor phenotype, restoring features (morphology, gene expression, drug resistance mechanisms) that 2D cultures lose [86] (Table 1). Spheroids thus fill a critical gap between monolayer assays and animal models. When contrasted with patient-derived organoids (PDO) (see below), spheroids have distinct strengths and weaknesses. Cell line-derived spheroids are relatively easy to generate in large numbers and highly reproducible, making them suitable for high-throughput screens. They are advantageous for probing specific cell–cell or cell–matrix interactions by mixing defined cell types (for example, adding a single stromal cell type to gauge its effect). However, spheroids made from established cancer cell lines lack the genetic and clonal heterogeneity of real tumors—each spheroid is genetically uniform (all cells being clones of the parental line) aside from spontaneous variations. PDO, by contrast, intrinsically carry the heterogeneity of the patient’s tumor and often retain multiple subclones. This means PDOs can model patient-specific drug responses and tumor evolution, whereas spheroids from a cell line cannot capture inter-patient genomic diversity. On the other hand, organoids (in their standard form) are usually composed only of epithelial tumor cells and require exogenous extracellular matrix support (Matrigel or similar) for growth. They lack the native stromal cells and immune infiltrates unless these are deliberately added, so they do not automatically recapitulate tumor–stromal crosstalk. Spheroids, especially heterotypic spheroids, are superior for modeling such multicellular interactions in vitro, as they can include fibroblasts, immune cells, and even microbial components by co-culture [59]. In summary, spheroids and organoids offer complementary insights: 3D spheroids (often cell line-based) provide a controlled, tunable model to study how specific microenvironment factors influence CRC cell behavior and drug response [87], whereas PDOs provide a patient-tailored model that preserves the intrinsic tumor makeup (mutational landscape, subclone architecture, differentiation state) for personalized therapy testing. An integrated approach—for instance, adding immune cells or fibroblasts to patient-derived organoid cultures, or validating spheroid findings in PDOs—is increasingly being pursued to exploit the strengths of each model.

### 2.3. Patient-Derived Organoids (PDOs) in Colorectal Cancer Research

**Figure 5:**
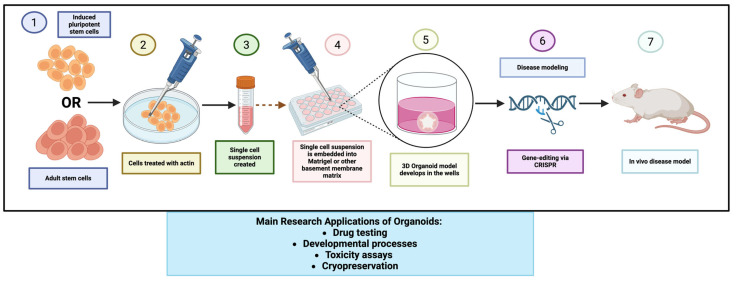
**Culturing Approach and Research Functions of Colorectal Cancer Organoid Models.** This figure outlines the process for culturing CRC organoids from patient tissue, including isolation, embedding in matrix, and expansion phases. It details their use in drug and toxicity screening, modeling developmental processes, and cryopreservation for long-term research. Organoids provide a potent model for translational CRC research and tailored treatment because they closely resemble the architecture of in vivo tissue.

PDOs are three-dimensional microtissues grown from a patient’s own colorectal tumor cells, typically by embedding minced tumor biopsies or isolated cancer stem cells in a laminin-rich gel (e.g., Matrigel) and culturing them with niche factors that support intestinal epithelial growth [88]. Under these conditions, the tumor cells self-organize into 3D structures that recapitulate key features of the original tumor’s histology and cellular composition [89]. Furthermore, the potential of PDOs for predicting sensitivity to combination therapy and the advancements in creating more complex, in vivo-like models are active areas of research [90]. CRC organoids often form hollow gland-like spheres or irregular clusters that mirror the adenocarcinoma architecture—for example, organoids derived from mucinous CRC will produce glandular structures with secreted mucin, and those from poorly differentiated tumors may form solid aggregates. Crucially, xPDO cultures maintain the genetic profile and intratumoral heterogeneity of the source tumor to a remarkable degree [91]. Studies show that CRC organoids retain the vast majority of driving mutations and even the relative clonal frequencies present in the patient’s tumor, across multiple passages. In contrast to cell lines (which undergo clonal selection on plastic) or patient-derived xenografts (which can acquire mouse-specific changes [92], organoids experience minimal genomic drift in early passage and thus represent a preclinical avatar of the individual patient’s cancer. Each organoid line contains self-renewing cancer stem cells that enable it to be expanded for months, as well as more differentiated progeny that collectively reproduce aspects of the tumor’s organization and function [93]. This self-renewal and multi-lineage differentiation capacity means organoids can be propagated long-term (biobanked) while preserving a stem-like compartment and the diversity of cell states found in the tumor (e.g., proliferative zones and quiescent cell pockets), something that conventional 2D cultures cannot achieve. Given these qualities, PDO technology has rapidly matured in recent years and is heralded as a transformative model in CRC research [94], the culturing process and functional uses of CRC organoid models are summarized in Figure 5.

***Applications in Colorectal Cancer Research***: The advent of CRC organoids has opened new avenues in both basic and translational research. One of the most impactful applications is in precision oncology and drug development. PDOs serve as individualized in vitro testing platforms to predict patient therapy responses. A substantial body of evidence now shows that drug sensitivities observed in a patient’s organoids often correlate with that patient’s clinical outcome [87]. For example, researchers have established living organoid biobanks from metastatic CRC patients and performed drug screens to identify effective and ineffective chemotherapies on a case-by-case basis [95]. In one prospective study, CRC PDO drug testing correctly identified over 80% of the patients who would not respond to standard irinotecan-based chemotherapy, thereby flagging those individuals who could avoid the toxicity of an ineffective regimen. Notably, that study also highlighted a current limitation: the same organoid tests did not predict responses to oxaliplatin-based chemotherapy, indicating that further optimization is needed for certain drugs [96]. More generally, PDO platforms have been explored across the continuum of CRC care—from neoadjuvant therapy (predicting which locally advanced rectal cancers will respond to chemoradiation before surgery) to adjuvant chemotherapy selection (testing which post-surgery chemo is most effective for an individual’s tumor) to metastatic treatment (personalizing later-line regimens and targeted therapies) [97,98]. Indeed, recent reviews catalogue the use of CRC organoids in virtually all therapeutic contexts: investigators have used PDO assays to guide postoperative chemotherapy choice, to evaluate targeted inhibitors (e.g., anti-EGFR, BRAF inhibitors) based on the organoid’s mutational profile, to test combinations for chemo-refractory cases, and even to assess treatment options for elderly or frail patients who cannot be trialed on multiple regimens [94]. In clinical research, organoid-based trials are emerging—for instance, drug sensitivity data from a patient’s organoids can be used to make real-time clinical decisions, a paradigm that has shown success in early studies by reducing unnecessary toxicity and improving response rates [99]. Beyond therapy prediction, CRC organoids are a versatile scientific tool for exploring tumor biology. They have been used to study tumor evolution and clonal dynamics, such as how minor subclones present in a primary tumor respond to specific drug pressure (organoids can be sequenced before and after drug exposure to observe selection of resistant clones, modeling the emergence of resistance in vivo) [100]. They also enable investigation of genotype–phenotype relationships in CRC: for example, isogenic organoid lines have been engineered (via CRISPR/Cas9) to introduce or correct mutations (APC, KRAS, p53, etc.), yielding faithful colorectal tumor models that help link certain mutations to drug sensitivities [101]. Furthermore, organoids support studies of TME interactions when co-cultured with other cell types. While pure epithelial organoids lack immune cells or stroma, scientists can merge organoids with immune components to create immuno-organoid models. A recent breakthrough involved co-culturing patient-derived CRC organoids with autologous T cells and other immune cells to simulate the tumor-immune microenvironment for immunotherapy testing [102]. In another study, researchers modeled the response to immune checkpoint inhibitors (ICIs) by combining MSI-high and MSS CRC organoids with their patients’ immune cells, successfully identifying biomarkers of immune resistance and showing that knocking out a certain gene in resistant organoids restored T-cell mediated tumor killing [103]. This exemplifies how PDOs are now driving translational discoveries—in this case, revealing novel targets to overcome immunotherapy resistance. Similarly, organoid co-culture systems with fibroblasts or endothelial cells are used to study tumor invasion and metastasis; for example, placing CRC organoids in a microfluidic “organ-on-chip” device with adjacent stromal channels has allowed observation of cancer cell invasion into a matrix under flow, mimicking metastatic spread and facilitating the testing of anti-invasion drugs [104]. In summary, PDOs have proven applications ranging from personalized drug screening and biomarker discovery to fundamental cancer biology, making them an indispensable model for CRC.

***Strengths and Recent Examples***: PDOs offer several unique strengths that distinguish it from traditional models. First and foremost is the high fidelity in recapitulating patient tumor characteristics. PDOs preserve the histopathological architecture (gland formation, mucin secretion, etc.), the genomic landscape, and the intertumoral heterogeneity of the original CRC tissue [105]. They contain both majority and minority clones from the tumor, including those with stem-like properties that can drive tumor regrowth. This heterogeneity is reflected in drug response: organoids from an individual patient often show a distribution of sensitivities (some organoid sub-clones may survive a treatment that kills others), mirroring the partial responses seen in the clinic. Notably, organoids retain chromosomal instability and mutational spectra characteristic of colorectal cancer—for example, microsatellite-unstable CRCs yield organoids that remain MSI-high and MMR-deficient, useful for testing immunotherapies, whereas chromosomally unstable tumors produce organoids with diverse aneuploidy patterns akin to the tumor [106]. Another strength is the indefinite expansion and biobanking capability of organoids [107]. Once established, a CRC organoid line can be expanded for many passages, frozen, and revived, allowing large numbers of replicate assays and sharing between labs. This has enabled the creation of living biobanks—collections of organoids representing many patients, spanning different molecular subtypes of CRC (e.g., MSI vs. MSS, various CMS subtypes, KRAS/BRAF mutant tumors, etc.). Such biobanks, like the one by van de Wetering et al. with dozens of CRC organoids, have become invaluable resources for drug screening and identifying subtype-specific vulnerabilities [108]. Indeed, screening these organoid libraries has led to novel therapeutic insights (for example, discovering that RSPO-fusion CRCs respond to certain Wnt pathway inhibitors [109]). Not only do PDOs reflect inter-patient heterogeneity, but they also maintain intra-patient diversity to some extent, which is critical for studying clonal evolution under therapy. Researchers have leveraged this to observe how resistance mutations (e.g., emergent RAS mutations upon anti-EGFR therapy) arise in organoids, thereby recapitulating clinically observed resistance mechanisms in a controlled setting. PDOs also recapitulate the drug metabolism and response pathways of tumors more accurately than cell lines. They express relevant drug transporters, activation enzymes, and metabolizing enzymes at levels similar to tumor tissue, which can impact drug efficacy. Additionally, because organoids are grown in 3D with an embedded extracellular matrix (ECM), they experience cell–matrix interactions and mechanical forces that shape cell behavior [110]. The stiffness of the collagen/Matrigel matrix and the 3D context contribute to more physiologic signaling—for instance, organoid cells have apical-basal polarity and form hypoxic cores if they grow large, features that influence drug penetration and cell differentiation. All these factors make organoids highly predictive of clinical outcomes. As one review summarized, organoids have enabled more accurate prediction of patient drug responses and, in cases where organoid-guided treatment was applied, have been associated with improved patient survival through therapy personalization [111]. For example, there are now reports of metastatic CRC patients who, after failing standard care, received a drug predicted to be effective by organoid testing (such as an HER2-targeted regimen for a HER2-amplified organoid) and subsequently experienced tumor regression—anecdotal but powerful evidence of the clinical relevance of organoid findings. Moreover, organoids allow discovery of drug sensitivities in niche contexts: one study found that a subset of CRC organoids carrying a certain ATM mutation were exquisitely sensitive to ATR inhibitors, a relationship that was then confirmed in a clinical trial [112]. Such discoveries underscore PDOs’ role in bridging molecular oncology to tangible therapies.

***Limitations and Challenges***: Notwithstanding their promise, patient-derived organoids come with practical and biological limitations that are the focus of ongoing research. A practical constraint is the need for patient tumor tissue and the associated variability. Establishing organoids requires access to fresh tumor samples (surgical resection or biopsy). While success rates for CRC organoid derivation are relatively high (~70–90% of samples can yield organoids), failure can occur with certain tumor types (e.g., highly necrotic or heavily pretreated tumors may not grow) [95,113]. This reliance on patient tissue means that organoid generation is labor-intensive and depends on clinical coordination. Time is a factor as well: expanding CRC organoids to a sizable population for drug testing typically takes 2–3 weeks, which may be slower than desired for urgent clinical decisions (though still far faster than establishing a PDX, which takes months). Another issue is cost and technical complexity. Organoid culture requires specialized media with multiple growth factors (Wnt, R-spondin, EGF, Noggin, etc.), 3D matrices, and often small-molecule inhibitors to suppress differentiation—all of which make it significantly more expensive than standard cell culture [114]. High-throughput screening with organoids is still developing, as handling 3D Matrigel cultures in multi-well format and performing automated readouts is more complex than for 2D cells. Moreover, there is intra-model variability: organoids from the same tumor can exhibit variability in growth rates or drug responses between batches or labs, due to subtle differences in culture conditions or genetic evolution over long periods [115]. Standardizing organoid assays (e.g., uniform endpoints for drug sensitivity, similar to how IC_50_ is used in cell lines) remains a challenge—as noted in one analysis, there is currently “*no consistent criteria for assessing drug sensitivity tests*” in PDO platforms [116]. Biologically, the lack of a native TME in organoids is a central limitation. Stromal cells (CAFs) are largely absent in routine CRC organoid cultures because the conditions favor epithelial cells and usually involve an initial tissue dissociation step that removes fibroblasts and immune cells. Consequently, PDOs as traditionally grown do not include the tumor’s extracellular matrix (beyond what tumor cells themselves secrete) or stromal signals. This means processes like angiogenesis, immunoediting, and tumor–stroma metabolic crosstalk is not represented. The immune compartment is also missing—tumor-infiltrating lymphocytes, macrophages, etc., are not present unless explicitly co-cultured later. This absence can limit the predictive ability of organoids for treatments that rely on immune involvement (for instance, an organoid may be insensitive to a PD-1 inhibitor simply because it has no T cells with which to interact). The avascular nature of organoids (like spheroids (refer above)) implies that gradients of nutrients and oxygen can form in larger organoids, but without blood vessels, they cannot model drug delivery kinetics or immune cell trafficking in a physiological manner [117]. Organoids also typically grow embedded in artificial matrices like ‘Matrigel’, which, while mimicking basement membrane, may not fully recapitulate the complex ECM of a tumor or its stiffness in vivo. Another limitation is heterogeneity in results and reproducibility. Because each patient’s organoid line is unique, findings in one PDO line need validation across many lines to be generalizable. The flip side of preserving heterogeneity is that organoid responses can be more variable than clonal cell lines—for example, one organoid might respond to a drug while another (with a different mutation profile) does not, making it necessary to test large panels to draw conclusions (which is expensive and time-consuming). There is also potential for clonal drift or selection over extended passaging: if organoids are cultured for very long periods or under drug pressure, certain clones may dominate, possibly altering the representation of the tumor. While genomic studies show organoids remain stable for many passages [118], subtle shifts in clonal composition have been observed (e.g., loss of a subclone with a slight growth disadvantage). Finally, similar to spheroids, measuring complex outcomes in organoids (like invasion or metastasis formation) is not straightforward. Organoids typically remain confined to their gel and do not spontaneously invade into surrounding areas unless specifically modeled in a device; thus, they cannot fully mimic metastatic behavior or multi-organ interactions on their own.

***Strategies to Overcome Limitations***: Researchers are actively refining PDO technology to address these challenges. One major effort is to incorporate microenvironment components into organoid cultures. For stromal inclusion, methods such as co-culturing organoids with patient-derived fibroblasts have shown success [119];—the fibroblasts can surround organoid structures and deposit collagen and other matrix, recreating a mini tumor niche. This organoid-stroma co-culture can restore tumor–CAF paracrine signaling (for example, inducing the organoid to become more invasive or chemo-resistant, as would occur in a CAF-rich tumor). Immune co-culture techniques are also advancing; autologous T cells or peripheral blood lymphocytes can be introduced to organoid cultures to assess immune cell infiltration and tumor cell killing. The development of air–liquid interface (ALI) organoid cultures is another approach [120]: by growing tumor organoids at an interface where immune cells can be added on top, investigators have achieved long-term co-culture of CRC organoids with tumor-infiltrating lymphocytes that maintain cytotoxic function, enabling immunotherapy testing in a pseudo-physiologic context. Additionally, organ-on-chip systems are being combined with organoids to supply mechanical cues and vasculature-like perfusion. In one recent study, CRC organoids were placed in a microfluidic device that applied fluid flow and cyclic strain, simulating peristaltic forces of the intestine, while perfusing nutrients—this led to more robust organoid growth and allowed cancer cells to invade into adjacent stromal compartments on-chip [104]. Such systems can even incorporate endothelial cell-lined microchannels around organoids to mimic blood vessels; endothelial cells form tubular networks that an organoid’s cells can interact with, partially restoring angiogenic signaling. To tackle the issue of standardized drug testing, consortia are working on harmonizing organoid assay protocols. For instance, defining a common readout (like an area-under-curve viability metric after drug exposure) and calibration with reference drugs may allow comparisons across labs. High-throughput adaptations, including automated organoid culture in 384-well plates and high-content imaging, are making large-scale drug screens feasible despite the 3D format. Moreover, new analytic techniques are improving data extraction from organoids: 3D imaging and single-cell RNA sequencing on organoids can reveal which subpopulations survive treatment, giving mechanistic insight into resistance. There is also interest in genetically barcoding organoids—marking different clones with unique DNA barcodes—to quantitatively track clonal evolution within an organoid during treatment. On the clinical side, efforts are underway to reduce the time and cost of organoid-based decision-making: for example, optimizing media to accelerate organoid growth, or using micro-organoids (very small organoid fragments that can be tested sooner). Another promising strategy is to perform organoid drug tests in parallel with tumor DNA/RNA analysis to get a comprehensive view (functional and genomic) of the tumor, thus refining therapy choices. Finally, hybrid models are being explored: for instance, implanting PDOs into mice to create organoid-derived xenografts (ODXs) can provide an in vivo environment (vasculature, immune system) for the organoid, combining the genetic fidelity of organoids with the systemic context of an animal. While such approaches re-introduce the complexity of animal models, they can validate organoid findings and generate larger tissue samples for pharmacodynamic studies. In summary, through co-culture, bioengineering, and assay standardization, scientists are steadily pushing the boundaries of PDO models to more completely emulate real tumors.

***Comparative Insights***: In the landscape of CRC models, patient-derived organoids are often seen as a game-changer, but it is instructive to compare them with other systems (Table 1). Versus 2D cell lines: Organoids have a clear edge in representing patient tumors—they capture heterogeneity, true differentiation states, and realistic drug responses that cell lines frequently fail to show [121]. For example, a conventional cell line might uniformly respond to a drug, whereas an organoid from the same tumor might reveal that only a subset of cells is sensitive while others are resistant, foreshadowing a partial response in the patient. Organoids also maintain the epigenetic landscape of tumors (such as DNA methylation patterns) better than 2D cultures, which tend to acquire artificial epigenetic changes [122]. However, cell lines are still useful for reductionist experiments and are far easier to manipulate genetically; creating isogenic organoid models is laborious by comparison. Versus 3D spheroids: Both spheroids and organoids are 3D, but there are fundamental differences. Spheroids (especially those from cell lines) provide a controlled model with typically one major cell type (unless co-cultured by design), whereas PDOs inherently include whatever cell types (usually epithelial tumor cells) grow out from the patient tissue. Organoids are thus intrinsically tumor-centric, preserving tumor cell heterogeneity and genetic authenticity, while spheroids are a more flexible platform to study cell–cell interactions by mixing cell types. One key distinction is that organoids require a supportive scaffold (Matrigel or similar), which supplies basement membrane components and stiffness that can induce polarization and differentiation of cells [123]. Spheroids, often grown suspension-style, may lack that defined matrix context (though some spheroid methods include collagen or other scaffolds). The ECM context in organoids means, for instance, that CRC organoids can form polarized epithelial sheets with lumen and exhibit secretory functions (like mucin secretion), reflecting the parent tumor’s histology—something a cell line spheroid might not readily do. On the other hand, spheroids can incorporate non-epithelial elements more readily: one can mix immune cells or fibroblasts into a spheroid at known ratios, but adding such cells into established organoids is less straightforward and often requires specialized protocols. In terms of throughput and scalability, spheroids (especially from immortal cell lines) are more amenable to large-scale screens; dozens of identical spheroids can be generated from a cell line, enabling parallel testing of many conditions with minimal variability. PDOs, being patient-specific, usually necessitate a separate culture for each patient sample, with inherent variability between them. However, organoid biobanks alleviate this by providing multiple organoid lines that can be used in parallel to represent a population. In vivo relevance: PDOs arguably offer the closest representation of in vivo tumor biology among in vitro models—they reflect the tumor’s genotype, phenotype, and even patient-specific drug responses [124]. Indeed, organoids have been called “clinical proxies” that can serve as functional diagnostics for treatment selection. Spheroids, while more TME savvy in vitro, do not capture patient-specific genetics unless they are made from patient cells (in which case they are essentially patient-derived spheroids, a concept overlapping with organoids but usually shorter-term). In practice, many researchers use these models complementarily: a drug might be tested on a panel of cell-line spheroids to understand mechanism and on a panel of patient-derived organoids to evaluate efficacy across real tumor genotypes. Finally, compared to in vivo PDX models, organoids are faster, cheaper, and more ethical (no animals needed), and they can be manipulated in vitro (e.g., for mechanistic studies or high-throughput screens) which is difficult with PDX. However, PDX (or organoid-xenograft hybrids) still provide the full organismal context, including immune interactions if humanized mice are used, so they remain a gold standard for some applications. Going forward, the field is trending toward “multi-modal” models—integrating organoids with immune cells, microbes, microfluidic chips, and even AI-driven analysis—to capture the many facets of colorectal cancer biology. In that ecosystem, PDOs stand out as a central hub for personalized translational research, bridging the gap between bench and bedside by enabling ex vivo trials on a patient’s own cancer. Researchers and clinicians selecting among in vitro CRC models will find that while no single model is perfect, patient-derived organoids offer an unprecedented balance of fidelity and experimental control, making them a keystone for advancing CRC therapy and understanding tumor behavior in the era of precision medicine.

### 2.4. In Vivo Models

#### Murine Models of Colorectal Cancer

**Figure 6:**
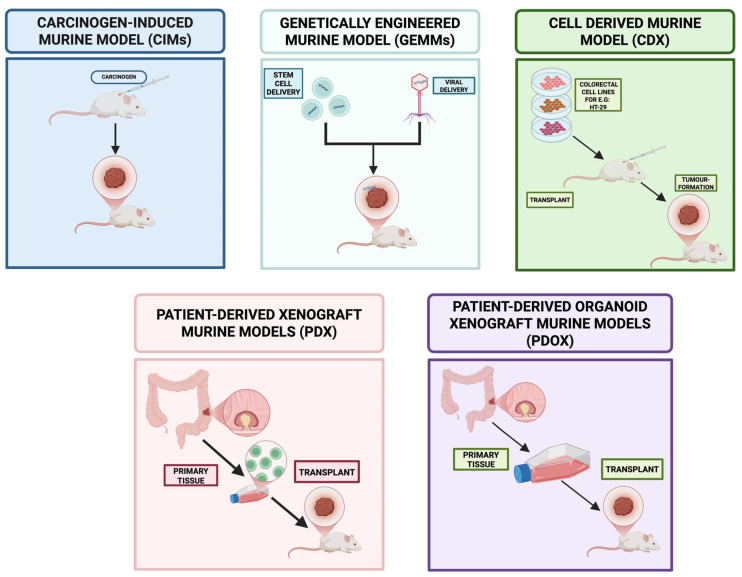
**Comparative Schematics of Established Murine Colorectal Cancer Models.** Methodological pipeline for generating five principal murine CRC models: chemically induced models (CIMs), genetically engineered mouse models (GEMMs), cell line-derived xenografts (CDXs), patient-derived xenografts (PDXs), and patient-derived organoid xenografts (PDOXs).

Murine models have historically constituted the backbone of experimental CRC research, offering versatile and tractable platforms to dissect tumor biology, test therapeutic strategies, and explore tumor–host interactions within an in vivo context. The genetic manipulability, well-characterized immunologic background, and rapid breeding cycles of mice, coupled with the availability of numerous inbred strains, have enabled the development of an array of CRC models that replicate distinct aspects of the human disease. These models (Figure 6), range from chemically induced carcinogenesis systems to genetically engineered mouse models (GEMMs) that mirror human oncogenic mutations, syngeneic transplantable models for immune-oncology studies, and patient-derived xenografts (PDXs) that conserve human tumor heterogeneity. Each category of murine CRC model offers unique advantages while also presenting specific limitations, necessitating strategic selection aligned to the biological question or therapeutic hypothesis under investigation (Table 6). Importantly, recent innovations such as humanized mice and orthotopic implantation techniques have sought to bridge some of the translational gaps inherent in traditional murine models. In the following sections, we critically examine the major types of murine models employed in CRC research, appraising their scientific rationale, methodological nuances, representative applications, and translational relevance, while highlighting emerging strategies to enhance their fidelity to human disease.

### 2.5. Chemically Induced Models (AOM, DSS, and Others)

Chemically induced mouse models use carcinogens and irritants to trigger colorectal tumorigenesis, mimicking sporadic CRC or colitis-associated cancer. A classic example is the azoxymethane (AOM) model, often combined with dextran sulfate sodium (DSS) to induce chronic colitis (Table 6). AOM is a procarcinogen that causes DNA alkylation (e.g., O^6-^methylguanine lesions) leading to point mutations in colon epithelial cells [13]. Repeated AOM injections (typically weekly intraperitoneal doses over several weeks) cause multiple proximal and distal colon tumors within ~3 months [138]. Adding DSS, a colonic irritant, produces cycles of epithelial injury and inflammation, accelerating tumor formation and modeling colitis-associated CRC [139]. The scientific rationale is that these agents replicate environmental and inflammatory insults implicated in human CRC development. Indeed, AOM/DSS tumors exhibit features of inflammation-driven cancer, including elevated NF-κB signaling and cytokine production, analogous to ulcerative colitis-related CRC [140].

*Use Cases*: Chemically induced models are widely used to study early-stage tumorigenesis, chemoprevention, and the inflammation–cancer connection. They allow researchers to examine how repeated injury, and repair can initiate dysplasia and carcinoma in otherwise wild-type mice. For example, recent studies have used AOM/DSS to investigate microbiome influences on tumor formation and to test anti-inflammatory interventions that might prevent colitis-associated cancer [141]. The model’s experimental utility lies in its simplicity and cost-effectiveness: large cohorts of immunocompetent mice can be treated in parallel, and resulting tumors arise in the natural colonic microenvironment with an intact immune system. This enables evaluation of dietary factors, carcinogen dose effects, and preventive compounds in a controlled manner [142].

*Limitations*: AOM-induced tumors are driven by random mutations (commonly activating β-catenin or K-Ras) rather than the orderly APC–KRAS–TP53 sequence of human CRC, so their molecular fidelity to human CRC is limited [143,144]. Tumor heterogeneity and progression can differ from human tumors, case in point, AOM tumors often remain microsatellite-stable and may not acquire the invasive or metastatic properties of advanced human CRC. Likewise, the AOM/DSS model’s heavy inflammatory component can confound studies of interventions (tumor growth may depend on colitis severity, not just oncogenic mutations) [145]. Another limitation is the lengthy protocol and variability: multiple injections over months are required, and DSS-induced colitis can cause inconsistent tumor yield or animal mortality if not carefully titrated [146]. These models also usually lack metastasis—tumors tend to remain in situ in the colon, rarely spreading to liver or other organs, which limits their use for studying the metastatic cascade [147].

*Mitigation Strategies*: Researchers mitigate these limitations by optimizing protocols and combining models. Using moderate DSS cycles reduces mortality while still promoting tumorigenesis, improving reproducibility [148]. To better mirror human mutations, chemical models have been combined with genetically predisposed mice (e.g., treating Apc^Min/+^ mice with AOM to induce more advanced tumors) [149]. Newer approaches deliver carcinogens like AOM directly to the distal colon (via enemas) to focus tumor formation in the colon rather than the small intestine [150]. Additionally, alternative inflammatory agents (e.g., TNBS to simulate Crohn-like Th1 inflammation) have been used in place of DSS to explore different immune microenvironments [151]. Despite their drawbacks, chemically induced CRC models remain a cornerstone for studying environmental carcinogenesis and testing chemopreventive strategies under immunocompetent conditions.

### 2.6. Genetically Engineered Mouse Models (GEMMs)

GEMMs recapitulate specific genetic mutations observed in human CRC, enabling in situ tumor development driven by defined molecular events. The scientific rationale is to mimic the genetic initiation and progression of CRC within an intact mouse organism, capturing the multi-step nature of the disease. A seminal example is the Apc^Min/+^ (Min) mouse, carrying a truncating mutation in the Apc tumor suppressor. Apc^Min/+^ mice spontaneously develop dozens of intestinal adenomas through constitutive Wnt signaling activation, modeling familial adenomatous polyposis (FAP) [127]. However, these polyps occur mostly in the small intestine and rarely progress to invasive carcinoma, highlighting some limitations of early GEMMs [127,152]. Newer models use tissue-specific Cre-lox systems to conditionally delete or mutate Apc (and other genes) in the colon epithelium. For instance, mice with floxed Apc crossed to a colon-specific Cre (e.g., Villin-CreER activated in adulthood) develop colonic adenomas that can progress over time to adenocarcinoma [153]. By engineering combinations of mutations—such as activating KRAS^G12D^ or deleting TP53 alongside Apc loss—researchers have created multi-hit models that better emulate the adenoma–carcinoma sequence and even metastasis [154,155]. These compound GEMMs (often called “triple-mutant” or “AKP” models, for Apc/Kras/P53) can produce invasive colon tumors and occasional liver metastases, mirroring advanced CRC [156].

*Use cases*: GEMMs are invaluable for dissecting the roles of specific pathways (Wnt, EGFR/MAPK, TGF-β, DNA repair, etc.) in CRC initiation and progression. They allow controlled investigation of how a given mutation drives tumorigenesis in vivo and how additional alterations cooperate to influence tumor behavior. For example, Lynch syndrome models with conditional knockout of mismatch repair genes (e.g., Msh2 or Mlh1 under a colon-specific promoter) have been developed to study microsatellite unstable CRC in a native setting [157]. These models develop hypermutant colon tumors that mimic the biology of hereditary MSI-high CRC, providing platforms to test immunotherapies targeting tumors with high neoantigen loads [158]. GEMMs also enable longitudinal studies of tumor evolution, since lesions develop gradually and can be sampled over time or visualized with endoscopy in live mice [159]. Researchers have used GEMMs to evaluate chemoprevention (e.g., NSAIDs in Apc mutant mice) and to test targeted therapies in tumors driven by known oncogenes (such as MEK inhibitors in KRAS-mutant CRC models) [160,161]. The immune system remains intact in GEMMs, so they are also used to study tumor-immune interactions in the context of specific oncogenic events.

*Limitations*: No single GEMM captures the full complexity of human CRC. Many GEMMs, like Apc^Min/+^, show incomplete fidelity to human disease—for instance, Apc^Min/+^ polyps rarely progress to carcinoma and mostly arise in small bowel (whereas human FAP patients mainly have colonic polyps) [127]. Conditional Apc knockout models improve colonic tumor location but often still require additional hits to achieve invasiveness, and even then, the spectrum of mutations in tumors may be limited compared to the heterogeneous mutational profiles of human CRC [162]. GEMMs can be time-consuming and expensive: breeding multiple genetically modified alleles to obtain the desired mutants can take over a year and yield limited cohorts. Additionally, mice often develop tumors synchronously and at young ages, which differs from the typically sporadic, age-related tumor development in humans. Some GEMMs have leaky phenotypes or extra-colonic tumors—for example, germline MMR-knockout mice developed lymphomas or small intestine tumors before colon tumors, confounding their use for CRC specifically [157]. Finally, studying metastasis in GEMMs is challenging while certain models metastasize, the rates are low, requiring large cohorts or promotion strategies to observe sufficient metastatic events [153].

Also, the application of GEMMs in CRC research presents significant ethical and regulatory challenges. The complexity of GEMM generation, involving multi-allelic germline manipulations, necessitates extensive animal breeding, often requiring large cohorts to achieve sufficient statistical power, *which conflicts with the ‘Reduction’ principle of the 3Rs (Replacement, Reduction, and Refinement)*. Furthermore, conditional and inducible gene targeting frequently requires administration of tamoxifen or doxycycline, compounds that themselves may cause off-target effects or stress responses, necessitating ethical justification and rigorous endpoint monitoring. Many GEMMs exhibit early-onset, synchronized tumor development and comorbidities such as anemia, cachexia, or intestinal obstruction, thereby mandating the use of humane endpoints and potentially compromising longitudinal experimental windows. From a genomic engineering perspective, the increasing use of CRISPR/Cas9 in somatic GEMMs introduces its own ethical complexity, as off-target mutations and mosaicism may generate unexpected phenotypes, requiring additional validation cohorts. Regulatory frameworks demand Institutional Animal Care and Use Committee (IACUC) or equivalent approvals for every genetic construct, phenotyping step, and intervention, creating substantial administrative burdens and limiting reproducibility across jurisdictions. Furthermore, the replacement of germline engineering with somatic editing via electroporation or viral vectors—though accelerating model generation—does not obviate ethical obligations; these interventions can cause significant inflammation, pain, and off-target effects, necessitating precise refinement of protocols and analgesic strategies. While humanized GEMMs (e.g., knock-in of human alleles or immune receptors) promise to improve translational fidelity, they also raise ethical questions regarding chimerism and xenogeneic antigen expression. In view of these concerns, ongoing efforts to replace extensive in vivo GEMM use with ex vivo alternatives such as organoid models, or to refine protocols through advanced in vivo imaging that minimizes animal numbers, are imperative. Nonetheless, until such alternatives reach full functional equivalence, the ethical conduct of GEMM-based CRC research must be underpinned by stringent adherence to international animal welfare guidelines, transparent reporting standards (e.g., ARRIVE) [163], and a proactive commitment to ethical refinement in model design and execution.

*Mitigation strategies*: Researchers continually refine GEMMs to enhance their translational relevance. Tissue-specific and inducible gene targeting (using CreER^T2^ systems) confines mutations to the adult colon, avoiding early lethality or off-target cancers and yielding more faithful CRC models. To overcome long latency or lack of progression, multiple genetic alterations are combined (e.g., Apc loss plus Kras mutation plus Tp53 deletion) to drive full malignant progression [156]. The use of viral or electroporation-based somatic gene delivery has emerged as a faster alternative to germline breeding: for instance, in vivo CRISPR/Cas9 plasmids can be introduced to the colonic epithelium to knockout tumor suppressors or knock-in oncogenes, creating autochthonous tumors without generating new mouse lines [164]. Such somatic GEMM approaches greatly accelerate model generation and allow flexible tuning of mutations. Another strategy is humanizing certain genes in mice—for example, replacing the mouse Apc allele with a human APC mutation—to study species-specific drug responses or antigenicity [165]. While GEMMs will never capture all aspects of human CRC, their design improvements and combination with other models (e.g., transplanting GEMM-derived organoids into mice for orthotopic studies) are enhancing their utility as preclinical platforms.

### 2.7. Xenograft Models (Human Tumor Grafts in Mice)

Xenograft models involve transplanting human CRC-derived material into mice, thereby creating chimeric systems where human tumor cells grow in a mouse host. These models are pivotal for preclinical drug testing and personalized oncology because they use actual human cancer cells. Xenografts are typically carried out in immunodeficient mice (such as athymic nude or NOD-SCID strains) to prevent rejection of the human graft [166]. There are two major subtypes of CRC xenografts: ***cell line-derived xenografts (CDX) and patient-derived xenografts (PDX)***.

***Cell Line-Derived Xenografts (CDX)***: CDX models use established human CRCr cell lines grown in culture, which are then injected or implanted into mice. Commonly, millions of cells (from lines such as HCT116, HT-29, SW480, etc.) (refer above in 2D models for details) are injected subcutaneously, forming palpable tumors within a few weeks [167]. This heterotopic (ectopic) approach is straightforward and allows easy monitoring of tumor volume by calipers. CDXs have been a workhorse for evaluating antitumor efficacy of drugs, as they are fast and reproducible; tumors can reach testing size in 2–4 weeks, and cohorts are relatively uniform [168]. These models have been used to screen chemotherapeutic regimens and targeted inhibitors (e.g., EGFR inhibitors tested on EGFR-overexpressing cell line xenografts) [169]. Variations of CDX include orthotopic cell implantation (injecting cells into the cecal/colonic wall or liver to model metastatic lesions) and experimental metastasis models (injecting cells into the spleen or tail vein to seed liver or lung metastases) [170]. The scientific rationale for CDX is to study human tumor biology (growth kinetics, drug response, angiogenesis) in vivo, albeit in a constrained system.

***Patient-Derived Xenografts (PDX)*:** PDX models involve transplanting actual tumor tissue or fragments obtained from CRC patients directly into mice, usually without intermediate cell culture. Typically, a small piece of a surgical resection or biopsy is implanted subcutaneously or orthotopically into an immunodeficient mouse (often called the F0 generation) [171]. If the graft “takes” and grows, it can be expanded into subsequent generations (F1, F2…) by retransplanting tumor pieces into additional mice. PDX tumors retain the histopathological architecture and genetic heterogeneity of the original patient tumor to a greater degree than cell lines [172]. For example, gland formation, stromal content, and subclonal genetic diversity present in the patient’s cancer are often preserved in early-passage PDX tumors. Molecular fidelity is high: PDX models typically carry the same spectrum of driver mutations and gene expression profiles as the donor tumor (including minor subclones), making them a powerful platform for co-clinical trials and biomarker discovery [173]. PDX models have been used to test individualized therapy regimens—in some cases, guiding patient treatment by identifying effective drugs in the mouse avatar (e.g., selecting a responsive chemotherapy based on PDX response) [174,175]. Case in point is a study Wang et al., who developed and characterized a comprehensive panel of PDX models from patients of Chinese Han ethnicity with colorectal cancer liver metastasis (CRLM). These models accurately recapitulated the genetic and chemosensitivity profile of the original patient tumors, thereby allowing the researchers to investigate potential therapeutic targets, including RAS, HER2, and FGFR2. The researchers were able to generate efficacious therapeutic strategies and chemotherapeutic combinations to screen and evaluate for patients lacking existing treatment avenues [176]. In addition, Bertotti et al. established a cohort of “Xenopatients”, which were 85 PDX models derived from patient surgical specimens. These models faithfully retained the genetic and morphological characteristics of the original CRC tumors. This study critically demonstrates the practical utility of PDX models in guiding patient therapy, specifically evidencing how an approach combining anti-ERBB2 and anti-EGFR targeted therapies in PDX models from eight metastatic colorectal cancer patients was used to predict resistance to anti-EGFR monotherapy. A validation trial also evidenced that cetuximab (anti-EGFR therapy) response rates and extent of response were comparable in both xenopatients and patients in clinical settings. Beyond identifying therapeutic approaches, the study also underscored the use of PDX models for prospective stratification of xenopatients into “responders” and “non-responders” based on predictive biomarkers. The stratification based on genetic profile and susceptibility allows for more effective management plans and better prognosis amongst CRC patients [177].

Furthermore, PDX models serve as a valuable platform for comparing the efficacy of diverse CRC therapeutic interventions to identify optimal treatment strategies. Chiron et al. compared the anti-neoplastic effect of two anti-cancer drugs: aflibercept (anti-VEGF) and bevacizumab (anti-VEGF-A). By utilizing PDX models, they demonstrated that aflibercept had increased anticancer functions compared with bevacizumab [178]. Beyond testing potential treatments, PDX models can also be utilized in preclinical trials to identify potential biomarkers, as carried out by Julien et al. Through an experimental cetuximab phase II trial conducted in their 54 PDX models, the researchers validated the key role of KRAS mutation in cetuximab-resistant CRC [179]. By thoroughly characterizing PDX models and correlating them with corresponding patient data, researchers can predict drug responses based on molecular profiles, thereby guiding therapeutic decisions for patients. They are also useful for studying tumors of specific molecular subtypes (such as BRAF-mutant CRC or CMS4 mesenchymal tumors) in vivo, especially when cell lines of those subtypes are scarce [175].

*Advantages and use cases*: Overall, xenografts allow human tumor cells to be studied in vivo, bridging the gap between in vitro studies and clinical trials. CDXs are advantageous for mechanistic studies where a stable, manipulable cell genotype is needed—for instance, testing how a specific oncogene or knockout (engineered in the cell line) affects tumor growth in mice [180]. PDX models excel in translational research: they are considered one of the most predictive preclinical models for drug efficacy since they can recapitulate patient-specific drug responses and resistance mechanisms [181]. Both CDX and PDX are widely employed in oncology drug development pipelines. For example, virtually every new cytotoxic or targeted agent in CRC has been tested in CDX models for efficacy and dosing optimization [182]. Similarly, large PDX repositories have been created to represent CRC diversity and have been screened for sensitivity to novel compounds, yielding insight into genotype-response correlations (such as microsatellite instability correlating with immune checkpoint inhibitor response in humanized PDX mice) [183].

*Limitations*: Despite their utility, xenografts have notable limitations. Because they require immunosuppressed mice, the tumor grows without an adaptive immune context—an important caveat for immunotherapy studies [181]. The lack of T cells and other immune interactions in standard CDX/PDX means these models cannot predict immune-mediated therapeutic effects or immune evasion mechanisms. Additionally, in CDX models, the implanted cell lines are often far removed from primary tumors: they have adapted to 2D culture, may carry culture-induced genetic changes, and typically represent late-stage cancer (since cell lines are usually derived from metastases). Thus, CDX tumors may not accurately reflect a treatment-naïve patient tumor’s behavior or heterogeneity [184]. Microenvironment differences are another issue: in xenografts, the stromal cells (fibroblasts, vasculature, etc.) are predominantly of murine origin. Human tumor cells in a mouse stromal milieu might respond differently to growth factors or drugs than they would in a fully human microenvironment [185]. Moreover, subcutaneous CDX/PDX models are ectopic, lacking the organ-specific interactions of colonic tissue; as a result, they rarely metastasize and may have abnormal metabolic conditions (e.g., different oxygen or nutrient availability in subcutis vs. colon) [186]. Engraftment bias can occur in PDX models: more aggressive tumor clones that adapt to mouse host will dominate, potentially underrepresenting indolent or human-specific aspects of the tumor. Engraftment is also not 100% successful—some patient tumors (especially well-differentiated or treatment-naïve ones) may fail to grow in mice, leading to under-sampling of certain tumor types in PDX collections [187]. PDX generation is time- and resource-intensive, often taking 2–6 months to establish a growing tumor and requiring specialized facilities. Finally, ethical considerations arise when large numbers of immunocompromised mice are used, and there are concerns about the evolution of human tumor cells in a mouse (e.g., emergence of mouse-specific adaptations, selection of murine pathogen-resistant clones) [188,189].

Moreover, the generation and utilization of PDX models are fraught with complex ethical considerations spanning patient rights, data governance, and animal welfare. Procurement of human tumor specimens necessitates rigorously obtained informed consent, wherein patients must be clearly apprised that their biospecimens will be implanted into immunodeficient animals and may be subjected to extensive molecular analyses, including genomic, transcriptomic, and epigenomic profiling. The ethical validity of such consent is especially contentious when obtained during acute clinical distress or in vulnerable populations such as pediatric patients, where assent and longitudinal re-consent may be necessary. Further, broad or blanket consent models are increasingly scrutinized, particularly when tissue use extends to commercial drug development or when derivative data are deposited into public repositories, raising substantial concerns around patient identifiability and data security. Given the high fidelity of PDX tumors to their parental genomic landscape, the risk of re-identification through high-resolution omics data—even when anonymized—necessitates robust ethical oversight and compliance with GDPR-equivalent data protection frameworks [190]. Additionally, inequities in PDX biobanking are becoming evident, as current repositories disproportionately represent tumors from patients at tertiary centers in high-income countries, thereby limiting the applicability of derived insights across diverse populations and potentially reinforcing racial and socioeconomic biases in therapeutic development. From an animal ethics standpoint, PDX protocols often involve extensive tumor passaging in severely immunocompromised murine hosts (e.g., NSG, NOG mice), which lack functional B, T, and NK cells, and are prone to high morbidity due to tumor burden or opportunistic infections. The ethical justification for such practices must adhere to the 3Rs principles—Replacement, Reduction, and Refinement—mandating not only minimization of animal use but also the adoption of refined endpoints, analgesia, and alternative in vitro avatars where feasible. Furthermore, the interspecies context introduces a unique layer of concern regarding the selection of murine-adapted human tumor subclones, which may diverge phenotypically and molecularly from their clinical counterparts, thus confounding translational relevance. As such, while PDX models remain indispensable for pharmacodynamic and resistance studies, their ethical implementation must be continuously recalibrated in light of evolving bioethical, legal, and societal expectations.

*Mitigation strategies*: Several strategies address these limitations. To incorporate immunity, researchers have developed humanized mouse models (refer below for details), where PDX tumors are engrafted into mice reconstituted with human immune cells (derived from human hematopoietic stem cells or peripheral blood) [191]. These humanized PDX models enable testing of immunotherapies (like checkpoint inhibitors or CAR-T cells) in the context of a human immune system, though they remain technically complex and expensive. For microenvironmental fidelity, orthotopic implantation of PDX (into the mouse cecum or liver) is employed to provoke more natural growth patterns and metastasis to liver, improving clinical relevance [192]. Co-culture and co-implantation approaches are also used: for example, mixing human stromal cells or extracellular matrix scaffolds with tumor cells can better recreate tumor architecture and drug responses [193]. To reduce the cost and time, patient-derived organoid xenografts (PDOX) are a new variation, wherein tumor organoids grown from patient cells in vitro are transplanted into mice. PDOXs can shorten engraftment time and allow some in vitro pre-selection of conditions, while still yielding tumors closely resembling the original CRC [194]. Cryopreservation protocols for PDX tumors help create biobanks and allow reuse of the same tumor in multiple experiments, improving reproducibility and reducing the number of new patient samples needed [195]. In drug development, panels of diverse PDX models are now used to perform mouse clinical trials, where cohorts of PDX-bearing mice are treated analogous to human trial arms, to identify responsive subsets and resistance markers before clinical testing [196]. Such integrative use of xenografts maximizes their translational utility while acknowledging their constraints.

### 2.8. Syngeneic Mouse Models

Syngeneic models (*also known as allograft models*) involve transplanting mouse-derived CRC tumors or cell lines into immunocompetent mice of the same genetic background. In contrast to xenografts, the host recognizes the tumor as “self,” allowing a fully intact immune system to engage with the tumor. The scientific rationale for syngeneic models is to study tumor–immune interactions and immunotherapy in vivo, which xenografts cannot readily address. Several murine CRC cell lines are commonly used for syngeneic transplantation: for example, CT26 (a colon carcinoma line from BALB/c mice) and MC38 (from C57BL/6 mice) are two widely used models [159]. These cell lines are typically injected subcutaneously or into the cecum/colon of mice of the matching inbred strain, where they form tumors that can be measured or observed for metastasis. Syngeneic tumors grow rapidly (often palpable within 1–2 weeks subcutaneously), enabling quick experiments [197]. Because the mouse immune system remains intact, therapies such as immune checkpoint inhibitors (anti-PD-1, anti-CTLA-4), cancer vaccines, adoptive T cell transfer, or other immunomodulators can be evaluated for their efficacy and mechanism [134]. For instance, CT26 and MC38 syngeneic models were instrumental in the preclinical development of PD-1/PD-L1 inhibitors, demonstrating tumor rejection or growth delay in response to these agents in immunocompetent hosts.

*Use cases*: Syngeneic models are primarily used in cancer immunology and immunotherapy studies, but also serve for testing chemotherapies and combination treatments in an immunocompetent setting. They are valuable for investigating how tumors evade immune surveillance, how different immune cell populations (T cells, NK cells, myeloid cells) infiltrate and affect tumor growth, and for profiling tumor immunogenicity (both CT26 and MC38 are known to be highly mutagenized and immunogenic, akin to MSI-high human tumors with many neoantigens) [198]. Researchers also use syngeneic models to study metastatic colon cancer: for example, the MC38 cell line can be injected into the spleen or cecal wall of C57BL/6 mice to model liver metastasis formation in the context of a competent immune system [197]. Another application is testing combination therapies—syngeneic tumors allow evaluation of how traditional treatments (radiation, chemotherapy) can synergize with immunotherapies to induce abscopal effects or enhance antigen release for immune priming [199]. Because many genetically engineered CRC models are on a C57BL/6 background, one can also transplant organoids or tumor pieces from GEMM mice into syngeneic hosts to propagate tumors or study late-stage disease with a defined genotype [135]. This hybrid approach combines the genetic specificity of GEMM with the convenience of transplantation.

*Limitations*: While syngeneic models offer an immune-competent system, they have limited genetic and molecular equivalence to human CRC. The available murine CRC cell lines (CT26, MC38, etc.) were originally induced by chemical carcinogens and often harbor a peculiar set of mutations not perfectly representative of human tumors [200]. For example, CT26 carries an activating Kras mutation and a β-catenin mutation, but is p53-wild-type, which is an unusual combination compared to typical human CRC which often has APC and TP53 mutations [201]. Additionally, syngeneic tumors are grown in inbred mice that lack the genetic diversity of human populations, so immune responses may be somewhat uniform and not capture the variability seen in patients. Tumor heterogeneity is limited: clonal cell lines form the tumors, meaning intra-tumoral diversity is low relative to patient tumors or PDXs [202]. The transplant nature of the model (often injecting a high number of cells) can lead to extremely fast-growing tumors that do not undergo the same evolutionary pressures as a naturally arising tumor, potentially oversimplifying the tumor biology. Another issue is that some syngeneic cell lines are so aggressive that they don’t accurately reflect the more indolent growth of many human CRCs—for instance, CT26 can kill a mouse within a month due to rapid growth, whereas human CRC typically grows for years [192]. Regarding metastasis, syngeneic models can be inconsistent: CT26 will occasionally metastasize from a subcutaneous site to lungs, and MC38 orthotopic implantation can yield liver metastases, but these outcomes are variable and often require surgical implantation or very specific techniques to reliably occur [197]. Finally, working with immunocompetent models means that therapies targeting human-specific molecules (e.g., a human-specific antibody drug) cannot be tested unless a surrogate mouse version is available, which can complicate translational studies [203].

*Mitigation strategies*: Researchers are expanding the repertoire of syngeneic models and refining techniques to address these gaps. New syngeneic lines and transplant models are being developed from genetically engineered mice: for example, tumors from an Apc/Trp53 double-knockout mouse have been propagated as an orthotopic transplant model that better reflects human APC-deficient CRC genetics while still being fully immunogenic [204]. Murine tumor organoids can be derived from various GEMM or carcinogen-induced tumors and then transplanted, providing a platform that combines tumor diversity with transplantation. This has led to organoid-based syngeneic models that recapitulate different molecular subtypes of CRC (e.g., CMS classifications) in immune-competent hosts [205]. To improve metastatic modeling, refined orthotopic implantation methods are used—such as injecting a small number of cells or an organoid suspension into the colonic submucosa under endoscopic guidance—to more closely mimic the development of a primary tumor that then invades and spreads [206]. This approach has increased the consistency of metastasis formation in syngeneic models like MC38, though challenges remain in achieving metastatic rates comparable to human disease. Additionally, researchers use bioluminescent or fluorescent reporters in syngeneic tumors to enable non-invasive tracking of tumor growth and dissemination, which helps in quantifying metastatic burden and timing [207]. When studying immunotherapies, it is common to include multiple syngeneic models (e.g., one “hot” immunogenic tumor and one “cold” less-immunogenic tumor) to ensure findings are not idiosyncratic to one cell line. Using a panel of syngeneic models with different characteristics can partially compensate for the limited diversity of any single model [134]. In summary, syngeneic models are essential for immune-related CRC research, and ongoing improvements aim to align their molecular features more closely with human CRC while harnessing their immunological fidelity.

### 2.9. Orthotopic Implantation Models

Orthotopic CRC models involve implanting tumor cells or tissue into the anatomically correct location in the mouse (the colon or rectum), rather than a heterotopic site like the subcutaneous flank. The key rationale is that tumor growth in the native microenvironment will more faithfully reproduce the interactions and metastatic patterns of human CRC. Orthotopic implantation can be performed with either syngeneic mouse tumors or human xenograft material (CDX or PDX), though syngeneic orthotopic models are especially valuable as they combine correct location with an intact immune system [204]. A typical procedure is to surgically expose the cecum or colon of the mouse and inject a suspension of tumor cells (mixed with Matrigel for consistency) into the colon wall, or to suture a small tumor fragment onto the serosal surface [170]. Alternatively, for rectal models, tumor cells can be injected transanally into the rectal mucosa using a specialized injector. When carried out carefully, an implanted tumor grows at the site, invading the colon layers and often forming an obstructive mass, much like human colorectal tumors [208]. Crucially, orthotopic tumors tend to metastasize along physiologically relevant routes—for example, from the cecum/colon to regional mesenteric lymph nodes and the liver via the portal circulation, which is analogous to human CRC metastasis patterns [209]. This contrasts with subcutaneous models, which rarely metastasize and, if they do, often spread to lungs (via venous circulation) rather than liver.

*Use cases*: Orthotopic models are used to study aspects of tumor biology that depend on the native tissue context. They are the gold standard for investigating metastasis in CRC, including the colon–liver metastatic cascade, tumor invasion of the bowel wall, and interactions with the normal colonic epithelium and microbiota [210]. For example, studies of angiogenesis and hypoxia in CRC have utilized orthotopic tumors to ensure the tumor vasculature and oxygen gradients mimic those in a colon-confined tumor [211]. Researchers also employ orthotopic models to evaluate therapeutic strategies such as surgical resection of primary tumor, adjuvant therapies, and novel locoregional treatments (like intraluminal drug delivery), as the model permits these clinically relevant interventions [212]. In immunocompetent setups, the influence of gut microbiome on tumor growth and immune modulation can be assessed since the tumor is adjacent to the intestinal lumen and microbiota—something not possible in a subcutaneous tumor [213]. Orthotopic PDX models (sometimes called patient-derived orthotopic xenografts, PDOX) are increasingly used for co-clinical trials and drug testing, since they may exhibit higher predictive value for drug responses and metastatic behavior. They allow observation of whether a therapy can shrink the primary colon tumor and also prevent or treat liver metastases, closely mirroring human clinical scenarios [214]. In summary, any study requiring the tumor’s physical presence in the colon (for example, evaluating colonoscopy imaging agents, studying tumor–stroma interactions with the colonic wall, or assessing effects of fecal microbiota) would necessitate an orthotopic model.

*Limitations:* Orthotopic implantation is technically demanding and often less reproducible than simpler models. It typically requires surgery or specialized skills, leading to increased animal stress and post-operative care, and sometimes higher mortality if complications like perforation or peritonitis occur [206]. Tumor take rates can be variable, especially for cell suspensions; some implants may not establish, leading to a fraction of animals without tumors or with very small tumors. This variability means experiments may need larger group sizes or careful verification of tumor growth (e.g., via imaging) before therapeutic studies, complicating logistics. Another limitation is monitoring tumor growth: unlike subcutaneous tumors, orthotopic tumors are internal and not easily measurable with calipers. This necessitates periodic imaging (ultrasound, MRI, or bioluminescence if the tumor cells are labeled) or intermediate sacrifices to assess tumor size and progression [215]. Such imaging requirements add cost and may not have the resolution to detect very early lesions or micrometastases. Additionally, orthotopic tumors might obstruct the bowel or cause animal discomfort at advanced stages, sometimes requiring early endpoints, which can limit the window for therapeutic intervention studies [206]. From a biological standpoint, if human tumor fragments are implanted orthotopically in immunodeficient mice, the environment is colonic but the immune context is still absent—thus each orthotopic model retains the limitations of its underlying source (xenograft or syngeneic). Another consideration is that the surgery itself induces wound-healing responses and inflammation that could affect tumor behavior (for instance, post-surgical inflammation might promote tumor engraftment or alter immune cell trafficking), which needs to be accounted for in experimental design [216].

*Mitigation strategies*: With practice and refined techniques, surgical implantation has become more reliable. Using small-gauge needles and precise microsurgical methods can improve cell engraftment while minimizing injury to the bowel [217,218]. Many protocols now employ bioluminescent tumor cells for real-time tracking: tumor cells transduced with luciferase can be monitored in live mice using an in vivo imaging system (IVIS), allowing researchers to confirm engraftment and quantify tumor burden longitudinally without sacrificing animals [219]. This helps in stratifying mice before randomization (ensuring each treatment group has comparable tumor load) and in observing metastatic spread to the liver or lungs through photon signals. To improve take rates, especially for PDX, tumor fragments can be implanted within supportive matrices or with a small piece of the original patient stroma to help initial vascularization [181]. Some studies also pre-condition the implantation site (e.g., lightly scraping the serosa or using a minute dose of DSS to create a pro-inflammatory niche) to enhance tumor cell engraftment in the colon tissue [170]. Additionally, orthotopic models are often combined with advanced imaging modalities like high-frequency ultrasound or PET/CT using tumor-targeted tracers to non-invasively measure tumor size and metabolic activity, respectively [220]. While these tools add complexity, they yield richer data from orthotopic studies. Finally, the use of orthotopic models is usually reserved for questions where they are truly necessary (such as metastasis or microenvironment studies); for routine drug screening, simpler subcutaneous models are used first, and only promising therapies advance to orthotopic testing. In this way, orthotopic models serve as a critical confirmatory step that can validate findings from easier models in a setting that better simulates human CRC behavior [221].

### 2.10. Non-Murine Models of Colorectal Cancer

Beyond mouse models, a variety of other organisms—from invertebrates to large mammals—have been employed to study colorectal cancer. These non-murine models each offer unique advantages in terms of tractability, physiology, or similarity to human CRC, and they contribute complementary insights. Below, we discuss several such models (zebrafish, Drosophila, canine, porcine, and primate), analyzing their relevance to CRC research, key findings, limitations, and how they can be integrated with other systems.

### 2.11. Zebrafish Models

**Figure 7:**
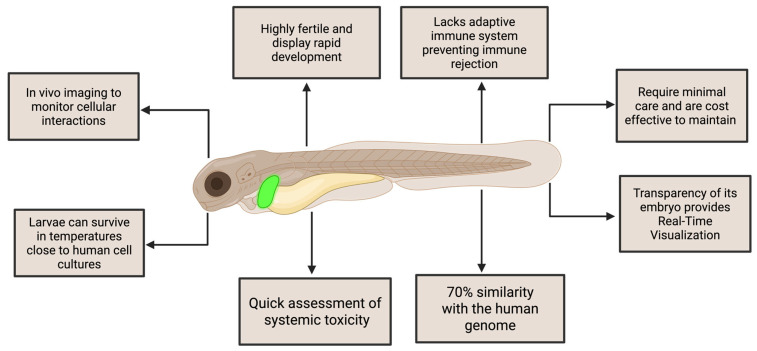
**Unique Advantages of Zebrafish Models for Colorectal Cancer Research.** Key benefits of zebrafish as an experimental platform for CRC studies include: High fecundity enabling large-scale studies, optical transparency permitting real-time tumor visualization, lack of adaptive immune system, minimal cost, and conserved cancer pathways with 70–80% human gene homology [226,227].

Zebrafish (*Danio rerio*) have emerged as a valuable model in cancer research due to their optical transparency as embryos, high fecundity, and genetic manipulability, in addition to having several other advantages as indicted in Figure 7. In CRC research, zebrafish are used in two main ways: genetic models of intestinal neoplasia and transplant models for tumor cell behavior. On the genetic front, zebrafish can be engineered to carry mutations analogous to human CRC drivers. For example, zebrafish with mutations in the APC gene (the ortholog of human APC) develop intestinal hyperplasia and adenomas, recapitulating aspects of Wnt-driven tumor initiation [222]. The histopathology of zebrafish intestinal tumors (architecture of glands, nuclear atypia) is reported to be remarkably similar to human adenomas, demonstrating a degree of morphological and molecular fidelity [223]. These fish models have been used to study the earliest stages of tumor formation in vivo, including how dysregulated Wnt signaling in intestinal stem cells leads to microadenoma formation [224]. Because zebrafish embryos and larvae are transparent, researchers can live-image the intestinal epithelium and visualize processes like cell proliferation, invasion, and angiogenesis in developing tumors at single-cell resolution [225]. This is nearly impossible in mammalian models and is a unique strength of zebrafish.

The second approach is the zebrafish xenograft: human CRC cells are fluorescently labeled and microinjected into young zebrafish (often 2-day-old larvae) [228]. At this early stage, zebrafish lack a mature adaptive immune system, so they tolerate human cell grafts for several days. The cancer cells engraft within the fish (commonly in the perivitelline space or circulation) and can be observed migrating, proliferating, and interacting with vasculature in real time. This platform has been used for high-throughput drug screening—one can introduce dozens of embryos with tumor cells into multi-well plates and expose them to different compounds, observing tumor cell clearance or apoptosis within 5–7 days [229,230]. Key findings from recent zebrafish xenograft studies include elucidation of metastatic behavior (e.g., identifying which CRC subclones are more invasive by watching them intravasate and form micrometastases in the fish) and testing of novel drug combinations in vivo at a scale unattainable in mice [231]. Zebrafish are also amenable to chemical carcinogenesis approaches; exposure of adult fish to carcinogens like dimethylhydrazine or nitrosamines can induce gastrointestinal tumors, though this is less commonly used than genetic models [232].

*Relevance and Integration*: Zebrafish offer a unique bridge between cell culture and mammalian models. They share a considerable portion of their genome with humans and have conserved pathways (Wnt, EGFR, p53, etc.), so drug and genetic findings in fish often translate to mammals [233]. Their small size and aquatic nature make them suitable for medium-throughput screening: researchers have identified potential CRC therapeutics by screening libraries for compounds that suppress the growth of zebrafish intestinal tumors or human CRC cell xenografts in fish [18,230]. Such hits can then be validated in mouse models (refer above), illustrating how zebrafish integrate into a pipeline with murine studies (early discovery in fish, followed by preclinical testing in mice). Zebrafish can also be used to study tumor biology in the context of whole-organism physiology (including interactions with liver, pancreas, etc., which co-develop in the embryo) much faster than mice, since zebrafish reach adulthood in a few months.

*Limitations*: Despite these advantages, zebrafish are aquatic ectotherms, and there are important differences from humans. The body temperature of zebrafish (~28 °C) is significantly lower than human (~37 °C), which imposes constraints: human cells in xenografts proliferate more slowly at fish temperature, and long-term propagation of human tumors in adult fish is not feasible without specialized techniques [234]. The immune system of zebrafish, while having analogous innate and adaptive components, is simpler and not identical to mammals. Adult zebrafish do have T and B cells, but their cytokine networks and immunological memory differ, so immunotherapy studies are not straightforward in this model. Additionally, the scale and anatomy of the zebrafish GI tract is very different—the fish intestine has folds but not true crypts/villi like mammals, and the microbiome composition in zebrafish (an aquatic diet) is distinct [235]. Thus, certain CRC aspects (like polyp formation or fecal stream effects) are not directly replicated. Zebrafish tumors (in genetic models) often require many months to develop, and the fish can succumb to other issues (like oedema or background infections) before tumors become large, complicating aging studies [204]. Another limitation is the throughput of imaging and analysis: while one can generate many embryos, tracking individual tumor cell fates across dozens of fish via microscopy is labor-intensive, and quantification can be challenging. Finally, as an animal model, zebrafish lack the clinical endpoints of human CRC—they do not develop colonic polyps that can be endoscopically monitored or overt liver metastases that mirror human disease burden.

*Mitigation strategies*: Researchers mitigate zebrafish limitations by focusing on what the model does best—in vivo visualization and rapid screening—and not overextending it to questions better suited for mammals. They use temperature-adapted cancer cell lines for xenografts or limit the assay duration to ensure meaningful results before thermal differences confound the data [236]. The development of immunodeficient zebrafish strains (e.g., Rag1-null fish) now allows adult zebrafish to carry human tumor xenografts for longer periods, partially overcoming the short window of larval assays [237]. Further, CRISPR/Cas9 gene editing is routinely used in zebrafish to knock out or modify genes of interest, enabling the creation of multiplexed mutant fish lines (for instance, modeling combined APC and KRAS mutations) much faster than breeding mice [238]. This is a powerful way to study gene interactions in CRC. Combination models are also explored: one can perform initial drug screens in zebrafish embryos, then test top hits in mouse xenografts, and even return to fish for mechanistic questions by creating transgenic fish with reporters for pathways (e.g., a Wnt signaling reporter) to see how drugs act in vivo [231]. In summary, zebrafish models are best utilized in synergy with mammalian models—they provide insight and narrow down hypotheses, which can then be validated in mice or other systems, thus integrating seamlessly into a mult7i-model CRC research strategy.

### 2.12. Drosophila (Fruit Fly) Models

**Figure 8:**
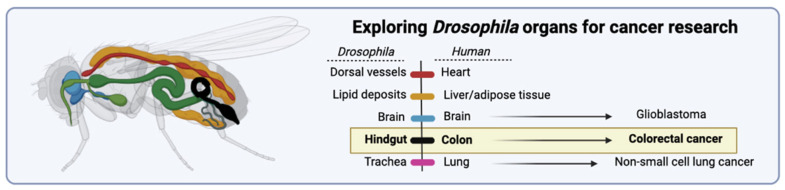
**Drosophila Melanogaster as a Conserved Model for Human Solid Tumors.** Color-coded schematics compare Drosophila organs (brain lobes, gut, trachea) to human tumor histology.

The fruit fly *Drosophila melanogaster* is an invertebrate model that, perhaps surprisingly, contributes to CRC research by allowing sophisticated genetic studies of conserved pathways. Drosophila has a simpler alimentary tract with a midgut analogous to the mammalian small intestine/colon, containing intestinal stem cells that continuously regenerate the epithelium (Figure 8). This regenerative capacity and the conservation of key signaling pathways make the fly gut a useful system to model aspects of epithelial tumorigenesis [239,240]. Researchers have developed Drosophila intestinal tumor models primarily by leveraging the powerful genetic tools available in this organism. For example, targeted knockdown or mutation of the Apc homolog (Adenomatous polyposis coli gene, known as Apc or aprataxin in flies) in gut stem cells leads to hyperproliferation due to Wnt pathway activation, simulating the initiating event of many human CRCs [241]. When combined with overactivation of the RAS/MAPK pathway (e.g., expressing an oncogenic Ras^V12^ in the intestine) or loss of epithelial polarity genes (such as scribble or dlg), flies develop dysplastic lesions in the gut that share hallmarks of neoplastic growth—loss of differentiation, invasive behavior, and recruitment of immune-like blood cells to the area [242,243]. These multi-hit models in Drosophila have been used to dissect the synergy between oncogenic mutations and the microenvironment. For instance, studies have shown that chronic activation of the JNK pathway (mimicking inflammation) cooperates with Ras activation to drive invasive intestinal tumors in flies, highlighting how inflammatory signaling can promote CRC [244].

*Use Cases and key findings*: The main use of Drosophila in CRC research is for genetic screens and pathway analysis. Flies are amenable to large-scale random mutagenesis or RNA interference screens to identify novel genes that modify tumor growth. Using a fly intestinal tumor model, one can knock down each gene in the genome (via RNAi libraries) in the tumor cells and observe if the tumors shrink or grow, thereby discovering new tumor suppressors or oncogenic collaborators [245]. This approach has yielded insights into conserved regulators of Wnt signaling, metabolism, and cell–cell interactions that were later validated in mammalian systems [246]. Moreover, Drosophila allows rapid testing of gene combinations; researchers can simulate the stepwise progression of CRC by sequentially adding mutations in a single fly strain (for example, APC loss, then Ras activation, then p53 homolog loss) to see how each step contributes to malignancy [19]. The timeline for generating and analyzing such complex genotypes is weeks in flies, versus years in mice. Another contribution of Drosophila is the ease of drug testing in vivo on a small scale. While not as high throughput as zebrafish for drugs, flies can be fed compounds by mixing them in food, and effects on gut tumors can be assessed. This has been used to test epigenetic drugs and dietary supplements on Ras-driven fly gut tumors, providing preliminary efficacy data [247]. One striking example of integration is using Drosophila to personalize therapy: “Avatar” fly models have been created by expressing combinations of human oncogenes in the fly gut to mimic a patient’s tumor mutation profile, then treating those flies with panels of drugs to identify effective ones [248,249]. Though a bit unconventional, such studies illustrate the potential of flies to inform treatment strategies that can then be tried in mammalian models or clinics.

*Limitations*: Drosophila is evolutionarily distant from humans, and many aspects of CRC cannot be modeled in the fly. Flies lack an adaptive immune system entirely—no equivalent of T or B cells—so the influence of the immune response on tumor progression is mostly absent (aside from innate immune-like responses by hemocytes, which are much simpler) [250,251]. The tumor microenvironment in a fly is also vastly simpler: flies do not have an organ like a liver to metastasize to, and their body size severely limits any analog of metastasis research. Fly tumors are microscopic and confined to the tiny gut; they can invade locally but cannot recapitulate the full metastatic cascade or clinical features like angiogenesis-driven tumor expansion (flies have an open circulatory system, not a closed vasculature like mammals) [252]. Additionally, certain key genes in human CRC are not conserved in flies. For instance, flies do not have an exact TP53 homolog playing the same role as human p53 in DNA damage response (the fly gene p53 is present but its functions differ and flies tolerate its loss without widespread cancer). Thus, modeling TP53 mutation effects in Drosophila is challenging [253]. Similarly, pathways like TGF-β signaling have only partial conservation [254]. Another limitation is anatomical: the fly “colon” (hindgut) is not a site of active proliferation, most intestinal stem cells reside in the midgut; thus, modeling colonic adenocarcinoma per se is approximated by midgut tumors which are more like small-intestine context [255]. The short lifespan of flies (on the order of weeks) means they cannot model long latency diseases or those requiring years of interactions with diet and environment. They also cannot simulate the effect of aging, which is a significant factor in human CRC incidence.

*Mitigation and integration*: Despite these limitations, Drosophila’s role is to provide rapid insights that can be later confirmed in vertebrates. Researchers mitigate the fly’s simplicity by focusing on highly conserved processes. For example, any potential new drug target found in a fly screen would typically be a fundamental cell growth or signaling regulator, which is likely conserved and relevant to human CRC (any fly-specific hits are filtered out in analysis) [256]. Integration with other models is key: hits from Drosophila genetic screens are often tested next in zebrafish or mouse models to ensure relevance. Conversely, flies can be used to explore hypotheses that arise from human data—for instance, if a large human genomic study finds an association of a certain gene with CRC progression, one can delete that gene in a fly tumor model to quickly see if it modulates tumor growth (a form of in vivo functional validation) [19,257]. Flies have also been used to probe drug side effects on normal stem cells vs. tumor cells: by having parallel flies, one with normal gut and one with a tumor, both exposed to a drug, researchers can observe differential effects on normal tissue homeostasis versus tumor growth, something that foreshadows therapeutic windows [258,259]. To overcome the lack of an adaptive immune system, findings related to immunology in CRC are simply not addressed in flies; those questions are left to mouse models. Instead, flies excel in studying cell-autonomous tumor processes and cell–cell signaling within the epithelium and its immediate microenvironment. By acknowledging what flies cannot do, scientists use them appropriately as part of a complementary toolkit. In summary, Drosophila models, while incapable of capturing the full clinical picture of CRC, are extremely powerful for uncovering fundamental genetic interactions and have successfully guided higher-level CRC research by generating hypotheses quickly and efficiently [260].

### 2.13. Canine Models (Dog)

Domestic dogs (*Canis lupus familiaris*) can serve as a comparative model for CRC, especially given their closer size, physiology, and shared environment with humans. Unlike laboratory-induced models, canine CRC models are typically based on spontaneously occurring tumors in pet dogs, making them a form of naturally arising model of disease. Dogs have a relatively low incidence of CRC (estimated < 1% of dogs develop spontaneous intestinal tumors), but when it occurs, it shows noteworthy parallels to human CRC [261,262]. Certain breeds of dogs may have higher predisposition to intestinal polyps or carcinomas, and the tumors often arise in the distal colon and rectum, like humans [261]. The pathology of canine colorectal tumors includes adenomatous polyps that can progress to adenocarcinomas. Studies have shown that canine colorectal adenomas frequently exhibit dysregulation of the Wnt signaling pathway—for instance, immunohistochemistry in dog polyps shows nuclear β-catenin accumulation, analogous to APC mutation effects in human adenomas [263]. This suggests dogs undergo a similar adenoma-carcinoma sequence. However, an interesting difference is noted: malignant progression in canine colon tumors often occurs without the typical TP53 mutations that accompany human CRC progression [263,264]. This indicates that while the upstream drivers (Wnt pathway, etc.) might be shared, the later genetic events can diverge, or dogs may have alternative mechanisms of tumor suppressor inactivation.

*Use cases and importance*: Canine models are especially valuable in the field of comparative oncology. The National Cancer Institute’s Comparative Oncology Program, for example, has included dogs in clinical trials of new cancer therapies because pet dogs with cancer represent a bridge between mouse models and human patients [265]. For CRC, dogs can help answer questions about long-term tumor evolution, malignancy, and metastasis in an outbred, immunocompetent large animal. They live in the same homes as humans, are exposed to similar environmental risk factors (diet, pollutants), and develop tumors naturally over years, which means canine cancers capture the multifactorial nature of carcinogenesis that lab models might miss [266]. Researchers have reported that canine colorectal cancers can metastasize to the lung, mirroring human disease progression [267]. This makes dogs a potential model for studying metastasis and for evaluating imaging modalities—indeed, veterinarians can perform endoscopies, CT scans, and even experimental PET imaging on dogs to track tumor development and response to therapy, much as would be clinically carried out in humans [23]. One key use of canine CRC cases has been in therapy trials. Case in point for instance, NSAIDs (nonsteroidal anti-inflammatory drugs) and COX-2 inhibitors that showed chemopreventive or therapeutic promise in human CRC have been tested in dogs with colorectal polyps or carcinomas, since dogs also often overexpress COX-2 in their tumors [268,269]. These trials can provide proof-of-concept of efficacy and dosing in a setting where the tumor and host are more similar in size/metabolism to humans than mice are. Additionally, immune-based therapies (like cancer vaccines or checkpoint inhibitors) could theoretically be evaluated in dogs, as dogs have a competent immune system and share many immunological features with humans (dogs even suffer from checkpoint inhibitor-related toxicities in trials, indicating the translational relevance of their immune responses) [270].

*Limitations*: The use of canine models in CRC research is constrained by several factors. Low incidence and availability are a primary limitation—spontaneous colorectal tumors in dogs are uncommon, so assembling a large cohort of dogs for study is challenging [271]. This is unlike canine osteosarcoma or lymphoma, which are more common and hence more often studied in comparative oncology. When cases do occur, they are usually in older dogs, and often at an advanced stage by the time of diagnosis (like humans), which can limit the window for intervention studies. Ethically and practically, one cannot induce CRC in dogs through experimental means (giving a dog a carcinogen would be unethical), so researchers are reliant on naturally occurring cases or perhaps dogs with genetic predispositions, if identified. Genetic manipulability in dogs is essentially absent in a laboratory sense—there are no transgenic dog lines for CRC research, and one cannot easily knock out genes or create inducible mutations in dogs as we do in mice. This means the mechanistic studies in dogs are mostly observational or correlative. Additionally, working with large animals is expensive and resource-intensive: housing, feeding, and medically managing dogs in a research setting costs vastly more than maintaining mice or fish. Each dog requires individualized care and veterinary expertise. The life span of dogs (10–15 years) also means studies can be protracted if looking at outcomes like survival or tumor latency, although their lifespan is shorter than humans which is somewhat advantageous. There are also biological differences, canine gastrointestinal anatomy has some differences (for example, dogs have a shorter colon relative to body length, and their intestinal microbiome differs due to carnivorous diet). These differences could influence how CRC develops or responds to treatment, and results in dogs might not fully translate to humans. Furthermore, the genetic background of pet dogs is heterogeneous (even within a breed) and their prior medical history (some may have received other treatments) can introduce variability that is hard to control, unlike the uniform inbred mice. Lastly, ethical considerations are prominent—pet dogs are often beloved family members, and their participation in research requires owner consent and careful ethical oversight. Studies usually coincide with treatment of the dog’s condition for its own benefit, which means purely experimental endpoints (like tissue collection) are limited to what would be carried out clinically or after natural death/euthanasia for humane reasons [272].

*Mitigation and future directions*: Researchers mitigate these challenges by focusing on observational studies and clinical trials in dogs rather than experimental induction. For instance, veterinary pathologists collect tumor samples from pet dogs with CRC to perform genomic and molecular analyses, comparing them to human CRC datasets to identify shared mutations or pathways [273]. Such studies have found overlaps (like alterations in β-catenin, mismatch repair defects in some cases, etc.) and differences (such as lower TP53 mutation rate) between canine and human CRC, providing insights into which pathways are fundamental to colon carcinogenesis across species [267]. When possible, multi-institutional networks enroll canine patients in trials of new therapies (e.g., testing a novel targeted drug that showed promise in mice). The outcomes—tumor response on imaging, side effects, survival—inform human trials, as dogs can reveal pharmacokinetic and pharmacodynamic information in a large-body context [274]. To integrate canine data with other models, a comparative approach is taken: for example, if a certain drug works in mouse models, giving it to dogs with CRC can validate its efficacy in a more human-like physiology. Conversely, if dogs unexpectedly do not respond or have toxicities, that can caution against moving to human trials. Another area of integration is in understanding metastasis: autopsy studies of dogs that died of CRC can map metastatic patterns and tumor microenvironment features, which can be compared with mouse metastasis models to see if mice recapitulate the same features or if adjustments (like adding certain mutations) might be needed [275]. In terms of future development, identifying or creating canine predisposition models could be valuable—for instance, if a heritable mutation in dogs that causes polyposis or Lynch-like syndrome is discovered, those dogs (in a controlled colony) could become a higher-throughput model for intervention (somewhat akin to how we use GEMMs in mice) [276]. Gene editing in dogs is still in theearly stages, but theoretically could produce a dog with a specific mutation (though ethical use of such technology would be a concern and is not currently practiced for cancer modeling). Another approach could be xenografting canine tumors into mice to create a dog-tumor PDX, which might allow some study of canine tumor biology in the lab without needing the dog present (this has been carried out for some canine cancers) [277]. Overall, canine models provide a poignant reminder that cancer is a cross-species disease and studying it in pet dogs under veterinary care not only helps the animals but also yields comparative insights that enrich our understanding of CRC in humans.

### 2.14. Porcine Models (Pig)

Pigs (*Sus scrofa domestica*) are increasingly recognized for their potential in modeling human diseases due to their close physiological resemblance to humans. In CRC research, pigs offer an intermediate between small rodent models and human patients, especially in terms of organ size, gastrointestinal anatomy, and metabolic similarities. The pig GI tract has a spiral colon with similar dimensions and function to the human colon, and pigs can be trained to undergo procedures like colonoscopies, making them attractive for longitudinal studies of colorectal tumor development [278]. There are two main categories of porcine CRC models: spontaneous/induced tumors and genetically engineered pigs.

*Spontaneous and induced tumors*: Spontaneous CRC is extremely rare in pigs; Notably, pigs share significant physiological and functional similarities with humans in colonic physiology, including water and electrolyte absorption, peristalsis mediated by the colonic muscle layer, and microbial fermentation of undigested material to produce bioactive metabolites. Both species exhibit comparable digestion kinetics for macro- and micronutrients, though differences exist in their gut microbiomes, particularly in butyrate-producing bacteria. Advances in gnotobiotic models have enabled successful colonization of pig intestines with human-derived microbiota, including *Bifidobacterium* and *Bacteroides*, enhancing the translational relevance of porcine models. Consequently, pigs are increasingly utilized to study human gastrointestinal pathologies, such as *Helicobacter pylori*-associated gastritis, gastric ulcers, and cancer predisposition, as well as metabolic disorders, necrotizing enterocolitis, and diet-induced obesity. These shared features support the utility of porcine models in investigating CRC mechanisms, particularly when combined with microbiome modulation or genetic engineering to improve tumor susceptibility [279]. More commonly discussed are models of colonic polyposis in pigs. Notably, researchers have created a pig model of familial adenomatous polyposis (FAP) by introducing a germline mutation in the APC gene. In one such model, pigs carrying an APC truncation (orthologous to a human FAP mutation) developed multiple adenomas throughout the colon and rectum over time [25]. This porcine FAP model demonstrated that, like humans and Apc mutant mice (refer above), Wnt pathway dysregulation drives polyp formation in pigs, validating the pig as a genetically relevant model. However, a key finding was that while pigs developed numerous polyps, these lesions often remained benign adenomas for extended periods and showed slow progression—no invasive carcinoma was observed in the initial report within the pigs’ observation span [280]. This underlines both an advantage (faithful polyp biology) and a limitation (incomplete progression) of the pig model. Pigs have also been used to study colitis-associated carcinoma by using agents like DSS to induce colitis, though sustained models of CAC in pigs are not well-established due to the challenge of chronic treatment in a large animal [281].

*Genetically engineered pig models*: Advances in gene editing (such as CRISPR/Cas9) and somatic transgenesis have enabled the creation of inducible porcine tumor models. One landmark example is the “Oncopig” cancer model, which is a transgenic pig engineered to carry latent oncogenic mutations (such as a KRAS^G12D^ and TP53^R167H^ cassette) that can be activated by Cre recombinase [282]. By delivering Cre (via an adenoviral vector or direct injection) to an organ, one can induce site-specific tumors in the pig. The anatomical similarity between the pig and GI tracts has made porcine models particularly valuable for CRC research, especially in advancing endoscopic technologies. The APC^1311^ pig model has already contributed to the development of fluorescence-guided endoscopy using cathepsin protease-activatable probes [283], fluorescent silica nanoparticle-based detection of adenomas via video-rate fluorescence-assisted white-light endoscopy [284], and the training of artificial intelligence systems to enhance adenoma detection [285]. These approaches improve early identification of colonic dysplasia, a critical step in CRC prevention [286].

Beyond imaging, pigs also serve as valuable translational models for intestinal epithelial stem cell (ISC) research. CRISPR/Cas9 editing has been used to generate an LGR5-H2B-GFP transgenic pig model, enabling the tracking and isolation of ISCs. Organoids derived from this model have been employed to study CRC. Notably, this model expresses OLFM4, along with three other ISC markers found in human intestines. In contrast, mouse models limit OLFM4 expression to the small intestine, making the pig a more appropriate model for studying OLFM4-positive CRC and potential metastasis [287].

Genetically engineered porcine models also offer significant insights into the molecular drivers of CRC. Studies using the APC^1311^ model have reported increased TP53 expression in cases of severe polyposis [288]. More recent work with the TP53^R167H^ pig model demonstrated expression of TP53 isoforms and circular RNAs that more closely resemble those in humans, providing a more accurate platform for studying TP53’s role in colon carcinogenesis [289]. This model has since been expanded to include an inducible KRAS^G12D^ mutation [26], which reflects human pathology where KRAS mutations are found in over 25% of CRC cases [290]. These Cre-inducible KRAS^G12D^ TP53^R167H^ pigs developed reproducible mesenchymal tumors, supporting the model’s relevance.

To further tailor this system to intestinal cancer research, Callesen et al. refined the model by restricting recombination to the intestinal epithelium using a tissue-specific promoter, leading to the development of duodenal carcinoma [291]. However, additional studies are necessary to establish consistent carcinoma development in the lower intestine, which would more closely model human CRC progression and metastasis in a large animal system.

*Advantages and use cases*: Pigs, being large omnivores, have diets and gut microbiomes that can be modulated similarly to humans, so they are useful for nutritional studies and microbiome–CRC interaction research [292,293]. Given the physiological and anatomical similarities between pigs and humans, one could hypothesize that feeding a pig a high-fat, low-fiber diet might promote polyp formation over time in an APC mutant pig, mirroring dietary risk factor studies in human CRC [294]. Importantly, pigs also share a high degree of genome homology with humans, including conservation of key cancer-related genes, which enhances the translational relevance of findings in CRC models [295]. The anatomical size of pigs allows use of standard endoscopic equipment, so serial biopsies and polyp resections can be performed, enabling within-subject study of tumor progression or regression [283]. Pigs also have sufficient blood volume and physiology for repeated blood draws, testing of systemic biomarkers (like CEA levels, circulating tumor DNA), and trialing systemic therapies at human-like doses. Their immune system is closer to humans than rodents in many respects, so immune-based therapies tested in pigs might better predict human responses or toxicities [296]. Additionally, pigs are long-lived relative to rodents; a pig can be studied for several years, which is useful for examining long-term outcomes, late toxicities of interventions, or secondary effects (e.g., metabolic syndrome from a treatment).

*Limitations:* Working with pigs carries substantial challenges. Cost and husbandry are significant barriers—housing pigs requires farm-like facilities or large animal research centers, and each pig consumes considerable feed and space. Therefore, sample sizes in pig studies are inherently small (often just a handful of animals), limiting statistical power [279]. The long time to tumor development in many pig models (e.g., many months to a year for polyps to form in APC mutant pigs) means studies are lengthy and labor-intensive [25]. From a genetic standpoint, creating and propagating genetically modified pigs is slow (a pig’s gestation is ~4 months, and reaching sexual maturity takes 6–8 months). Breeding transgenic pigs or knocking in specific alleles is far less efficient than in mice, and the numbers of animals generated are low. There is also variability in pigs as they are often outbred or only partially inbred, which can lead to variability in tumor phenotype even with the same mutation [297]. Ethical concerns are non-trivial: pigs are intelligent mammals, and their use in invasive cancer experiments must be justified and overseen strictly; endpoints have to minimize suffering, meaning pigs might need to be euthanized when tumors cause discomfort (like obstruction or pain), potentially truncating experiments. On the scientific side, one limitation observed is that pig tumors, especially genetically induced ones, might not evolve the same co-mutations seen in human tumors. For example, an APC-mutant pig adenoma might not spontaneously pick up KRAS or TP53 mutations within the study timeframe, whereas human adenomas in a FAP patient often do over decades. This can result in pig tumors that are more benign than typical human tumors unless multiple hits are engineered in from the start [298]. Finally, the lack of pig-specific reagents (antibodies, molecular probes) can hamper detailed immunological or molecular analysis, though this is improving over time as interest in pig models grows.

*Integration and future prospects*: Porcine models are best viewed as specialized, high-fidelity platforms to be used when mouse models are insufficient. A logical strategy is a tiered approach: hypotheses and therapies are first tested in mice (rapid and cost-effective), and only those that show promise move to a pig model for further validation in a system that mimics human clinical conditions. For instance, a new endoscopic device for tumor ablation could be trialed in an inducible pig CRC model to see if it fully ablates a tumor and how the colon heals, data that would be directly relevant to human endoscopy practice [283]. Similarly, a chemo-radiation protocol might be piloted in a pig model of rectal cancer to gauge normal tissue toxicity in a human-sized pelvis before a clinical trial. The pig’s size also means it can be used for multi-modality imaging: a pig with CRC can be imaged with CT, MRI, and PET, facilitating the development of new imaging tracers or protocols that can later be applied to patients [282]. To enhance the pig model, researchers are exploring creating pig avatars of human patients—for example, implanting a patient’s tumor (xenograft) into a pig. While engraftment of human tissue in a pig is immunologically challenging due to rejection, there have been attempts with immunosuppressive regimens or humanized pigs to allow short-term growth of xenografts for better simulation of the patient tumor in a larger setting [299,300]. Another future direction is to produce miniature pig breeds with cancer predispositions. Mini-pigs or cloned pigs that carry mutations can be easier to handle (30–50 kg instead of 200+ kg) while retaining the biological advantages of the porcine model [301]. If successful, this could reduce costs and improve manageability. Integration with other models also includes cross-species comparisons: the gene expression profiles of pig tumors can be compared with those of human and mouse tumors to identify conserved signatures or pig-specific peculiarities that need to be accounted for [302]. In conclusion, porcine models of CRC, though demanding, offer unparalleled translational relevance. They serve as an in vivo test-bed for procedures and therapies under human-like conditions and help bridge the gap between rodent studies and human clinical trials. As genetic engineering in pigs becomes more routine and costs decrease, we can expect pigs to play a growing role in preclinical CRC research, especially for validating interventions intended for clinical use [286].

### 2.15. Non-Human Primate Models

Non-human primates (NHPs) are our closest relatives in the animal kingdom, and in principle they could serve as highly faithful models for human diseases, including CRC. In practice, NHP use in cancer research is extremely limited due to ethical, logistical, and financial constraints. There is no commonly used experimental NHP model for inducing CRC; rather, what we know about CRC in primates comes from sporadic cases in captive colonies and retrospective analyses. Notably, rhesus macaques (*Macaca mulatta*) have been reported to develop spontaneous colorectal adenomas and adenocarcinomas, particularly in the context of a hereditary predisposition akin to Lynch syndrome (hereditary non-polyposis colorectal cancer) [303]. Over the past few decades, veterinarians at primate research centers observed that a subset of rhesus monkeys were prone to early-onset colon cancer. Investigation revealed that these animals often had defects in DNA mismatch repair (MMR) genes—for example, somatic or germline loss of the MLH1 gene—leading to microsatellite instability and CRC development, very much analogous to Lynch syndrome in humans [304]. This discovery, sometimes referred to as MLH1-deficient rhesus CRC, is significant as it provides a proof-of-concept that NHPs can naturally mirror specific subtypes of human CRC, including the molecular pathogenesis (MMR deficiency, high mutational burden) and pathology (right-sided colon cancers, as seen in Lynch) [305]. Additionally, some NHPs (like certain marmosets or tamarins) are known to develop colitis in captivity and occasionally colitis-associated carcinoma of the colon, offering parallels to human ulcerative colitis progressing to CRC [306]. For example, the cotton-top tamarin, a small primate, famously has a high incidence of chronic colitis and has been documented to develop colonic adenocarcinoma arising from long-standing colitic inflammation [307].

*Use cases and contributions*: Non-human primate data is primarily valuable for confirming that the fundamental biology of CRC holds true in a closest-to-human species. The observed cases of CRC in macaques have shown similar clinical behavior (like metastasis to lymph nodes and liver) and histopathological features (e.g., mucinous adenocarcinomas with signet-ring cells in some instances) as human CRC [308]. These observations lend confidence that mechanisms identified in humans and tested in mice are not an artifact of rodents—if the same patterns appear in monkeys spontaneously, it underscores their validity. In terms of direct research use, there have been a few instances where existing cases of NHP CRC were used to test treatments (for example, administering a chemotherapy regimen to a monkey with colon cancer, akin to a single-animal case study) [309,310]. The results can highlight potential efficacy or toxicity issues in a physiology much closer to humans than a mouse.

Perhaps the most tangible contribution of NHPs is in the realm of immunoprevention and vaccine development. Because monkeys have an immune system extremely similar to humans, any prophylactic cancer vaccine or immunotherapy strategy that is intended for use in humans might be tested in a small cohort of NHPs to ensure it elicits the desired immune response and is safe, before proceeding to human trials. For example, if one were developing a monoclonal antibody against a tumor-associated antigen relevant to CRC, observing immune responses in the NHPs and any autoimmunity would be a prudent preclinical step [311].

*Limitations*: The limitations of using NHPs in CRC research are profound. Ethical considerations are paramount: primates are highly sentient, often endangered or protected species, and experimentation on them is subject to stringent regulations. Intentionally inducing cancer in a monkey (for instance, by giving carcinogens or attempting genetic modification) is generally considered unethical and is practically unheard of in modern research. Thus, scientists are essentially limited to observing naturally occurring disease or performing minimally invasive research that overlaps with veterinary care. Availability is another issue—there are relatively few CRC cases in NHPs; even at large primate centers, the number of spontaneous CRC cases might be in the tens over many years [312]. This scarcity means statistical studies or large cohorts are impossible. The genetic diversity in primates is high (they are not inbred), and each case might be unique. Also, primates have a long lifespan (rhesus macaques can live 25+ years), so any longitudinal study is extremely time-consuming. Cost is very high: housing a single monkey for research can cost thousands of dollars per year, not including specialized care. Experimental manipulation is limited—genetic engineering in NHPs has been carried out for some diseases (like neurological disorders) using CRISPR or viral vectors in very limited instances, but doing so to create a CRC model would be enormously challenging and controversial [313]. Finally, working with NHPs poses safety concerns for humans (zoonotic disease transmission) and requires sophisticated facilities and training.

*Mitigation and integration*: Given these constraints, the role of NHPs is usually to complement findings from other models rather than to be a primary experimental model. For example, if a particular molecular pathway is suspected to be important in CRC based on mouse and human data, and tissues from a monkey CRC case show the same pathway activation (detected via immunohistochemistry or sequencing), that cross-species validation strengthens the overall hypothesis [314]. In some instances, researchers have performed cross-species genomic comparisons: e.g., comparing the mutation profiles of human, mouse, and macaque colon tumors to see what overlaps (perhaps core tumor suppressors like APC and SMAD4 are always hit, whereas others might be species-specific) [304]. This can highlight which drivers are universally critical. To integrate NHP models with other preclinical systems, every opportunity to study afflicted primates is maximized—for example, if a monkey with CRC undergoes surgery, the resected tumor might be shared across labs for pathology, genomics, or cell line establishment. While NHP-derived cell lines or PDX models could theoretically bridge NHP and murine systems for therapy testing, their translational relevance must be carefully evaluated.

Notably, murine models (including PDXs) often fail to fully recapitulate human stromal biology and the complexity of tumor-microenvironment interactions [315]. This limitation is particularly relevant for targets like fibroblast activation protein (FAP). In mice, FAP is expressed by murine—not human—fibroblasts, requiring surrogate antibodies for preclinical studies. In contrast, NHP tumors (e.g., TBM models) mirror human FAP expression patterns more faithfully, with abundant stromal FAP in tumor-adjacent tissue and minimal expression in tumor cell nests, closely resembling human CRC [316,317]. This allows direct testing of human-targeted therapies (e.g., FAP-4-1BBL) without murine surrogates, underscoring the translational advantage of NHPs for stromal-targeted immunotherapies. Thus, while NHP-derived cell lines or xenografts could expand usability, their limitations in preserving species-specific stromal interactions must be weighed against the superior fidelity of intact NHP models for certain targets.

In terms of preventive studies, where ethically acceptable, NHPs might be involved in dietary or behavioral studies—for example, monitoring colon health in colonies of monkeys on different diets (Mediterranean diet vs. Western diet) or examining colonoscopic screening efficacy in detecting lesions in older monkeys [318,319]. These are more observational but can yield insights translatable to human public health.

Looking forward, the relevance of NHPs may increase with the advent of humanized organoid models and ex vivo systems that reduce the need for live primates. However, if there were a scenario where an NHP model is indispensable (for instance, a prophylactic CRC vaccine that must be tried in a system closest to humans), researchers would design the study to align with veterinary care of the animals. Any positive results from monkeys would be a strong impetus for human trials, given the close homology.

In summary, non-human primates are not routine models for CRC, but the instances where they have been involved underscore that they can naturally recapitulate the disease in ways smaller animals sometimes do not (especially for MSI-high tumors). Their greatest value lies in confirming and fine-tuning approaches in a biologic context almost identical to humans. Due to practical limitations, they remain a niche, last-resort model, used only when necessary and typically in small observational studies [320].

### 2.16. Emerging Integrative Platforms

#### 2.16.1. Humanized Mouse Models

**Figure 9:**
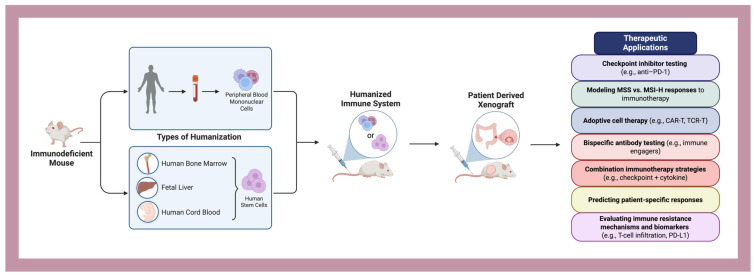
**Schematic Overview of Humanized Mouse Models for Immunotherapy Studies in Colorectal Cancer.** Immunodeficient mice can be humanized by engraftment with either human peripheral blood mononuclear cells (PBMCs) or stem cells, leading to in vivo reconstitution of a functional human immune system. These humanized mice can then be co-engrafted with patient-derived CRC xenografts to model human tumor–immune interactions. This system enables a wide range of immuno-oncology applications, including: (1) testing immune checkpoint inhibitors, (2) modeling differential responses in microsatellite instability-high (MSI-H) versus microsatellite stable (MSS) CRC, (3) evaluating adoptive cell therapies, (4) assessing bispecific antibodies targeting CRC antigens, (5) investigating combination immunotherapies, (6) predicting patient-specific therapeutic responses, and (7) studying mechanisms of immune resistance and tumor adaptation. These models recapitulate key aspects of the human tumor microenvironment and serve as a translational platform for evaluating novel immunotherapies in CRC.

Humanized mouse models are engineered to overcome the absence of an intact human immune system in traditional xenografts. Immunodeficient mice (e.g., NSG strains) are reconstituted with human hematopoietic cells—either CD34^+^ hematopoietic stem cells or peripheral blood mononuclear cells—to generate a functional human immune system in vivo [321,322]. This permits co-engraftment of human colorectal tumors (such as patient-derived xenografts, PDXs) in the presence of human T cells, B cells, and other immune components. The design recapitulates key immune–cancer interactions: human T cells can infiltrate the CRC xenograft and engage tumor antigens, modeling processes like immune surveillance and checkpoint regulation that are absent in immunodeficient mouse hosts. Recent humanized CRC models employ advanced immunodeficient strains (e.g., IL2rγ^null^ mice with knock-in human cytokine genes) to improve human cell engraftment and survival [323]. By partially “humanizing” the tumor microenvironment, these models provide a platform to investigate human-specific immunologic responses to colorectal cancer (Figure 9).

***Experimental utility in Colorectal Cancer (molecular, therapeutic, and immunologic insights)***: Humanized mice enable mechanistic studies of tumor–immune dynamics and therapeutic testing in a context that mirrors human immunology. They are especially invaluable for immuno-oncology research in CRC, where most tumors are microsatellite-stable (MSS) and poorly immunogenic. In humanized models, one can observe human T-cell infiltration, cytokine signaling, and immune checkpoint expression in CRC lesions, offering insight into immune evasion and immunoediting. For example, humanized CRC PDX models have been used to evaluate immune checkpoint blockade: tumors derived from a mismatch-repair deficient (MSI-H) patient showed significant growth inhibition upon anti–PD-1 therapy, whereas MSS tumors in the same models did not—reflecting the differential clinical responses seen in patients [324,325]. This validates the platform’s ability to model therapeutic outcomes that depend on human immune function. Additionally, these models allow testing of adoptive cell therapies (e.g., CAR-T or TCR-T cells targeting CRC antigens) and bispecific immune engagers in vivo, which require human effector cells to mediate tumor killing [191,326]. Molecularly, humanized mice can reveal which tumor-intrinsic pathways are targeted by immune attack or how CRC cells adapt under immune pressure (such as upregulating PD-L1 or other checkpoints) [327]. They also facilitate the study of immunotherapy combinations—for instance, adding cytokine therapies or co-stimulatory agonists—to overcome resistance in “cold” CRC tumors [328]. Thus, humanized models provide a translational bridge for evaluating novel immunotherapies, identifying immune biomarkers, and dissecting tumor–immune crosstalk in colorectal cancer [329].

***Illustrative examples from recent studies***: A 2019 study by Capasso et al. demonstrated the power of humanized PDX models for immunotherapy, where immunodeficient mice reconstituted with human cord blood HSCs were implanted with CRC PDXs and treated with anti–PD-1, resulting in tumor regression in an MSI-H CRC and no response in an MSS CRC, closely mirroring patient outcomes [330]. Notably, this work showed that the human immune system in the mouse can reproduce the well-known immunotherapy sensitivity of MSI-H CRC, something not achievable in conventional mouse models. More recently, Kanikarla-Marie et al. (2022) [191] developed an autologous humanized CRC model by engrafting each mouse with PBMCs from the same patient as the tumor PDX. They found that anti–PD-1 therapy responses in these autologous humanized mice correlated strongly with the patients’ actual clinical responses, whereas allogeneic (unmatched donor) PBMC-engrafted mice showed nonspecific graft-versus-tumor effects unrelated to patient outcome. This highlights that preserving the patient-specific T cell repertoire is critical for faithfully predicting immunotherapy efficacy. Such models have been used to identify which pre-existing tumor-specific T cells drive rejection of CRC lesions and to test combination regimens (e.g., dual checkpoint blockade or checkpoint inhibitor plus vaccine) in a human-relevant setting. Beyond checkpoint inhibitors, humanized mice are being employed to test CAR-T cells against CRC targets (like HER2 variants) and antibody-based immunotherapies, providing evidence of anti-tumor activity and toxicity profiles in the context of human immune cells [191]. Together, these studies illustrate how humanized CRC models are enabling advanced immunotherapy investigations that closely parallel clinical scenarios.

***Technical limitations and current challenges***: Despite their promise, humanized mouse models face significant technical and logistical challenges. Engraftment of a human immune system is inherently complex and costly. Mice reconstituted with human HSCs require specialized strains and often human foetal tissues (liver/thymus) or cord blood, and engraftment can take months. Even then, the reconstituted immune system is incomplete—for instance, human T and NK cells develop, but other components like lymph node architecture or a full repertoire of myeloid cells may be suboptimal [331]. Models using mature PBMC engraftment have a more rapid setup but tend to be short-lived due to xenogeneic graft-versus-host disease (GvHD), where human T cells attack murine tissues. GvHD can severely limit the window for experiments to ~4–5 weeks post-engraftment [332]. Additionally, maintaining these mice is expensive and labor-intensive—specialized housing, precautions to prevent human pathogen transmission, and the need for large quantities of human cells make scalability difficult. There is also considerable variability: differences in HLA matching between donor immune cells and tumor can affect results, and each mouse may reconstitute with different immune cell levels [333]. The stromal microenvironment in these models remains partly murine (e.g., mouse cytokines, fibroblasts, vasculature), which can cross-react differently with human cells and potentially confound certain interactions. Moreover, using patient-derived immune cells and tumors raises ethical and practical issues in obtaining sufficient patient material. Because of these hurdles, humanized CRC models are not yet widespread—many research groups find them unattainable due to high cost and technical expertise required [334,335].

***Strategies to address challenges or integrate with other model systems***: Ongoing innovations are aimed at improving the feasibility and fidelity of humanized models. One strategy is to use autologous immune engraftment (as demonstrated by Kanikarla-Marie et al.) so that the tumor and immune system are HLA-matched, minimizing artifactual immune responses [191]. While patient-matched immune cells are not always available, expanding patient T cells or using induced pluripotent stem cell (iPSC)-derived immune cells specific to that patient’s tumor are potential future approaches. Advances in mouse engineering are also mitigating limitations: next-generation strains express human cytokines (e.g., IL-2, IL-15, GM-CSF) to better support human immune cell development, and knock-in human HLA genes may allow more physiological T-cell education and reduce xenogeneic reactivity [336]. To extend experimental windows, researchers are exploring methods to temper GvHD—for instance, partial HLA matching, T cell subset depletion, or deficient in murine MHC support—though these must be balanced against perturbing the immune-tumor interaction [337]. Integrating humanized models with other systems can enhance translational impact: for example, organoid co-culture systems can be used as a preliminary screen for immunotherapies (e.g., co-culturing organoids with autologous T cells or NK cells in vitro), with the most promising therapies then tested in vivo in humanized mice for confirmation of efficacy and assessment of systemic effects. Cross-disciplinary collaboration is crucial [338]—clinicians can provide tumor/immune specimens and clinical context, while immunologists and mouse modelers refine the systems. There is a push to standardize protocols (e.g., standardized readouts for human immune cell engraftment and function) and to share humanized model resources via consortia so that results are more reproducible and broadly applicable. Ultimately, continued technological advances—including gene-editing, improved human immune engraftment techniques, and possibly in silico modeling to complement in vivo findings—are expected to further empower humanized mouse models as translational tools [339]. These improvements, coupled with careful integration with patient data (such as using patient ‘avatars’ in humanized mice to test therapies), will help bridge the remaining gap between bench and bedside for immunotherapy in colorectal cancer.

To further enhance the fidelity and personalization of humanized CRC models, recent innovations have harnessed the precision of CRISPR-Cas9 gene-editing, enabling targeted modification of both host and tumor compartments.

##### CRISPR-Cas9 Edited Models

The Clustered Regularly Interspaced Short Palindromic Repeats (CRISPR) and CRISPR-associated protein 9 (Cas9) system functions as a programmable molecular scalpel. It consists of the Cas9 nuclease and a guide RNA (gRNA) that directs the nuclease to a specific 20-nucleotide target sequence in the genome. For cleavage to occur, the target site must be adjacent to a short sequence known as the protospacer adjacent motif (PAM). Once guided to the target, Cas9 creates a double-strand break (DSB) in the DNA. The cell’s natural repair machinery then takes over, primarily through one of two pathways: the error-prone non-homologous end joining (NHEJ) pathway, which often results in small insertions or deletions (indels) that disrupt gene function (knockout), or the high-fidelity homology-directed repair (HDR) pathway, which can be used to insert specific genetic sequences (knock-in) if a DNA repair template is provide [340].

***CRISPR-edited organoids:*** To apply CRISPR technology to organoids, the three-dimensional structures must first be dissociated into single cells or small cell clusters using enzymatic reagents such as ACCUTASE™ or TrypLE™. The most commonly employed delivery method for CRISPR components is electroporation, which utilizes electrical pulses to transiently permeabilize the cell membrane. This approach is particularly effective for introducing pre-assembled ribonucleoprotein (RNP) complexes composed of purified Cas9 protein and synthetic guide RNA (gRNA).

RNP delivery is preferred in many experimental settings due to its rapid onset of activity and transient presence within the cell, which reduces the likelihood of off-target effects compared to plasmid- or virus-based methods that result in prolonged expression. Following electroporation, cells are promptly re-plated in Matrigel to support the reformation of organoid structures. To improve cell survival following the mechanical and chemical stress of dissociation and electroporation, the culture medium is typically supplemented with a Rho-associated kinase (ROCK) inhibitor, such as Y-27632 [341].

Once the edited organoids have reformed, individual clones harboring the desired genetic modification can be isolated either through fluorescence-activated cell sorting (FACS) or via manual picking following limiting dilution. These clones are subsequently expanded and verified to establish a stable and genetically defined organoid line [342].

***CRISPR-edited humanized mouse models:*** The integration of CRISPR-Cas9 gene-editing technologies into the generation of humanized mouse models marks a pivotal advancement in translational CRC research. Unlike conventional humanized models that rely on passive engraftment of human cells or tissues, CRISPR enables active genetic reprogramming at multiple levels, enhancing both the immune humanization process and the tumor modeling fidelity.

*Step 1: Host Genome Engineering for Enhanced Human Cell Engraftment:* The process often begins with the use of immunodeficient mouse strains such as NSG (NOD-scid IL2Rγnull) or NOG mice, which are further engineered using CRISPR to improve their compatibility with human hematopoietic stem cells (HSCs) and immune cell subsets. Key murine genes that interfere with human hematopoiesis—such as *Sirpa*, *Il15ra*, or *Mhc class I/II genes*—can be knocked out or humanized using CRISPR [327]. For instance, murine *Sirpa* can be replaced with its human ortholog to enhance macrophage tolerance to human cells, reducing xenograft rejection [343].

*Step 2: Human Cytokine Knock-In to Support Immune Reconstitution:* A major limitation of traditional humanized mice is the poor development of certain immune subsets due to species-specific cytokine incompatibilities. CRISPR-mediated knock-in of human cytokine genes such as *IL-3*, *GM-CSF*, *IL-7*, *M-CSF*, and *TPO* into the mouse genome has been employed to support multilineage hematopoiesis and promote functional development of T cells, B cells, NK cells, and dendritic cells [344]. These knock-ins are often inserted into endogenous loci to ensure physiological expression and reduce ectopic effects.

*Step 3: CRISPR-Edited Patient-Derived Tumor Organoids for Engraftment:* In parallel, patient-derived organoids can be genetically edited using CRISPR-Cas9 to introduce specific mutations commonly found in CRC—such as *APC*, *KRAS*, *TP53*, and *SMAD4*—either singly or in combination [341,345]. These organoids serve as genetically defined tumor inputs for engraftment into the CRISPR-optimized humanized host. This enables the modeling of tumor initiation, progression, and immune escape mechanisms in an in vivo setting that recapitulates the patient’s genomic landscape.

*Step 4: Autologous Immune-Tumor Co-Engraftment*: Advanced models now enable the co-engraftment of CRISPR-edited organoids and autologous immune cells (either PBMCs or CD34^+^ HSCs) derived from the same patient. This approach minimizes alloreactivity and immune rejection, allowing for stable long-term monitoring of tumor-immune interactions [346,347]. In this setup, researchers can evaluate how specific genetic alterations introduced via CRISPR influence immunogenicity, checkpoint ligand expression, or T-cell infiltration within a matched immune context.

*Step 5: Optional Human MHC Engineering:* Some platforms incorporate CRISPR-engineered knock-in of human leukocyte antigen (HLA) class I and II alleles into murine hematopoietic compartments or thymic stromal cells. This promotes more physiologic antigen presentation and T cell education, improving TCR repertoire diversity and functional specificity. CRISPR-engineered knock-in of human HLA class II (e.g., HLA-DRB1*04:01) into thymic epithelial cells enables physiologic MHC II expression, supporting robust T cell education [348]. Moreover, mice co-expressing HLA-A2 and HLA-DR4 demonstrate superior reconstitution of both CD4^+^ and CD8^+^ T cells following human HSC engraftment [349].

***Applications in Colorectal Cancer Research***: The true value of these advanced preclinical models is demonstrated by their application to solve the most pressing and clinically intractable problems in CRC. Recent studies (Table 7) are not merely technical demonstrations, but they represent strategic assaults on major clinical barriers such as therapy resistance, “undruggable” oncogenes, and the failure of immunotherapy in the majority of patients. Furthermore, these applications reveal a blurring of the lines between the model and the therapy, as the core technologies of model creation, CRISPR and stem cells—are increasingly being engineered directly into novel therapeutic modalities.

***Challenges and Mitigation Strategies:*** While CRISPR-edited organoids and humanized mice represent the cutting edge of preclinical research, it is crucial to recognize their inherent limitations. These models exist in a scientific “uncanny valley”, they are remarkably human-like, yet the remaining non-human components and technical artifacts introduce complexities that demand careful experimental design and interpretation. The increasing sophistication of these models places a higher burden on the researcher to dissect whether an observed effect is due to the intended therapeutic mechanism or an artifact of the chimeric system. Furthermore, a fundamental trade-off exists between the high-throughput scalability of organoids and the high-fidelity systemic biology of humanized mice, a dichotomy that defines their respective roles in the research pipeline. For the benefit of the reader, we have identified and summarized some of the key challenges associated with CRISPR-engineered models, along with corresponding mitigation strategies, in the table below (Table 8).

### 2.17. Microfluidic Tumor-on-Chip Systems

**Figure 10:**
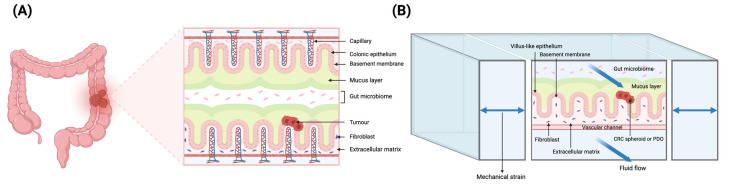
**Modeling Colorectal Cancer Microenvironment: Native Tissue vs. Organ-on-Chip System.** Panel (**A**) illustrates a colorectal cancer (CRC) tumor embedded within the native colonic epithelium, surrounded by mucus, gut microbiota, and a fibroblast-rich extracellular matrix (ECM). This depiction highlights key features of the tumor microenvironment, including stromal components and microbial interactions. Panel (**B**) shows a colon-on-a-chip platform that recapitulates the microarchitecture of the colonic epithelium, including villus-like epithelial structures and a tumor component modeled using spheroids or patient-derived organoids. The tumor sits atop a vascularized ECM layer containing fibroblasts. The system supports physiological features such as mechanical strain, luminal fluid flow, mucus secretion, and co-culture with gut microbiota—collectively enabling a more accurate in vitro mimicry of colonic physiology and CRC pathophysiology.

Microfluidic “tumor-on-a-chip” systems are bioengineered platforms that emulate the tumor microenvironment’s physical and biochemical context with high precision. The rationale behind tumor-on-chip technology is to recapitulate key features of human tumors—3D architecture, multicellular composition, perfused vasculature, and dynamic mechanical forces—in a controlled in vitro device, thereby overcoming the oversimplification of static 2D or even conventional 3D cultures [358]. Figure 10 illustrates the architectural and functional parallels between the native colorectal tumor microenvironment and a colon-on-a-chip platform, highlighting the potential of microfluidic systems to recapitulate complex in vivo features such as vascularization, immune and stromal cell interactions, microbiota presence, and mechanical cues. These chips are typically built using microfabrication techniques to create small chambers and channels through which culture media (and gases or mechanical forces) can be flowed. In a CRC tumor-on-chip, for example, a central chamber might be filled with a 3D collagen or Matrigel matrix containing embedded CRC organoid or spheroid cells, flanked by microfluidic channels that function as blood vessels [359]. Endothelial cells can line these channels to form a microvascular network, and controlled flow through the channels generates shear stress similar to blood flow [360]. This design allows precise manipulation of the tumor’s environment: nutrients and drugs can be perfused in a way that mimics gradient exposure, waste products are removed, and oxygen tension can be modulated to create hypoxic zones, as occur in vivo. The platform also enables incorporation of additional cell types such as fibroblasts, immune cells, or bacteria (to model gut microbiota), compartmentalized in physiologically relevant arrangements. Some advanced CRC-on-chip designs integrate multiple organ components—for instance, connecting a “colon tumor” chamber to a “liver” chamber—to model metastatic spread or pharmacokinetic distribution of drugs across organ systems [361]. Overall, the key design feature is the ability to fine-tune biophysical conditions (flow rates, mechanical stretch, matrix stiffness) and spatiotemporal drug delivery, providing an experimentally tractable yet physiologically relevant model of colorectal tumor growth. Notably, recent efforts have focused on improving chip materials and fabrication: a fully 3D-printed CRC tumor-on-chip was developed with transparent, biocompatible materials to enhance cell viability and allow real-time imaging, while being cost-effective and easily reproducible [362]. Such engineering advances address prior barriers (e.g., PDMS-based chips absorbing drugs or being opaque), ensuring that tumor-on-chip systems meet essential criteria of versatility, optical accessibility, and compatibility with living tissues [363,364].

***Experimental utility in Colorectal Cancer (molecular, therapeutic, and immunologic insights):*** Microfluidic tumor-on-chip models serve as powerful experimental platforms for CRC research, offering insights at multiple levels. Molecularly, they allow researchers to study how the TME’s physical forces and gradients influence cancer cell behavior and gene expression. For instance, a recent CRC organoid-on-chip study showed that applying peristalsis-like cyclic mechanical strain and fluid flow enhanced the invasiveness of KRAS-mutant colon cancer cells, activating mechano-transduction pathways that are quiescent in static organoid culture [104]. Such findings underscore how biomechanical cues (shear stress, compression) in the gut can drive molecular changes (e.g., EMT, MMP secretion) in colorectal tumors—insights difficult to obtain from traditional models. Therapeutically, tumor-on-chip systems provide more predictive drug testing platforms. They enable pharmacokinetic modeling in vitro: drugs can be perfused through the microfluidic channels to mimic dosing regimens and concentration gradients as seen in patients [80]. This is crucial for CRC, where perfusion and drug penetration in dense tumor tissue can alter efficacy. In study by Ong et al., patient-derived CRC spheroids were cultured in a microfluidic device and exposed to various chemotherapy treatments; the chip maintained the microtissues in a viable state over days of perfusion, and drug response profiles on-chip strongly correlated with the patients’ actual clinical outcomes [365]. This exemplifies how tumor-on-chip platforms can be used for personalized medicine: performing ex vivo drug screens on a patient’s tumor under near-physiologic conditions to predict which regimen will work best in the clinic. Additionally, chips are uniquely suited to examine multicellular interactions. For CRC, that includes tumor-stroma crosstalk (e.g., co-culturing cancer cells with cancer-associated fibroblasts under flow to study reciprocal signaling) and immune–tumor interactions. Indeed, organ-on-chip models are being leveraged to probe immunologic aspects of CRC by introducing immune cells into the system. Microfluidic platforms have been designed where circulating immune cells (such as T cells or NK cells) are flowed through a vessel channel adjacent to a tumor chamber; researchers can observe these cells extravasate into the 3D tumor matrix, infiltrate the tumor, and carry out cytotoxic functions [366]. This dynamic modeling of immune cell trafficking and tumor cell killing cannot be captured in static transwell assays. It offers insight into phenomena like immune cell extravasation, activation status upon tumor contact (e.g., loss of CD16 on NK cells after infiltrating the tumor [367], and the efficacy of immune-mediated tumor cell clearance in real time. Metastatic processes can also be studied: by connecting a CRC chip to “downstream” organ chips (liver, lung), investigators have recreated the steps of metastasis—tumor cells invading into circulation, then colonizing distant organ-mimicking chambers. Remarkably, HCT116 colon cancer cells in such a platform preferentially migrated to liver and lung microchambers, mirroring the organotropic metastasis pattern seen in CRC patients [361]. This provides a controlled way to investigate the molecular determinants of metastasis (e.g., adhesion molecules or chemokine receptors guiding CRC cells to the liver) and to test anti-metastatic therapies. Furthermore, tumor-on-chip systems support real-time monitoring—high-resolution microscopy or even on-chip sensors can track tumor growth, cell death, or metabolic changes longitudinally, yielding rich datasets on tumor dynamics and drug responses [368]. In summary, the experimental utility of CRC chips spans from dissecting biomechanical and biochemical influences on tumor biology to performing clinically oriented drug and immunotherapy testing, all within a microphysiologically relevant setting [369,370]. By bridging the gap between simplistic in vitro assays and complex in vivo models, these systems contribute both mechanistic insights and translational data for colorectal cancer.

***Illustrative examples from high-quality recent studies***: Innovative studies continue to push the boundaries of what CRC organ-on-chip models can do. Carvalho et al. (2019) developed a vascularized colorectal tumor-on-a-chip with a central 3D hydrogel laden with CRC cells and parallel perfusable channels, demonstrating its utility for precision nanomedicine delivery—they showed that endothelial networks formed in the chip and that an anti-cancer nanotherapeutic could effectively perfuse and suppress tumor growth in this microenvironment [358]. Steinberg et al. (2023) reported a fully 3D-printed tumor-on-chip platform used to culture patient-derived CRC spheroids; the device’s design (featuring multiple microchambers and easy media access) enabled them to test five chemotherapy regimens on each patient’s tumor spheroids and achieve clinical correlation—the in-chip drug that best reduced spheroid viability corresponded to the regimen that produced the best outcome for that patient in the clinic [362]. This study provides a compelling proof-of-concept for personalized drug screening in colorectal cancer and underscores the potential for chips to guide therapy selection. In the realm of mechanistic discovery, Strelez et al. (2023) [104] combined patient-derived organoids with a gut-mimetic microfluidic platform to examine how physical forces influence tumor invasion. They found that organoid-on-chip cultures subjected to rhythmic compression (mimicking intestinal peristalsis) showed upregulation of invasion-related genes and more aggressive behavior compared to static organoids, particularly in KRAS-mutant tumors [104]. This work revealed a novel facet of CRC biology—that mechanical stimuli in the colonic environment can enhance malignancy—highlighting a potential link between bowel peristalsis and tumor cell dissemination. On the engineering front, researchers have also showcased high-throughput and multi-organ configurations: for example, a “metastasis-on-chip” system was used to model the spread of CRC organoid cells to liver and lung modules, as noted above [361], and Zhang et al. developed a multiplexed microfluidic platform where dozens of tumor migration assays run in parallel, enabling screening of anti-invasion compounds in a throughput-compatible manner [371]. These high-impact studies collectively demonstrate the versatility of CRC tumor-on-chip models—from reproducing patient-specific drug responses and uncovering microenvironment-driven behavior to simulating complex processes like metastasis and immune cell infiltration—firmly establishing these platforms as cutting-edge tools in colorectal cancer research.

***Technical limitations and current challenges***: Notwithstanding their promise, microfluidic tumor-on-chip systems come with practical challenges that must be addressed for broader adoption. One major issue is technical complexity: designing, fabricating, and operating microfluidic devices often requires specialized engineering expertise not typically found in biology labs. The chips themselves can be intricate; ensuring consistent fabrication (especially when created via soft lithography or manual assembly) is non-trivial, leading to variability between devices and between labs. This lack of standardization in chip design (dimensions, materials, flow rates) makes it difficult to compare results across studies and has slowed regulatory acceptance of these models in drug development [372,373]. Material choice is another concern—polydimethylsiloxane (PDMS), a common chip material, is gas-permeable and easy to mold but can absorb hydrophobic drugs and leach uncured oligomers, potentially skewing drug screening results or harming cells. Recent chips using alternative polymers or 3D-printed resins aim to mitigate this, but each new material requires biocompatibility testing [372,374]. Throughput and scalability remain challenging as well. While organ-on-chip devices can be parallelized, they historically handle fewer samples than standard well-plate assays. Efforts are underway to create multi-well chip arrays and automation (e.g., automated microfluidic platforms that interface with liquid handlers) [375], yet running large numbers of chips in parallel (for population-scale screens or replicates) can be resource-intensive. Another limitation is integration of analytical readouts: extracting cells or molecules from chips for downstream assays (genomic, proteomic analyses) can be difficult due to low volumes and the need to disassemble devices. On-chip detection methods (sensors, imaging) help, but these can complicate device design or require sophisticated instrumentation [376]. Biological completeness is also a concern—despite their ability to include multiple components, chips still simplify the system. For example, a CRC-on-chip might lack the full immune repertoire or systemic endocrine signals that affect a tumor in a living organism. Recreating features like the gut microbiome or nervous system innervation in a chip is an ongoing challenge (though some studies have begun to incorporate microbial flows or neuronal cells) [377]. Maintaining long-term cultures on chips poses another hurdle: tumors can be grown for days to a few weeks, but modeling processes like prolonged drug resistance development or tumor evolution would require stable long-term culture without contamination or device degradation [378]. Moreover, user error (e.g., bubbles in microfluidic channels, clogs, or pump malfunctions) can cause experimental failures, indicating the need for robust, user-friendly designs. Data interpretation and validation present challenges too—when a chip yields a complex result (say, a certain flow condition induces a gene expression change), linking that to in vivo relevance requires careful validation in orthogonal models or patient samples [379]. Thus, while tumor-on-chip systems are powerful, they currently demand substantial optimization and expertise, and their throughput and standardization lag behind more traditional assays. Addressing these limitations is critical to move this technology from specialized labs into mainstream use for CRC research and drug development.

***Strategies to address these challenges or integrate with other model systems***: The field is actively working on solutions to make tumor-on-chip platforms more reliable, scalable, and integrative. Standardization initiatives are underway to establish common materials and readout protocols—for example, using 3D printing to produce identical chip units at scale, as demonstrated by Steinberg et al., ensures uniformity and could allow commercial distribution of ready-to-use chips [362]. Likewise, microfluidic consortiums are defining standards for flow rates and media formulations for specific organ chips (akin to how organoid media have standardized recipes) to reduce inter-study variability. To tackle throughput, researchers are developing automated, high-throughput organ-on-chip platforms. One such approach is integrating microfluidic chambers into a format compatible with multi-well plates and standard robotic handlers, enabling simultaneous perfusion of dozens of mini-tumors [380]. Automation can also improve reproducibility by minimizing human error in handling microfluids. Another strategy is coupling organ-on-chip systems with real-time sensors (for pH, oxygen, drug concentration) and machine vision, so that data collection is continuous and less labor-intensive [381]. This also generates big data that can be mined by AI for patterns (e.g., correlating certain dynamic behaviors with drug response). Importantly, combining tumor chips with other model systems can capitalize on their respective strengths. For instance, chips can be used in conjunction with patient-derived organoids (PDOs)—an emerging paradigm is to first expand a patient’s tumor as organoids (to have sufficient material) and then seed the organoids into a microfluidic chip to test drug responses under flow or 3D co-culture conditions [382]. This leverages the scalability of organoid culture and the physiological relevance of the chip. Similarly, organ-on-chip models can incorporate components from in vivo models; one could envision using biopsies or fresh slices from a CRC patient tumor on a chip to maintain native architecture and then perfuse human immune cells through the device, effectively creating a miniaturized, ex vivo human tumor/immune ecosystem for short-term experiments. In the immunotherapy realm, “immune-on-a-chip” designs are being refined—for example, Marzagalli et al. developed a multi-organ-chip for NK cell migration and tumor cell killing, which can be adapted to CRC to test checkpoint inhibitors or cellular immunotherapies in a human fluidic context [367]. These setups could be integrated with humanized mouse findings: e.g., immune cells from a humanized mouse or patient could be tested on a chip against tumor organoids to screen combinations, and promising combos then fed back into mouse studies or clinical trials. Feedback loops between models (chip ↔ organoid, chip ↔ in vivo, chip ↔ in silico) are likely to enhance predictive power. Additionally, computational integration is a key strategy—computational fluid dynamics can be used to design chips that accurately simulate in vivo flow patterns, and PK/PD modeling can interpret chip-based drug response in terms of human dosing [383]. Modelers are even coupling multi-organ chips with computer models to simulate whole-body pharmacokinetics, linking gut, liver, and tumor chips to predict systemic drug behavior. From a practical standpoint, increasing the user-friendliness of tumor-on-chip is crucial: newer platforms come pre-assembled and interface with simple pneumatic pump systems or even passive pumping, lowering the barrier for biological researchers. In terms of validation, efforts are focused on demonstrating that chip models can prospectively predict clinical outcomes (as in the Steinberg study) and recapitulate known biology (for credibility). Regulatory agencies have begun to recognize organ-on-chip data in toxicology, and a similar path is envisioned for efficacy testing once sufficient validation is achieved [384]. In the interim, tumor-on-chip data can augment traditional models—for example, if a drug shows modest effect in a cell line but strong effect in a vascularized CRC chip, it flags a mechanism (like improved delivery or microenvironment modulation) that might be clinically relevant, thus informing trial design. In summary, by standardizing designs, scaling throughput, integrating with organoids and in vivo models, and employing computational tools, microfluidic tumor-on-chip systems are poised to become indispensable components of the preclinical CRC model arsenal, ultimately tightening the bench-to-bedside continuum for cancer therapeutics.

### 2.18. AI-Augmented Computational Frameworks

**Figure 11:**
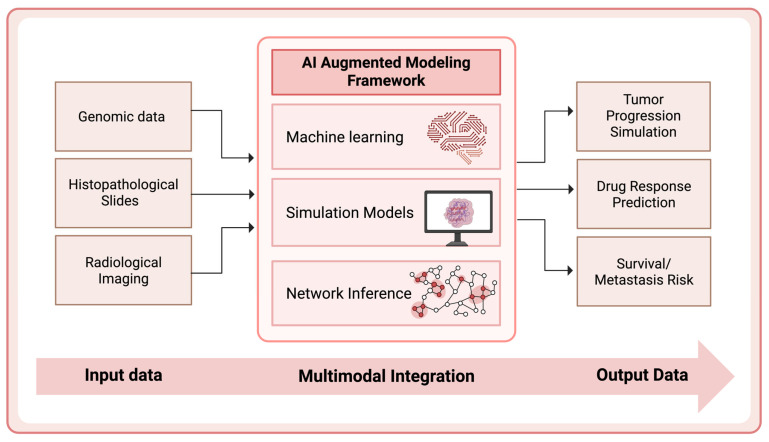
**AI-Augmented Integrative Framework for Predicting Cancer Outcomes.** This figure depicts an AI-augmented modeling framework that integrates multimodal data—including genomic profiles, histopathological images, and radiological scans—to enhance the prediction of cancer outcomes. Input data are processed through a combination of machine learning algorithms, mechanistic simulations, and network-based inference. The framework outputs predictive insights such as tumor progression trajectories, drug response profiles, and survival or metastasis risk assessments. This integrative approach supports comprehensive and personalized oncology by informing prognosis and optimizing therapeutic strategies.

***Scientific rationale and design features:*** Artificial intelligence (AI) and computational modeling frameworks have emerged as transformative tools to simulate and analyze colorectal cancer beyond what purely experimental models can achieve. The rationale for incorporating AI is grounded in CRC’s complexity: tumors exhibit high genetic heterogeneity, nonlinear signaling networks, and varied treatment responses, generating massive datasets (from multi-omics to histopathology to clinical records) that exceed human analytical capacity. This integration is illustrated in Figure 11, which outlines a conceptual AI-augmented framework that combines diverse data modalities and computational approaches to model tumor progression, predict therapy responses, and inform personalized oncology. Recent reviews highlight how AI is increasingly being integrated into cancer research to decode cellular heterogeneity, tumor–microenvironment interactions, and multiomics data, ultimately supporting biomarker discovery, patient stratification, and therapeutic decision-making in translational oncology [385]. AI—encompassing machine learning (ML) and deep learning (DL) techniques—can identify hidden patterns and multivariate relationships within these datasets [386]. For instance, recent studies have applied AI-based segmentation frameworks to quantify residual tumor burden accurately in resection specimens following neoadjuvant therapy, as demonstrated by the ISGPP-2 study in pancreatic cancer [387]. These approaches highlight the potential for AI to enhance translational research in CRC. Modern AI-augmented frameworks in CRC research often combine data-driven algorithms with domain knowledge. For example, a framework may take as input genomic profiles (mutations, gene expression), histological features, and prior knowledge of pathways, and output predictions about tumor behavior or therapy response. Under the hood, this might involve training ML models on large cohorts of CRC cases where outcomes (drug sensitivity, survival, metastasis occurrence) are known, thereby “learning” the complex correlations between input features and outcomes. Some frameworks deploy neural networks to simulate aspects of tumor progression; others use approaches like support vector machines or ensemble methods for predictive modeling. Simulation of CRC progression is a key design goal: computational models can be constructed to virtually “grow” a tumor, incorporating rules of cell division, mutation acquisition, and interaction with a simulated microenvironment. These can be agent-based models or differential equation models augmented with AI to estimate parameters or to introduce adaptive behaviors. Additionally, AI frameworks are being designed to model mutation-based pathway rewiring—that is, predicting how specific mutations (e.g., KRAS^G12D^ or TP53 loss) alter cellular signaling networks and phenotypes [388]. By training on perturbation data or using network inference, the AI can suggest which downstream pathways become hyperactive or redundant when a given mutation is present. The output might be testable predictions like “KRAS mutation confers addiction to the ERK pathway but also induces a backup MEK-independent route,” pointing to combination therapy targets. Another important feature is drug response prediction: Emerging strategies now integrate ex vivo efficacy testing, such as drug response assays in patient-derived organoids, with mathematical modeling frameworks like second-order regression and adaptive lasso to identify synergistic, low-dose drug combinations—offering a more personalized and mechanistically informed approach to CRC therapy design [389]. AI models ingest high-dimensional data (genomic, transcriptomic, proteomic) from tumor samples or cell line panels along with drug sensitivity data to learn predictive signatures for chemotherapy, targeted agents, or novel compounds. These models often employ cross-validation and independent validation to ensure generalizability, and they can be designed to continuously improve as more data (e.g., from organoid drug screens or clinical trials) are fed in. Many frameworks are now multimodal, integrating radiologic images (CT/MRI), pathology slides, and molecular data to build a more comprehensive predictive model—a design well-suited to CRC, where imaging (like identifying lymph node metastases or tumor bulk) and molecular biomarkers (like MSI status) together guide therapy. In sum, AI-augmented frameworks are effectively in silico laboratories for CRC, where hypotheses about tumor growth, metastasis, and treatment can be generated quickly. They serve as a complementary approach to wet-lab models, capable of both guiding experiments (by prioritizing conditions to test) and synthesizing results (finding cohesive interpretations among disparate data). The NIH-style technical design often involves layered networks or ensembles of algorithms that handle various data types and are trained on large, curated CRC datasets, with performance metrics (accuracy, AUROC, etc.) to evaluate their predictive power [390,391]. Crucially, these frameworks are increasingly incorporating explainability (to satisfy biomedical interpretability needs) and are built to be updated with new data, reflecting an iterative learning design well-suited for the evolving landscape of colorectal cancer research.

***Experimental utility in Colorectal Cancer (molecular, therapeutic, and immunologic insights)***: AI-driven computational models have already proven useful in extracting biologically and clinically relevant insights in CRC. On the molecular level, they can decipher complex gene expression patterns and mutational constellations. For instance, unsupervised machine learning on CRC transcriptomes can reveal novel molecular subtypes or gene networks that correlate with aggressiveness, beyond the established consensus molecular subtypes. More mechanistically, AI tools can analyze signaling network data to find points of pathway convergence or compensation—e.g., identifying that EGFR pathway activation in CRC is rewired via alternate receptors when RAS is mutated, which helps explain the failure of EGFR inhibitors and highlights potential co-targeting nodes (perhaps SHP2 or ERK) [392]. In terms of therapeutic insights, one of the most immediate utilities of AI is in predicting drug sensitivity and resistance. This has been demonstrated in recent studies where ML models were trained on data from panels of CRC patient-derived organoids and matched clinical outcomes. Kong et al., for example, integrated genomic and pharmacologic profiles of colorectal and bladder cancer organoids using support vector regression and other ML algorithms to predict patients’ chemotherapy responses. Their model successfully stratified 114 CRC patients treated with 5-FU into likely responders vs. non-responders, with the predicted responders showing significantly longer survival in reality [393]. Such predictions provide actionable insight—i.e., an AI framework can flag which patients are unlikely to benefit from standard chemo, prompting consideration of alternative treatments. Similarly, Park et al. used a random-forest ML model on ex vivo organoid radiotherapy experiments to predict rectal cancer patients’ response to chemoradiation, achieving over 81% accuracy in identifying responders [394]. This kind of tool could guide neoadjuvant treatment decisions (who can safely skip surgery, for instance). AI frameworks are also adept at uncovering biomarkers of response or resistance. They can sift through thousands of features to find those most predictive of outcome—often highlighting genes or proteins not previously obvious. In the organoid-based 5-FU study, the model effectively identified biomarkers associated with drug efficacy, helping improve therapeutic decision-making [393]. On the immunologic front, AI and computational models contribute to understanding and predicting immune responses in CRC. Although most CRCs are MSS and unresponsive to checkpoint inhibitors, AI can help parse why some microsatellite-stable tumors do respond or how to induce responsiveness. For example, deep learning models analyzing the tumor microenvironment (via digitized histology or transcriptomic data) can quantify immune infiltration patterns and correlate them with outcomes. Indeed, emerging AI tools can predict microsatellite instability status from H&E slides [395,396,397], which has direct therapeutic relevance since MSI-high status predicts immunotherapy benefit. Additionally, AI models can integrate tumor genomic data with T-cell receptor sequencing and cytokine profiles to forecast the efficacy of immunotherapies or identify patients who might benefit from novel immunomodulators. There is an active area of research using AI to simulate tumor-immune dynamics—for example, mathematical models of cancer-immune interplay can be calibrated to patient data, then used to test how a tumor’s clonal evolution might proceed under immune pressure or how adding an immunotherapy could shift the balance [398,399,400]. While these simulations are complex, they yield insights such as which immune cell types are most crucial in containing CRC or which immune evasion mechanism (PD-L1 upregulation, antigen loss, etc.) is most likely at play in a given tumor. AI has also been applied to study the role of the microbiome in CRC immunotherapy by analyzing metagenomic profiles and outcomes, finding patterns that human analysis alone could not (e.g., specific bacterial signatures that correlate with immunotherapy success or failure) [401]. In summary, AI-augmented frameworks provide a virtual testing ground for CRC hypotheses—they can rapidly evaluate how combinations of mutations might influence drug response, suggest new drug targets (by identifying network “choke points”), and predict clinical outcomes (like metastasis risk or survival) with a level of accuracy that is steadily improving. Importantly, these computational insights often dovetail with experimental data: for instance, an AI might predict that tumors with high chromosomal instability (CIN) are sensitive to spindle poisons, a prediction that should then be confirmed by in vivo models or clinical trials [402]. In doing so, AI models help prioritize experiments and sharpen our molecular understanding, thereby accelerating the translational research cycle for colorectal cancer.

***Illustrative examples from recent studies***: Several high-quality studies illustrate the impact of AI in CRC research. One landmark example is the development of a digital twin approach for chemotherapy selection: in the study by Kong et al. mentioned above, the authors created a computational model trained on organoid drug testing data, which was then applied to actual patients. It correctly predicted which metastatic CRC patients would benefit from 5-FU-based chemotherapy, with responders identified by the model showing markedly improved survival compared to model-predicted non-responders [393]. This demonstrates AI’s potential to inform treatment decisions. In another study, investigators used machine learning to integrate gene expression data from CRC tumors with their known treatment outcomes and discovered a gene signature predictive of oxaliplatin resistance that was not part of the conventional DNA mismatch repair or RAS/BRAF status markers. When this signature was applied to an independent patient cohort, it successfully stratified patients by progression risk on oxaliplatin, highlighting a new panel of genes for further biological study [403]. On the prognostic front, Guo et al. developed an explainable ML model to predict lung metastasis in colorectal cancer patients by analyzing primary tumor transcriptomes [404]. Their model identified a combination of immunomodulatory and EMT-related genes that foretold a high likelihood of lung metastatic relapse, thus providing both a predictive tool and biological clues (e.g., TGF-β pathway activation) about lung-tropic metastasis mechanisms. In the realm of immunotherapy, although CRC has lagged other cancers, AI is making inroads: a 2021 study used deep learning on pathology images from CRC tumors to predict the presence of tertiary lymphoid structures and high CD8 T-cell densities—features associated with better immunotherapy response—and this image-based model could potentially triage MSS CRC patients for immunotherapy trials even if they are not classically MSI-high [404]. Another exciting example comes from network modeling: researchers constructed a computational model of intracellular signaling in CRC cells and used an evolutionary algorithm (an AI method) to simulate how the network adapts under drug pressure. The simulation predicted that when an EGFR inhibitor is applied, CRC cells will upregulate alternate pathways like MET or IGF1R; this was later confirmed in wet-lab experiments, and importantly, the model suggested a specific dual inhibitor strategy (targeting EGFR and MET simultaneously) which proved highly effective in CRC xenografts [405]. Lastly, in terms of clinical translation, Kleppe et al. developed a deep learning model (“DoMoreAI”) on digitized slides that outperformed conventional histologic grading in prognosticating stage II–III CRC patient survival, which is now being tested in prospective trials as a decision aid for adjuvant chemotherapy [406]. These examples underscore that AI-augmented frameworks are not just theoretical—they are already yielding publishable discoveries in top journals and, in some cases, being deployed in clinical or experimental settings to guide real-world decisions.

***Technical limitations and current challenges***: Despite the impressive advances, the application of AI in CRC research and care faces several challenges. One fundamental issue is the dependence on data quality and quantity. Robust AI models, especially deep learning ones, typically require large, well-annotated datasets. In colorectal cancer, while data are accumulating (TCGA, clinical trials, organoid biobanks, etc.), many AI studies are still limited by cohort size or batch effects between datasets. A predictive model trained on one dataset may not generalize to another if there were differences in sample processing or patient demographics. Overfitting is a constant concern—a model might inadvertently learn patterns specific to a training cohort (e.g., a specific sequencing platform or institutional practice) rather than true biological signals. This leads to another challenge: reproducibility and validation. It is imperative to validate AI models on independent patient cohorts (or external organoid datasets) and, ultimately, in prospective studies. However, such validation is not always carried out or feasible, meaning some published models may not hold up in general use. Another limitation is the interpretability (or lack thereof) of many AI models. Complex algorithms like deep neural networks can act as “black boxes,” making it difficult to understand why a certain prediction was made. In a field like biomedicine, this is problematic—researchers and clinicians need to trust and rationalize model outputs. Techniques in explainable AI (XAI), such as SHAP values or attention maps, are being applied to address this, by highlighting which features (genes, image regions) drove a prediction [407]. Still, bridging the gap between an algorithm’s internal logic and human-understandable biology remains challenging. Additionally, AI models may inadvertently incorporate biases present in the training data. For example, if minority ethnic groups are underrepresented in the data, the model may be less accurate for those populations—a concern for equitable application in CRC, which affects diverse groups [408]. Integration with existing workflows is another hurdle. In the research context, biologists may lack the computational expertise to use AI tools, and conversely data scientists may not fully grasp the biological context, leading to models that miss the mark in terms of useful output. In the clinical realm, implementing AI (say, for decision support) requires changes in workflow and clinician training, and must clear regulatory barriers for safety and efficacy. There are also challenges specific to what is being modeled: for instance, simulating tumor evolution or treatment response is a dynamic, time-series problem, but most available data (biopsies, omics) are static snapshots. AI frameworks thus must extrapolate temporal behavior from static data, which is inherently difficult and can limit accuracy in predicting things like recurrence timing or acquired resistance mechanisms. Privacy and data-sharing issues can impede assembling the large datasets needed for AI, although federated learning (training models across institutions without sharing raw data) is emerging as a solution [409]. Finally, a current challenge is convincing the broader community to view AI predictions as hypothesis-generating rather than definitive—there can be a tendency to either over-hype AI findings or, conversely, to mistrust them entirely [410]. The truth lies in between: AI can point out interesting correlations or candidate mechanisms, but these still require experimental validation and careful peer review. Thus, while AI-augmented frameworks are powerful, one must navigate their limitations by rigorous validation, interpretability efforts, and integration with experimental insight.

***Strategies to address these challenges or integrate with other model systems*:** To realize the full potential of AI in CRC research, several strategies are being implemented. Data curation and sharing is paramount: international consortia are assembling large CRC datasets (combining genomics, imaging, clinical outcomes) and making them available to researchers, which will enable more robust model training [411]. Initiatives to generate high-quality linked data—for example, sequencing organoids and xenografts alongside patient tumors with known treatment outcomes—provide fertile ground for AI model development that connects preclinical and clinical realms [412]. Moreover, adopting standards for data (common file formats, agreed-upon metadata and endpoints) facilitates merging datasets from different studies, effectively increasing sample sizes. On the algorithmic front, researchers are leveraging transfer learning and federated learning to build models that generalize better. For instance, an AI model might be pre-trained on a pan-cancer dataset (to learn general principles) and then fine-tuned on CRC-specific data, which can improve performance when CRC-specific data are limited. To improve interpretability, one strategy is to use knowledge-guided AI: instead of allowing a model to consider all features freely, constrain it using prior biological knowledge. An example would be an AI network that mimics known pathway architectures—such as nodes representing Wnt, MAPK, TGF-β signaling—so that the model’s parameters have biological meaning (this is sometimes achieved with probabilistic graphical models or neural networks with pathway-based nodes). This hybrid approach can yield models that both predict and enlighten (by indicating which pathways or interactions are most perturbed in each tumor). Integration with other model systems is also a key strategy. AI models can act as the “glue” between disparate experimental models: for example, data from 2D cell lines, organoids, PDXs, and patients can be integrated by an AI framework to find common response predictors across models, thereby identifying truly robust biomarkers that transcend a single model system [413]. In practice, this might look like using an AI to analyze a panel of drug responses in cell lines and organoids and then validate the findings in patient data—effectively an in silico meta-analysis that increases confidence in the results. Another integration strategy is in silico trial simulation: combining computational models with clinical trial criteria to simulate how a trial might play out [414]. This can inform trial design (e.g., selecting patient subgroups most likely to benefit, thus making trials more efficient). High-throughput screening data (from organoid chips or CRISPR screens) can feed into AI models to nominate combination therapies, which can then be tested in humanized mice or organ-on-chip setups for functional validation, creating a tri-tier system (AI → ex vivo chip → in vivo model) for vetting therapeutic hypotheses [415]. As for the challenge of implementation in practice, cross-training programs are being put in place—e.g., workshops to teach clinicians and biologists about AI, and conversely to teach data scientists about oncology—fostering the multidisciplinary collaboration needed for successful integration [416]. Efforts to incorporate AI into user-friendly software interfaces or to embed them into existing analysis pipelines also lower the barrier. For example, an AI model for pathology can be integrated into a pathology slide scanner software, so pathologists obtain AI results automatically during their normal workflow. On the regulatory side, there is increasing guidance on validating AI algorithms (e.g., FDA’s proposed frameworks for AI in medical devices), which, once satisfied, will ease clinical adoption [417]. Finally, to ensure AI model recommendations truly bridge to the bedside, some oncology trials are now incorporating an “AI arm”—wherein an AI recommends a therapy and the outcome is observed—to prospectively test the utility of these models [418]. Early success in such trials would be game-changing for acceptance. In summary, by expanding and sharing datasets, using biologically informed and interpretable AI methods, rigorously validating predictions experimentally, and fostering multidisciplinary integration with other model systems, the limitations of AI in CRC research can be overcome. These strategies will enable AI-augmented frameworks to act in concert with humanized mice, organoids, and organ-on-chips—each informing the other—to drive a more efficient and mechanism-driven translational research pipeline. The ultimate vision is a synergistic ecosystem where advanced preclinical models generate data that AI can learn from, and AI in turn guides the next experiments or patient trials, thereby continuously narrowing the gap between bench and bedside in colorectal cancer.

### 2.19. Cross-Scale Integrated Models/Hybrid Models

Cross-scale integrated models are hybrid experimental platforms that combine biological model systems operating at different spatial, temporal, and organizational scales—ranging from subcellular signaling and gene regulatory networks to tissue architecture and systemic interactions—into a cohesive experimental or computational framework. These models are typically constructed through the synergistic integration of 3D organoid cultures, microfluidic systems (organ-on-chip technologies), and AI-based simulations, including multi-omics-informed predictive modeling and machine learning–driven optimization algorithms.

In CRC research, such integrative systems enable the real-time tracking and modulation of tumor progression, immune-microbiome-tumor crosstalk, metastatic transitions, and treatment responses, offering a multi-dimensional, patient-representative experimental landscape. By bridging cellular behavior with tissue-level mechanics and systemic outputs (e.g., drug metabolism, immune infiltration, or microbial influence), cross-scale models address the inherent limitations of single-scale platforms, especially in replicating the heterogeneity, plasticity, and evolutionary dynamics of human CRC.

***Experimental utility in Colorectal Cancer (molecular, therapeutic, and immunologic insights)****:* Hybrid model systems that integrate patient-derived organoids, microfluidic organ-on-chip devices, and AI-driven computational simulations are emerging as powerful cross-scale platforms in CRC research. These integrative models bridge the gap between the cellular microenvironment and whole-patient physiology by combining the 3D tumor architecture of organoids with the dynamic flow and mechanical cues of organs-on-chips, augmented by predictive algorithms. For example, microfluidic “organoid-on-chip” platforms have been used to more faithfully recapitulate the CRC tumor microenvironment. Fang et al. developed a colon tumor organoid chip that mimics intestinal peristalsis, finding that cyclic mechanical strain enhanced stemness (LGR5^+^) and proliferative activity (Ki67^+^) in CRC organoids while reducing uptake of an anti-cancer drug, thereby altering drug efficacy [419]. Likewise, a low-cost OrganoidChip system was shown to support the growth of patient-derived CRC organoids with higher efficiency and maturation than static cultures, yielding larger, more complex organoids without compromising drug response to 5-FU [420].

In the same study, CRC organoids grown on a microfluidic chip formed thicker, multilayered structures with crypt-like projections, compared to cystic, hollow organoids in standard plate culture. The chip-cultured organoids also showed higher formation efficiency and size, indicating a more in vivo-like growth pattern [420]. Beyond tumor epithelium, organoid chips can incorporate physical forces and microenvironment components: a recent bioengineered model combined patient-specific CRC organoids with rhythmic mechanical compression, demonstrating that KRAS-mutant tumors respond to peristaltic forces and even utilize neurotransmitters (like GABA) as an energy source to fuel invasion under those conditions [104]. Such organoid-on-chip systems more closely resemble patient tumors at the transcriptomic level than organoids alone, underscoring their value in modeling CRC invasion and progression.

Advanced AI-driven simulations further enhance these experimental platforms. AI and machine learning algorithms can analyze complex data from organoid and xenograft experiments to optimize treatment strategies in silico. In one approach, an AI-based phenotypic response platform screened dozens of drug combinations on CRC models and successfully identified optimal multi-drug regimens. Notably, a four-drug cocktail (including 5-FU, cetuximab, gemcitabine, and regorafenib) was found to maximally kill tumor cells across CRC organoid lines, circulating tumor cell cultures, and even cells derived from patient xenografts [421]. The AI algorithm efficiently navigated the enormous search space of drug-dose permutations without prior knowledge of drug interactions, illustrating the synergy of AI with wet-lab experiments to discover efficacious therapies.

In a significant advance, researchers have developed “Virtual Patient Avatars via Network Personalization” [422], creating personalized gene regulatory networks for PDX models using genetic algorithms. This approach optimized network alignment with tumor-specific expression profiles, achieving over 90% alignment scores and enabling the identification of patient-specific pathways driving drug sensitivity or resistance, thus demonstrating the feasibility of tailoring therapies to individual tumor networks. Furthermore, machine learning models have been successfully applied to analyze PDX pharmacogenomic data (SNVs, CNAs, GEX) to predict treatment outcomes, particularly in CRC, where multi-gene predictors captured 40% more sensitive PDXs than EGFR mutation status alone [423].

More broadly, AI tools have been integrated with “cancer-on-chip” models for real-time monitoring and decision support. For instance, recent reviews highlight that coupling AI-based image analysis with organ-on-chip devices enables high-throughput, real-time readouts of tumor growth, drug responses, and even spatial immune cell dynamics in CRC models [424]. Such AI-augmented organoid chips raise the prospect of “digital twin” simulations—computational models trained on a patient’s organoid or PDX data—to predict treatment outcomes in silico and refine therapy selection before applying it in patients. This integrative approach leverages the strength of experimental models (faithful tumor biology) and computational power (pattern recognition and optimization) to personalize CRC treatment protocols.

Hybrid platforms are also being employed to study microbiome-immune-tumor interactions in CRC, an area of intense interest given the influence of gut bacteria on tumor behavior and therapy response. Researchers are co-culturing CRC organoids with microbial and immune components on chips to dissect these complex interactions. A recent microfluidic co-culture model seeded colon spheroids in channels perfused with a consortium of gut bacteria to emulate the colonic microbiota. Using this system, Penarete-Acosta et al. investigated the effects of *Fusobacterium nucleatum*—a bacterium enriched in CRC—on tumor cells in the context of other commensal microbes. They found that *Fusobacterium nucleatum* alone triggers pro-carcinogenic changes in colonocytes, upregulating genes for cytokine production, EMT, and proliferation, but many of these effects were markedly blunted when a normal commensal microbial community was present [425]. Interestingly, *Fusobacterium nucleatum* also reshaped the microbial composition, reducing beneficial taxa and potentially fostering a more tumor-promoting microbiota. These findings highlight the context-dependent impact of the microbiome on CRC and underscore the utility of integrated models to study how pathogenic bacteria, host cells, and the immune microenvironment crosstalk. Indeed, multiple studies have singled out *Fusobacterium nucleatum* as a key player: it is frequently abundant in CRC tissues, correlating with poor patient prognosis and chemotherapy failure, and mechanistic work has shown this bacterium can drive chemoresistance. *Fusobacterium nucleatum* binds to TLR4 on CRC cells and activates MYD88 signaling, leading to selective microRNA upregulation that triggers autophagy—a cell survival pathway—thereby protecting tumor cells from chemotherapy-induced death. In vivo and ex vivo experiments confirmed that *Fusobacterium nucleatum* enrichment in tumors promotes resistance to 5-FU and oxaliplatin, consistent with clinical observations that *Fusobacterium nucleatum* is elevated in post-chemotherapy recurrent tumors [85]. Similarly, *Fusobacterium nucleatum* has been shown to inhibit cancer cell pyroptosis (inflammatory programmed cell death) via the Hippo signaling pathway, providing an alternative route by which it blunts chemotherapy efficacy [67,426]. By incorporating bacteria like *Fusobacterium nucleatum* into organoid-on-chip cultures—sometimes alongside immune cells—researchers can directly observe microbiome-mediated modulation of tumor growth and immune responses in real time. These cross-scale models are thus shedding light on the previously opaque tumor–microbiome–immune axis in CRC, revealing how microbial actors might induce immune evasion or drug resistance, and suggesting new therapeutic targets (e.g., blocking TLR4 or autophagy in *Fusobacterium nucleatum*-positive tumors).

***Technical limitations and current challenges:*** Despite their promise, current cross-scale CRC models face significant technical and biological challenges that limit their broader utility.

Integrating organoids with microfluidic chips and AI analytics is technically complex and often low-throughput. Custom organ-on-chip setups require specialized fabrication and handling, and setting up each patient-derived organoid on a chip with perfusion and real-time imaging can be labor-intensive. Scaling these systems for high-throughput drug screens or large patient cohorts remains difficult (each chip may only culture a small number of organoids) [99]. This complexity also raises costs and demands multidisciplinary expertise, making the platforms less accessible to many labs.

Furthermore, a lack of standardized protocols for organoid-on-chip culture leads to variability. Organoids derived from patients can be heterogeneous in size, cell composition, and growth rates. When combined with microfluidic devices, results may vary with subtle differences in chip design (channel geometry, flow rates, matrix composition) or user technique. Reproducing findings across different devices and institutions is challenging without consensus on best practices. Moreover, AI models trained on one dataset or lab’s protocol may not generalize to data from a slightly different platform, hampering reproducibility of AI-driven predictions.

Moreover, while organoids mimic many aspects of tumors, they often lack key components of the native tumor microenvironment. Notably, standard organoids have no vasculature, immune cells, or nerves unless specifically added, and on-chip systems, though they provide mechanical cues and fluid flow, still might not fully recapitulate human organ-level interactions (e.g., endocrine signals or multi-organ metabolism of drugs). The immune contexture is especially critical in CRC, where T cells, macrophages, and other stromal cells influence tumor progression and therapy response. Without these, an organoid-on-chip may respond to drugs differently than an actual tumor in a patient. Similarly, incorporating a complex microbiome is non-trivial—maintaining a stable, representative microbial community alongside mammalian cells on a chip is technically challenging, and often only one or a few bacterial species are introduced. These omissions can lead to models that, despite improvements, still diverge from clinical reality in certain aspects (for instance, an organoid-on-chip might predict a tumor’s chemotherapy response accurately for tumor-intrinsic reasons, but fail to predict immune-mediated effects or toxicity that would occur in vivo).

Lastly, data generated by these hybrid systems can be high-dimensional and complex—time-lapse imaging, multi-omics from organoids, microfluidic sensor readouts, etc. Analyzing and integrating these heterogeneous datasets is non-trivial. AI models require large, well-annotated training data, but given the novelty and custom nature of many organoid-chip experiments, data volumes are often limited. This raises risks of overfitting or biased models. In addition, AI algorithms can behave as “black boxes,” making it hard for researchers to interpret which biological features the model deems important for treatment response [424]. Such opacity can reduce trust in AI-guided protocols and complicate regulatory approval if these models were to be used for clinical decision-making. Finally, there are practical challenges in coordinating wet-lab and computational components—experiments must be designed to generate data that AI can meaningfully use, and conversely AI predictions need to be tested iteratively in the lab. Achieving this feedback loop is logistically demanding.

***Strategies to address these challenges or integrate with other model systems****:* Researchers are actively developing strategies to overcome the above limitations, and several innovations have already demonstrated success in improving the performance and realism of cross-scale models.

To address low throughput, engineers are creating high-density organoid chip arrays that can culture many micro-tissues in parallel. For example, Fang et al.’s peristalsis-mimicking device contains hundreds of microwells to grow colon organoids under uniform mechanical stimulation [419], enabling parallel drug testing. Likewise, “Organoid-on-a-chip” platforms are being made more user-friendly and modular—e.g., prefabricated polymer chips with standard 24-well plate footprints—so that existing laboratory automation (liquid handlers, plate readers) can interface with them [420]. Such designs improve throughput and reproducibility by reducing the need for bespoke setups. Additionally, integration of microfluidic perfusion with robotic handling and real-time imaging has been shown in some systems, allowing automated media exchange, drug addition, and image capture on organoid chips without manual intervention. This automation minimizes user-to-user variability and can run experiments on dozens of patient organoids simultaneously, moving the platform closer to high-throughput screening capabilities.

Additionally, the community is working toward standardizing organoid and organ-on-chip methodologies. Large consortium efforts and biobanks now provide standard operating procedures (SOPs) for organoid culture, genetic characterization, and drug assays. When adapting organoids to chips, researchers have begun publishing detailed protocols (flow rates, matrix composition, timing of interventions) to enable reproducibility. Some studies have demonstrated that results from organoid drug screens can be reproduced in different labs when protocols are harmonized [99]. In the AI realm, using common datasets (e.g., public PDX drug response data or organoid pharmacotyping data) for model training and validation is a mitigation strategy. Shared benchmarking—for instance, the DREAM challenges for drug response prediction—help ensure that an AI model’s performance is robust and not an artifact of a single dataset. Releasing open-source code and pretrained models also improves transparency. As a result, an increasing number of AI tools for tumor modeling adhere to best practices in reporting (dataset size, features used, validation strategy), which builds confidence in their predictions.

Also, a major thrust in improving these models is the addition of immune and stromal elements into organoid-on-chip systems. For example, Neal et al. developed an air–liquid interface (ALI) organoid culture method that preserves tumor-infiltrating lymphocytes within patient-derived tumor organoids (including CRC), enabling ex vivo testing of immunotherapies like anti-PD-1 in the presence of a patient’s own T cells [427]. Such co-culture setups—now sometimes combined with microfluidic chips—allow immune cells to migrate and interact with tumor organoids, recreating the tumor-immune dynamics. Other groups have successfully co-cultured organoids with autologous dendritic cells and T cells to simulate the cancer immunity cycle in vitro, or added cancer-associated fibroblasts to organoid chips to study their effect on drug resistance [428]. To incorporate the microbiome, researchers like Penarete-Acosta et al. have designed anaerobic microfluidic chambers that can sustain commensal bacteria alongside colon organoids [425]. These chips maintain hypoxic conditions and a mucus layer to support bacterial viability, while physical compartmentalization (porous membranes or hydrogel barriers) protects the mammalian cells from overgrowth or direct contact if undesired. By tuning such parameters, it’s possible to model a tumor–microbiome ecosystem and even introduce components of the patient’s own microbiota. Each added cell type or element (immune cells, fibroblasts, bacteria) brings the model closer to physiologic reality and can reveal interactions that would otherwise be missed—for instance, how bacteria suppress or stimulate anti-tumor immune cells. Of course, every addition also increases complexity, so researchers often start with one added component at a time (e.g., organoid + T cells, or organoid + a single microbe) before attempting full ecosystems.

Next, to tackle the data integration challenge, new computational pipelines are being developed that combine mechanistic modeling with AI (a form of “augmented AI”). Rather than treating the organoid-chip as a black box data source, investigators build mechanistic understanding (e.g., via ordinary differential equation models of tumor growth or metabolism under drug exposure) and use AI to fit the parameters or suggest hypotheses. This can reduce the amount of data needed and improve interpretability. Additionally, explainable AI techniques are being applied: for instance, algorithms like SHAP (SHapley Additive exPlanations) or attention-based deep learning models can highlight which input features (e.g., particular gene expression changes in an organoid, or certain cell morphologies on imaging) most strongly influenced a prediction of drug response [429]. By linking these features back to biological processes, researchers can validate whether the AI’s “reasoning” makes sense (e.g., the model might implicitly learn that KRAS mutation status and high EGFR expression correlate with response to an anti-EGFR therapy—insights a human expert can recognize as logical). Such approaches mitigate the black-box issue and have led to discoveries of biomarkers: in one case, a deep learning model analyzing PDX response data identified a set of gene expression changes that predicted chemotherapy resistance, which upon biological follow-up were found to relate to epithelial–mesenchymal transition and immune evasion pathways. This showcases how AI can generate testable biological hypotheses rather than just predictions. Finally, iterative experimental designs—where AI proposes an experiment (e.g., testing a particular drug combo on an organoid) and the new data are used to update the model—create a closed-loop learning system. Early implementations of this (in CRC and other cancers) have successfully converged on potent drug combinations with fewer experiments than brute-force screening [424]. As these virtuous cycles become more commonplace, the predictive power and reliability of cross-scale models will continually improve.

***Future Prospects:*** Looking ahead, cross-scale integrative platforms are poised to become indispensable in CRC research and precision oncology, but realizing their full potential will require several key innovations. First, future systems should strive for complete tumor emulation—this means engineering platforms that not only co-culture tumor organoids with immune and stromal partners, but also capture the spatial and temporal evolution of tumors. *One vision is a “CRC tumor microecosystem on-chip,”* where a patient’s organoid is connected via microfluidic vasculature to modules containing immune cells, gut microbiome, and even tissue from organ-specific niches (like liver organoids to model metastasis). Achieving this will likely involve micro-physiological multi-organ networks: for example, linking a colon tumor chip with a liver chip could allow drugs and tumor-secreted factors to circulate between them, better modeling metastasis and drug metabolism. Early versions of such multi-organ setups have been reported (e.g., coupling liver and tumor organoids to study cancer spread [420,430], describes how chips can be designed with multiple wells for different tissues), but a fully integrated system that includes immunity and microbiota remains an ambitious goal. Innovations in tissue engineering and microfluidics (such as vascularized organoids or endothelial-lined channels that mimic blood vessels) will be critical to supply oxygen and nutrients at scale and to permit immune cell trafficking through the model.

Second, there is a need for high-throughput and high-content integration. The next generation of platforms should marry the throughput of traditional cell-based screens with the realism of organoid-on-chip models. This could involve developing microfluidic plate readers or imaging systems that can rapidly read out dozens of chips in parallel, coupled with AI for image analysis. High-content single-cell analyses (like spatial transcriptomics or Cytometry by Time-of-flight (cy-TOF) imaging) could be incorporated to monitor how tumor subclones or immune cell states evolve over time under treatment—essentially tracking dynamic tumor evolution on the chip. With AI support, one could envision real-time alerts when resistant cell populations emerge in an organoid (detected via morphological changes or gene expression patterns), prompting a clinician or researcher to adjust the treatment in the model. This “adaptive therapy simulation” could guide patient treatment schedules: for instance, if the model predicts that a drug holiday or a switch in drug is needed to prevent resistance, that insight could be tested clinically. To enable this, future platforms will need robust biosensors and live-cell reporters (for apoptosis, proliferation, immune activation, etc.) built into the chips, providing continuous data streams that AI algorithms can analyze on-the-fly.

Third, the field would benefit from an expert system approach that combines human expert knowledge with AI. As more data are amassed from organoid-chip experiments (including drug responses, genomic and microbiome profiles, and patient outcomes), AI could be used to find patterns across patients—for example, identifying that patients with a certain gut microbiome signature and immune cell profile are likely to respond to a specific immunotherapy. These insights might lead to tailored combination therapies: perhaps adding a microbiome-modulating treatment (like an antibiotic or probiotic) to improve immunotherapy efficacy in a subset of CRC patients with *Fusobacterium nucleatum* –rich tumors (a strategy already hinted by evidence that targeting *Fusobacterium nucleatum* or its pathways can restore chemosensitivity [85]. In the future, when a CRC patient is diagnosed, one could rapidly establish an organoid from their tumor, subject it to a battery of drugs on an organ-on-chip platform (with microfluidic perfusion and immune cells present), and simultaneously run a computational model calibrated to that patient’s tumor (“virtual twin”). Within a week, this cross-scale pipeline might output an optimized treatment regimen—not only identifying the most effective drug or drug combination but also forecasting the tumor’s likely evolutionary trajectories (e.g., which resistance mutations or phenotypes might emerge). This information could enable proactive combination therapy, treating the tumor with a cocktail aimed at both killing the bulk of cancer cells and preventing the outgrowth of resistant clones. Some computational oncology frameworks are already moving in this direction, using evolutionary algorithms and longitudinal data to suggest when to switch therapies before resistance blooms.

Finally, to truly enhance clinical translatability, it will be important to demonstrate that these integrative models can predict patient outcomes more reliably than conventional methods. This will likely involve larger validation studies: for instance, a prospective trial where organoid-on-chip assays plus AI predictions are used to guide therapy selection for CRC patients, and the outcomes are compared to a control group treated by standard-of-care decisions. Success in such trials would firmly establish the utility of cross-scale models in the clinic. The integration of advanced computational models, such as the personalized gene regulatory networks for PDX models in “Virtual Patient Avatars via Network Personalization” [422], or the ML-driven multi-gene predictors for PDX drug response [423], can significantly enhance the predictive power of these platforms. An expert proposal for the coming years is to create centralized centers or “tumor simulation cores” equipped with organoid chip technology and AI, where oncologists can send a tumor biopsy and receive a detailed report on predicted drug responses and resistance risks.

In parallel, continued refinement of these models will incorporate emerging science—for example, if new research uncovers that neural innervation of tumors affects CRC progression, future chips might integrate enteric nerve cells alongside organoids. The flexibility of the organoid-on-chip approach means it can evolve with our understanding, making it a living platform for testing hypotheses about CRC biology. In conclusion, cross-scale integrated models represent a convergence of bioengineering, computer science, and cancer biology that is transforming CRC research. By faithfully reproducing tumor complexity and coupling it with smart algorithms, they offer a route to high-throughput, physiologically relevant drug screening, deep immune profiling in vitro, and tracking of tumor adaptation over time—all tailored to individual patients. Overcoming current challenges through innovation and collaboration will pave the way for these models to accelerate drug discovery and bring truly personalized, dynamic treatment strategies to the forefront of colorectal cancer care. The momentum in this field suggests that the vision of “clinical trials on a chip”—testing therapies on a patient’s mini-tumor and its microenvironment before treating the patient—is on the horizon, heralding a new era of integrative oncology research [431]. Each refinement in these platforms brings us closer to that goal, with the ultimate payoff being improved outcomes and survival for CRC patients through smarter, data-driven intervention strategies.

## 3. Future Directions

***Affordable and Scalable Colorectal Cancer Model Systems for Low-Resource Settings:*** A key future goal is to democratize advanced CRC models by reducing cost and complexity without sacrificing translational relevance. Current high-end models like PDXs and organoids rely on expensive reagents and infrastructure, limiting their use in low-resource settings. To overcome this, researchers are developing economical alternatives such as zebrafish xenograft models, which offer a rapid in vivo platform for tumor growth and drug testing at a fraction of the cost of mouse models [432]. Zebrafish are ~87% genetically similar to humans and can absorb compounds directly from water, enabling high-throughput drug screens in a living system. They thus serve as a bridge between simple in vitro assays and expensive rodent studies, allowing labs with limited resources to perform in vivo therapeutic evaluation [231]. In parallel, efforts are underway to simplify 3D organoid culture by using synthetic or plant-derived matrices and cost-effective growth factor substitutes, aiming to create “organoid kits” that can be widely adopted. By standardizing protocols and establishing regional biobanks, organoids could be shared across institutions, reducing the need for each lab to develop its own models from scratch. Additionally, open-source microfluidics and 3D-bioprinted scaffolds are being explored to produce CRC-on-a-chip systems using affordable materials. These approaches, combined with training programs and tech-transfer to developing regions, will pave the way for scalable CRC models that global researchers can use for drug screening and mechanistic studies. Ultimately, making model systems more accessible and scalable will accelerate CRC research and therapeutic testing worldwide.

***AI and In Silico Platforms for Mechanistic Insights and Drug Discovery:*** Emerging artificial intelligence tools are set to revolutionize CRC model development by offering powerful in silico complements to laboratory models. Machine learning algorithms can analyze vast datasets from genomics, proteomics, and pharmacogenomic screens to predict new drug targets and optimize therapy combinations [433]. In CRC, various deep learning models have already been used for therapeutic decision support and drug repurposing analyses. Future model systems will integrate AI-driven simulations to guide experiments—for example, predicting how a particular KRAS or p53 mutation alters signaling network dynamics and drug sensitivities, which can then be validated in organoids or cell lines. A particularly transformative advance is the use of AlphaFold and next-generation protein folding AI for structural biology. AlphaFold2 has demonstrated unprecedented accuracy in predicting 3D structures of cancer-relevant proteins, and the latest iteration (AlphaFold3) can even model protein–protein and protein–ligand complexes [434]. These capabilities enable researchers to perform virtual docking studies on mutant CRC proteins or identify cryptic binding pockets for difficult targets (e.g., undruggable oncogenes). By integrating these structural insights, one can design or screen novel inhibitors in silico before testing them in vitro. Moreover, AI can help model tumor behavior: for instance, digital twin simulations of tumor growth could be used to predict how a patient’s cancer might respond to immunotherapy or evolve resistance, informing the design of corresponding in vitro models. Looking forward, a tight coupling between computational and experimental platforms will greatly accelerate drug discovery—AI will propose candidate compounds or therapeutic strategies, and advanced CRC models will verify efficacy and elucidate mechanisms. This iterative loop can significantly cut down the cost and time of developing new CRC therapies [433], an especially important benefit for academic and low-resource settings.

***High-Throughput Model Platforms Emulating the Tumor Niche:*** Next-generation CRC models are being engineered for high-throughput drug screening while faithfully recapitulating the complex tumor microenvironment. Traditional 2D monocultures cannot mimic the three-dimensional architecture, signaling gradients, and cell–cell interactions of tumors, prompting development of advanced 3D culture systems. Organoid-based screening platforms are leading this area: CRC organoids grown in multi-well formats or microfluidic devices can be exposed to large panels of drug candidates to identify effective treatments. Recent innovations allow incorporation of crucial tumor microenvironment components into these screens. For example, co-culturing organoids with autologous immune cells (T lymphocytes, NK cells) or fibroblasts produces immune-enhanced organoids that respond to immunotherapies, helping evaluate checkpoint inhibitors or CAR-T cells in a patient-specific context. Similarly, organoids can be embedded in perfused microfluidic chips that supply nutrients and oxygen gradients, recreating in vivo-like signaling and drug distribution. Microfluidic organ-on-chip systems also permit the introduction of shear stress and flow, modeling conditions like those in the gut vasculature. In tandem, 3D bioprinting is being used to fabricate miniaturized tumor tissues with controlled architecture, printing cancer cells together with extracellular matrix components and stromal cells [435]. Such printed mini-tumors can be made in arrayed formats for parallel testing of hundreds of compounds. High-content imaging and single-cell analyses can then read out not only viability but also pathway activation, differentiation states, and immune cell infiltration in each model. Notably, these high-throughput platforms strive to capture intratumoral heterogeneity—for instance, by starting from multi-clonal organoid cultures or mixing cells from different tumor regions—to ensure that drug responses reflect the varied cell subpopulations present in real tumors. While challenges remain (e.g., maintaining long-term stability and the full spectrum of immune cell diversity [436]), future improvements, including integrating AI to analyze results and multi-omics readouts (genomic, transcriptomic, etc.), are expected to greatly enhance the predictive power of these model systems [435]. Ultimately, high-throughput models that closely emulate the in vivo CRC niche will enable rapid, cost-effective screening of new therapeutics (or combinations) with much greater translational relevance than conventional cell-line assays.

***Modeling Tumor Evolution and Metastatic Spread*:** Colorectal cancer progresses through multiple stages—from early adenoma to localized carcinoma, and eventually to distant metastases (commonly to liver and lung)—yet most model systems capture only snapshots of this progression. A forward-looking strategy is to develop models that track CRC evolution over time and across sites, enabling study of how tumor cell phenotypes and vulnerabilities change during disease advancement. One approach is longitudinal organoid modeling, where serial samples from the same patient (e.g., primary tumor, post-therapy recurrence, and metastases) are used to establish organoids that can be compared and passaged over time. Such paired organoid models have demonstrated that metastatic lesions can harbor distinct cellular states and drug sensitivities compared to their primary tumor counterparts [437]. By examining organoids derived from liver or lung metastases alongside those from primary colon tumors, researchers can pinpoint genetic or epigenetic adaptations that underlie organ-specific tropism. For example, liver metastasis organoids may be co-cultured with hepatocytes or liver sinusoidal endothelial cells to observe how tumor cells exploit the hepatic niche. Likewise, lung-tropic CRC organoids can be exposed to pulmonary alveolar conditions (such as high oxygen) to study their adaptability. Future micro-physiological models might even connect colon organoids with mini-liver and mini-lung tissues in one circuit, to simulate the process of metastatic dissemination and colonization in real time. On the in vivo front, new mouse models are being created to mirror the stepwise progression of CRC metastasis. These include genetically engineered mouse models that develop spontaneous liver or lung metastases, and refined orthotopic models where CRC cells are implanted into the cecum or spleen of mice to seed metastatic outgrowth in a natural manner [438]. Such models offer the ability to monitor tumor evolution under the pressure of an intact immune system and organ environment. Additionally, evolutionary pressure experiments in vitro—for instance, exposing organoids to intermittent chemotherapy to select for resistant clones—can illuminate how tumors adapt and which molecular pathways drive progression or drug resistance over time. Integrating these approaches, future CRC models will not be static but will represent a dynamic timeline of the disease. This will greatly aid in identifying stage-specific targets (e.g., metastasis suppressors) and in testing interventions that prevent early-stage lesions from acquiring aggressive traits. Importantly, modeling metastasis (especially to liver and lung) is crucial for improving late-stage CRC outcomes; by replicating these distant microenvironments in the lab, researchers can discover vulnerabilities of metastatic cells that would be missed in primary-tumor-only models [437]. Such insights will guide the development of therapies tailored to advanced CRC and perhaps strategies to preempt metastasis in high-risk patients.

***Nutraceuticals and Combination Therapies in Colorectal Cancer Models***: There is growing interest in integrating nutraceuticals—bioactive compounds derived from diet or plants—into CRC model systems to explore their preventive and therapeutic potential, especially in combination with standard drugs. Nutraceuticals like curcumin (from turmeric), resveratrol (from grapes), and epigallocatechin-3-gallate (from green tea) have shown anti-CRC effects in preclinical studies and can modulate pathways linked to inflammation, apoptosis, and metastasis [439]. Future CRC models are poised to evaluate such compounds more systematically, both as single agents and as adjuvants that enhance conventional therapies. Notably, nutraceuticals often target multiple cellular pathways with low toxicity, which could help overcome chemotherapy resistance and cancer cell plasticity [440]. For instance, resveratrol has been identified as a potent chemosensitizer that can re-sensitize CRC cells to 5-FU and oxaliplatin by modulating signaling networks involved in drug resistance and stemness. Incorporating resveratrol or similar compounds into organoid drug screens has revealed synergistic effects—studies show that combining resveratrol with standard chemotherapeutics significantly inhibits CRC cell growth and prevents emergence of resistant clones. Future organoid models might include nutraceutical-enriched culture conditions to simulate a “chemopreventive” microenvironment and analyze how chronic exposure to dietary agents alters tumor behavior or response to drugs. Our, recent work (IJMS 2025) demonstrated the power of nutraceuticals to interfere with pro-tumor inflammatory signaling: oleocanthal, a phenolic compound from extra-virgin olive oil, was shown to selectively modulate the PAR-2 (protease-activated receptor-2) pathway in CRC cells, resulting in attenuation of TNF-α secretion and restoration of calcium homeostasis [441,442]. This led to suppressed activation of downstream oncogenic pathways and highlights how a dietary compound can act on the same inflammatory targets as pharmaceutical drugs. Going forward, CRC models will be used to explore drug–nutraceutical synergies: for example, combining an anti-EGFR antibody with a polyphenol to achieve additive anti-proliferative effects, or using omega-3 fatty acids alongside immunotherapy to mitigate treatment-related inflammation. The integration of nutraceutical research into CRC modeling not only reflects a push toward more holistic, low-toxicity treatment strategies but also is especially relevant for low-resource regions, where certain nutraceuticals are readily available and affordable. By validating these agents in predictive models (organoids, spheroids, or even in vivo dietary intervention models), researchers can provide a scientific basis for novel adjunct therapies and inform clinical trials for precision nutrition approaches in CRC management.

***Repurposed Drugs and Inflammation-Driven Colorectal Cancer Models:*** Exploiting existing drugs for new anti-cancer uses—drug repurposing—is a promising avenue that future CRC models will actively support. Many common medications (e.g., anti-diabetic, anti-hypertensive, or anti-inflammatory drugs) have unanticipated anti-tumor effects that can be systematically identified using advanced models. A prime example is the class of statins, widely used as cholesterol-lowering agents, which epidemiological studies have linked to reduced CRC incidence and improved patient survival. Cutting-edge CRC models are now shedding light on the mechanisms behind this observation. In particular, chronic inflammation is known to drive colorectal tumorigenesis (as seen in colitis-associated cancer), and one inflammatory axis involves PAR-2, a receptor that when activated (by proteases or tissue injury) triggers pro-tumorigenic signaling cascades. Recent research using CRC cell line models revealed that atorvastatin and rosuvastatin directly target this pathway: these statins selectively downregulated PAR-2 expression and markedly reduced TNF-α secretion from CRC cells, while also suppressing aberrant calcium signaling linked to PAR-2 activation [34]. This led to an overall dampening of the inflammatory microenvironment and slowed cancer cell proliferation, underscoring the potential of statins as adjunctive anti-inflammatory agents in CRC. The ability of a repurposed cardiovascular drug to modulate a cancer-driving inflammatory loop exemplifies the kind of insight future models can deliver. Moving forward, CRC organoids co-cultured with immune cells (to produce cytokines) or stimulated with bacterial endotoxins (to simulate gut inflammation) will serve as in vitro platforms to evaluate anti-inflammatory or immune-modulating drugs. These inflammation-enhanced models can mimic conditions like inflammatory bowel disease leading to CRC, allowing testing of candidates such as NSAIDs, IL-6/JAK-STAT inhibitors, or novel PAR-2 antagonists in a controlled setting. Coupled with transcriptomic profiling, such studies will identify how repurposed drugs rewire the tumor–immune interface. Beyond statins, other drugs are on the radar for repurposing in CRC: metformin (an antidiabetic) has been shown to inhibit CRC stem cell growth by affecting AMPK/mTOR signaling, and aspirin (a traditional NSAID) is known to prevent CRC partly by blocking COX-2 mediated prostaglandin E2 production. Future CRC models—including genetically engineered mice that develop tumors in the context of chronic colitis, or humanized mouse models with patient-derived tumors and immune systems—will be invaluable to test these agents and their combinations. Importantly, by modeling the inflammation-driven progression of CRC, researchers can better understand how interventions at the level of inflammatory signaling (like PAR-2 blockade) might halt or reverse tumor development. The convergence of evidence from nutraceutical studies (e.g., oleocanthal) and drug repurposing studies (e.g., statins) on common inflammatory pathways (such as PAR-2, NF-κB, and cytokine networks) is particularly exciting. It suggests that future therapies could combine lifestyle components with safe existing drugs to synergistically defuse pro-tumor inflammation. Advanced CRC model systems will be the testing ground for this strategy—helping to optimize doses, timing, and combinations of repurposed drugs and nutraceuticals that most effectively curtail inflammation-driven tumor growth. By leveraging what is already pharmaceutically available, this approach could rapidly translate into cost-effective treatments to improve CRC prevention and patient outcomes.

## 4. Conclusions

The landscape of CRC modeling has evolved into a richly layered ecosystem where in vivo, in vitro, and in silico platforms serve as distinct yet increasingly interconnected modalities. This review has undertaken a rigorous classification of these models—spanning humanized mouse avatars, organ-on-chip biointerfaces, AI-augmented computational simulators, and diverse animal systems ranging from zebrafish to porcine hosts—with the intent of clarifying their mechanistic depth, translational viability, and contextual limitations.

What emerges is a schema of strategic complementarity: while no single model recapitulates all aspects of human CRC pathogenesis or therapeutic response, each offers uniquely valuable insights. For instance, humanized PDX mouse models, such as those developed by Kanikarla-Marie et al., accurately simulate immune checkpoint blockade in a patient-specific context—an achievement unreplicated by canonical xenografts [191]. Meanwhile, tumor-on-chip systems, such as the 3D-printed microfluidic arrays by Steinberg et al., have demonstrated the capacity to recapitulate personalized chemotherapy responses, integrating pharmacokinetic realism and stromal complexity [362]. Simultaneously, AI-augmented frameworks—particularly digital twin models and pathway-informed neural nets—have successfully stratified patients for chemotherapy or immunotherapy based on multi-omics and radiopathological correlates, thus closing the loop between predictive insight and actionable treatment planning.

This dynamic interplay underscores the necessity of transdisciplinary synergy. Models must no longer exist in silos; instead, iterative workflows that span across platforms—e.g., using AI to prioritize treatment regimens which are first tested in organ-on-chip systems and subsequently validated in humanized in vivo model, i.e., *the workflow: in vitro mechanism → chip validation → in vivo translation* is emerging as gold standard. Case in point, a recent translational CRC study demonstrated the power of integrating PDOs, OoC platforms, and PDX models to unravel KRAS-driven tumor biology. Researchers began with mechanistic in vitro experiments using OoC systems, which more faithfully recapitulated patient tumor transcriptional profiles than conventional static organoid cultures. Employing omics technologies and live-cell imaging, they identified that KRAS-mutant CRC cells responded markedly to biomechanical cues present in the tumor microenvironment. In particular, under conditions simulating physiological flow and peristalsis, these tumor cells exploited γ-aminobutyric acid (GABA) as an alternative metabolic substrate, thereby enhancing their invasiveness. These mechanistic insights were then corroborated in PDX models, affirming the translational validity of the chip-based findings and underlining the clinical potential of targeting KRAS-mutant CRC through metabolic and biomechanical pathways biology [104]. Similarly, in another study, investigators developed a sophisticated multiplexed tumor-on-chip device featuring an 8 × 4 array integrated with a concentration gradient generator to enable high-throughput drug screening. In the in vitro discovery phase, patient-derived tumor spheroids obtained from three individuals with CRC were exposed to five standard-of-care chemotherapeutic agents. The tumor-on-chip system allowed for precise dose–response profiling across the drug panel. Notably, the chip-based validation revealed consistent, dose-dependent responses which were then benchmarked against matched PDX models [365]. Quantitative comparison showed a strong correlation between the drug efficacy profiles obtained from the chip and the in vivo PDX outcomes, with correlation coefficients spanning from 0.40 to 0.90 across the three patient samples [443]. This study underscored the translational fidelity of the chip-based screening approach, effectively bridging in vitro therapeutic profiling with in vivo response prediction. Building upon these findings, another study employed a vascularized micro-tumor (VMT) platform to model CRC in a physiologically relevant microenvironment. In the in vitro mechanistic phase, researchers conducted transcriptomic profiling of 770 cancer-associated genes across 13 signaling pathways using HCT116 CRC cells cultured under varying conditions. The chip-based validation revealed that tumor cells grown within the VMT system exhibited gene expression profiles that closely mirrored those of xenograft-derived tumors, with no statistically significant differences observed. Notably, key oncogenic pathways such as PI3K-Akt signaling, MAPK signaling, and epithelial-to-mesenchymal transition were consistently enriched in both the VMT and xenograft-derived CRC cells. In the final translational phase, tumor growth kinetics in the VMT closely paralleled those of xenografts, and administration of the FOLFOX chemotherapy regimen yielded concordant therapeutic responses in both models. This study further reinforced the predictive accuracy and translational value of OoC systems in preclinical CRC research [369]. In a further advancement of translational modeling, researchers engineered a sophisticated microfluidic platform to investigate the effects of dynamic, physiologically relevant drug exposure profiles in CRC. The in vitro mechanistic phase focused on contrasting static drug administration with pharmacokinetic profiles that mimic in vivo conditions. During the chip validation phase, the device delivered precisely controlled drug concentrations through continuous flow systems, and the accuracy of these profiles was confirmed using fluorescence-based tracking and computational simulations [444]. HCT116 CRC cells were treated with oxaliplatin under both static and dynamic conditions. The study revealed that cells exposed to dynamic, clinically relevant drug profiles exhibited significantly reduced growth compared to untreated controls. Notably, continuous exposure to mean drug concentrations proved more effective than short bursts of peak concentrations, underscoring the importance of exposure kinetics in therapeutic efficacy. These findings offered valuable insights for optimizing drug dosing regimens in clinical oncology. Furthermore, researchers engineered biofabricated CRC tissue equivalents to replicate the complex architectural features observed in patient biopsy specimens. In the in vitro mechanistic phase, they demonstrated that the organization of the ECM, critically influenced CRC cell behavior, aligned ECM fibers within tumor-stroma co-cultures promoted epithelial phenotypes, whereas disordered ECM configurations induced mesenchymal traits. The chip-based validation phase confirmed that these architectural and phenotypic features could be faithfully preserved within controlled microfluidic environments, enabling reproducible manipulation of tumor-stroma interactions. In the in vivo translation phase, implantation of these engineered co-cultures into murine models recapitulated tumor growth dynamics and preserved ECM architecture for up to 28 days. This study was the first to successfully demonstrate the replication of human tumor ECM architecture across ex vivo and in vivo systems, thus validating the translational relevance of structurally engineered tumor models in CRC research [445]. Moreover, a novel microfluidic model was devised to investigate the intricate interactions between colonocytes and gut microbiota in the context of CRC. In the in vitro mechanistic phase, researchers focused on elucidating how *Fusobacterium nucleatum* modulates colonocyte gene expression and alters the surrounding microbial community. During the chip validation phase, spatial separation of colonocytes and microbial populations was achieved using porous membranes under continuous perfusion, allowing stable and physiologically relevant co-cultures. The study revealed that *Fusobacterium nucleatum* could induce transcriptional changes in genes related to cytokine secretion, epithelial-to-mesenchymal transition, and cellular proliferation, all through contact-independent mechanisms [425]. While this work remained within the in vitro and chip-based domains, it laid a critical foundation for future in vivo translational studies aimed at developing microbiome-targeted therapies for colorectal cancer. In summary, the studies reviewed demonstrate several key advantages of following the in vitro mechanism → chip validation → in vivo translation blueprint: (A) Enhanced predictive power: OoC models showed closer transcriptional resemblance to patient tumors compared to static cultures, with 76% average accuracy in predicting patient responses; (B) Reduced animal usage: Chip validation allows for extensive screening and optimization before in vivo studies, reducing the number of animal experiments required; (C) Personalized medicine integration: The workflow enables patient-specific model development using primary tissues, facilitating personalized treatment strategies; and (D) Mechanistic insights: The combination approach provides deeper understanding of biological mechanisms while maintaining clinical relevance.

Consequently, the convergence of these platforms enables a recursive learning cycle wherein wet-lab findings inform computational refinement, and vice versa. Translational relevance, long the Achilles’ heel of preclinical cancer research, is increasingly being addressed through hybrid approaches. The integration of patient-derived material into both organoids and mouse avatars, alongside real-time data analytics and biomarker discovery through AI, now makes it feasible to envisage “avatar-guided clinical trials”—trials whose therapeutic hypotheses are stress-tested across layers of experimental fidelity before reaching the patient bedside [446].

As we refine these models further, future innovations should not only enhance model fidelity, but also improve interoperability, scalability, and ethical accessibility. Federated learning frameworks, microphysiological system standardization, and organoid-chip interfaces seeded with autologous immune components are likely to become linchpins in this translational continuum.

All things considered, the future of CRC modeling is not about choosing the “best” model, but about understanding how to strategically integrate model systems—leveraging their complementary strengths to construct a coherent, multi-modal, and patient-relevant translational pipeline. This will be the crucible within which next-generation therapeutics, biomarkers, and diagnostic algorithms for colorectal cancer are forged.

## Figures and Tables

**Table 1:** Comparative analysis of in vitro models for colorectal cancer research.

In Vitro Model	2D Cell Lines	Cell Line Spheroids (MCTS)	Patient-Derived Organoids (PDOs)—Standard Culture	Advanced PDO Models (Co-Culture/OoC/ALI)
Source Material	Immortalized lines (Patient/Xenograft origin)	Immortalized lines	Patient Tissue (Tumor/Normal; Biopsy/Resection)	Patient Tissue + TME Cells/Microfluidic Device
Generation Method	Monolayer culture on plastic/glass	Self-aggregation (Scaffold-free/based)	Self-organization from stem/progenitor cells in ECM	Co-culture, Microfluidics, ALI methods
3D Architecture	Absent (Monolayer)	Basic (Spherical aggregate)	Complex (Organotypic, Glandular)	Complex + TME integration/Perfusion
Cellular Heterogeneity (Epithelial)	Low (Often clonal)	Low-Medium (Source line dependent)	High (Reflects patient tumor)	High (Reflects patient tumor)
TME Representation	Minimal/Absent	Limited (Absent unless co-cultured)	Limited (Epithelial only)	Medium-High (Incorporates TME cells/factors)
Cell–Cell Interactions	Limited/Artificial	Moderate (3D proximity)	High (Physiological)	High (Physiological + TME)
Cell-ECM Interactions	Artificial (Plastic)	Limited (Endogenous ECM)	Medium (ECM substitute—Matrigel)	Medium-High (ECM substitute + TME matrix/forces)
Physiological Gradients	Minimal	Present (Size-dependent)	Present (Complex morphology)	Present and Controllable (OoC)
Genetic/Epigenetic Fidelity	Low (Drift prone)	Low-Medium (Source line dependent)	Very High (Closely matches patient)	Very High (Maintains PDO fidelity)
Scalability	★★★★★ (Very High)	★★★☆☆ (Medium, Method-dependent)	★★☆☆☆ (Low-Medium)	★☆☆☆☆ (Low, Complex)
Throughput	★★★★★ (Very High)	★★★☆☆ (Medium)	★★☆☆☆ (Low-Medium, Improving)	★☆☆☆☆ (Low)
Reproducibility	★★★★☆ (High)	★★☆☆☆ (Variable, Size control issue)	★★☆☆☆ (Patient variability, Protocol dependent)	★☆☆☆☆ (Complex, Less standardized)
Cost	★★★★★ (Very Low)	★★★★☆ (Low-Medium)	★★☆☆☆ (High)	★☆☆☆☆ (Very High)
Time Requirement	★★★★★ (Short)	★★★★☆ (Moderate)	★★☆☆☆ (Long establishment, Moderate testing)	★☆☆☆☆ (Long, Complex setup)
Technical Expertise Required	★★★★★ (Minimal)	★★★★☆ (Moderate)	★★☆☆☆ (High)	★☆☆☆☆ (Very High)
Suitability: HTS	★★★★☆ (Standard, but low relevance)	★★★☆☆ (Feasible, better relevance)	★★☆☆☆ (Possible, improving)	★☆☆☆☆ (Difficult)
Suitability: Personalized Med.	☆☆☆☆☆ (Not Suitable)	☆☆☆☆☆ (Not Suitable)	★★★★★ (Excellent, Predictive potential)	★★★★★ (Potentially enhanced prediction)
Suitability: Mechanistic Studies	★★☆☆☆ (Basic pathways)	★★★☆☆ (Gradient/3D effects)	★★★★☆ (Patient-relevant context)	★★★★★ (Complex interactions, TME)
Suitability: Metastasis Studies	★☆☆☆☆ (Limited)	★★☆☆☆ (Invasion assays)	★★★☆☆ (Invasion, Requires PDOX/OoC)	★★★★☆ (OoC for cascade steps)
Suitability: Biomarker Discovery	★☆☆☆☆ (Low relevance)	★★☆☆☆ (Limited)	★★★★★ (Excellent, High fidelity)	★★★★★ (Excellent, TME context)
Suitability: IO/TME Studies	☆☆☆☆☆ (Not Suitable)	★★☆☆☆ (Heterotypic spheroids only)	★☆☆☆☆ (Requires co-culture)	★★★★★ (Ideal platform)
Clinical Predictivity	★☆☆☆☆ (Poor)	★★☆☆☆ (Limited evidence)	★★★★☆ (Good, Validated correlations)	★★★★★ (Potentially Highest, Needs validation)
Key Limitations	Relevance, Simplicity	Complexity, Fidelity, TME	TME absence, Scalability, Cost, TAT, Standardization	Complexity, Cost, Standardization, Throughput

**Table 2:** Comparative analysis of in vivo models for colorectal cancer research.

Species	Model Description	Human CRC Relevance	Genetic Manipulability	Cost and Maintenance	Imaging and Access to Tumors	Translational Utility	Major Limitations	Ref
**Mouse (*Mus musculus*)**	20–30 g, ~2 yr lifespan; used in CRC research (carcinogen-induced, GEMMs, xenografts, syngeneic, orthotopic implants)	High relevance for CRC; ~95% genetic homology. Murine tumors recapitulate key aspects of human CRC, including polyposis and liver metastasis.	Highly tractable for genetic engineering (transgenic, knockout, CRISPR). Rapid breeding.	Moderate cost; more cost-effective than larger animals, but can be expensive for sophisticated GEMMs or carcinogen-induced models.	Small size necessitates advanced imaging (endoscopy, bioluminescence, micro-CT/MRI). Subcutaneous tumors are readily measurable.	Primary platform for preclinical therapeutic testing. Facilitates toxicology and pharmacokinetic studies. Critical for evaluating immune therapies.	Species-specific differences in immune system, metabolism, and gut microbiome. Many models only partially mimic human CRC (e.g., *ApcMin* model). Scale limitations. Predictive validity varies.	[13,14,15]
**Zebrafish (*Danio rerio*)**	Small aquatic vertebrates (3–5 cm); optically transparent embryos; rapid life cycle (3–4 months). Genetic models (APC/KRAS mutation) and xenograft models used for CRC.	Moderate relevance for human CRC. Conserved cell types and pathways. Tumors share morphological and molecular characteristics with human adenomas. Cancer cell behaviors (migration, angiogenesis) parallel mammals.	Significant genetic tractability; facile manipulation via external fertilization. Efficient gene knockout (CRISPR, morpholino). Large clutch sizes facilitate high-throughput studies.	Highly cost-effective; dozens of adult fish use same space as one mouse. Simple aquatic setups for embryos/larvae.	Excellent visualization in larvae due to transparency. Real-time in vivo imaging of tumor cells.	Moderate translational utility; serves as discovery tools and filters. Valuable for drug discovery and screening. Excellent for studying early tumorigenesis and cell migration.	Temperature and physiology mismatch (xenografts tested at 28 °C vs. 37 °C human). Lack of adaptive immune system in early larval stages. Fish-specific biology (aquatic microbiome, regenerative capacity) can lead to non-human tumor responses. Adult fish tumors are difficult to manipulate/assess without sacrifice	[16,17,18]
**Drosophila (*Fruit fly*)**	~3 mm, ~10-day life cycle; invertebrate model for intestinal tumorigenesis. Engineered loss of tumor suppressors (e.g., APC homolog) or activation of oncogenes (e.g., RasV12) in intestinal stem cells.	Low-to-moderate relevance due to evolutionary distance. Conserves core cancer pathways (e.g., Wnt, EGFR/Ras, Hippo, JNK). Fly midgut tumors mimic cellular aspects of human CRC.	Unparalleled genetic control; facile combination of multiple mutations. Tissue-specific gene expression/knockdown (Gal4-UAS, RNAi libraries). Whole-genome screens feasible; CRISPR routine.	Exceptionally inexpensive; simple media; basic experiments need only dissection microscopes. Large-scale screens economically feasible.	Limited in vivo imaging without advanced techniques; typically post-mortem dissection. Fluorescent reporters with confocal microscopy allow cellular-resolution.	Low direct translational value; primarily for gene discovery and pathway dissection. Can inform mammalian research by identifying novel targets. Limited for direct drug efficacy testing.	Lack adaptive immunity. Drastically different physiology; no true colon structure or similar microbiome. Scale and endpoint assessment challenging. Some human CRC drivers have divergent roles or no clear fly counterparts.	[19,20,21]
**Dog (*Canis familiaris*)**	Pet dogs (10–40 kg) with low incidence (<1%) of spontaneous CRC. Tumors often in distal colon/rectum, mimicking human adenoma-carcinoma sequence.	Highly relevant for specific CRC aspects. Share mammalian physiology, diet, environmental exposures. Spontaneous tumors develop over years with intact immune system, mirroring human sporadic CRC. Histologically and molecularly similar to human CRC.	Very limited genetic manipulation; relies on natural variation. No transgenic/knockout dogs for CRC. Ethical considerations preclude induced models.	Highly expensive due to space, food, and veterinary care. Comparative oncology clinical trials are costly.	Good opportunities for tumor assessment; full human clinical imaging modalities (colonoscopies, CT/MRI). Allows in vivo monitoring of tumor development/treatment response. Access to tumor tissue via biopsy/surgery.	High translational potential, especially for immunotherapies and drug side effects. Drug pharmacokinetics often scale to humans. Offer insights into long-term outcomes in immunocompetent, heterogeneous population.	Low incidence and limited case availability. Genetic heterogeneity. Ethical constraints limit tissue collection and experimental design (no untreated controls). Disease differences (low incidence, distinct risk factors)	[22,23,24]
**Pig (*Sus scrofa*)**	Large omnivorous mammals (50–300 kg; mini-pigs ~20–40 kg); long lifespan (10–15 years). Genetically engineered models (e.g., APC^1311 mutation, Oncopig with KRAS/TP53 mutations). Spontaneous CRC rare.	High relevance due to anatomical/functional similarities to human colon. Porcine polyps/tumors resemble human lesions in structure/molecular alterations. Greater metabolic and microbiome similarities to humans than rodents. Recapitulates clinical scenario of human tumors in size, location, progression.	Low-to-moderate challenge. Gene-editing (CRISPR) and cloning enabled mutant lines, but slower breeding. Conditional/inducible systems enhance control. Still far from ease of mouse genetic manipulation.	Exceptionally expensive due to high housing and feed costs. Veterinary care adds significantly. Few specialized centers.	Fair opportunities for tumor assessment; accommodates identical clinical imaging tools as humans (colonoscopy, CT, MRI, PET). Multiple biopsies and experimental surgeries feasible. Longitudinal access for biopsies over time.	Excellent preclinical validation model with high translational potential. Human-equivalent scale allows testing of new endoscopic techniques, surgical devices, radiotherapy. Pharmacokinetic/toxicity studies often more predictive than rodent data.	Small sample sizes due to high costs. Long tumor latency. Incomplete disease spectrum (APC-mutant pigs primarily adenomas). Handling/welfare concerns (requires anesthesia). Fewer molecular tools compared to mice.	[25,26,27]
**Non-Human Primate (e.g., *Rhesus macaque*)**	No standard experimental CRC model; spontaneous cases observed. Some rhesus macaques develop early-onset CRC with MMR deficiencies (Lynch syndrome analog). Cotton-top tamarins develop ulcerative colitis and subsequent CRC. Long lifespan (20–30 years).	Very high relevance due to close physiological similarities. Spontaneous tumors have nearly identical histology and metastatic patterns to human CRC. Molecular genetics mirror human pathways (MMR, APC). Colonic structure, microbiota, aging processes highly comparable.	Extremely limited genetic manipulation. No ability to create/breed NHPs specifically for CRC research. Relies on serendipitous findings or colitis induction.	Extremely high cost; specialized primate facilities, skilled veterinary staff. Annual care can be tens of thousands of dollars.	Good opportunities for assessment; identical clinical diagnostic approaches as humans (endoscopy, biopsy, full imaging). Allows in vivo monitoring. Longitudinal monitoring possible for rare spontaneous cases.	Moderate direct translational utility, primarily due to close immunological similarity. Valuable for testing preventive vaccines or immune interventions. Important for assessing therapy safety.	Ethical and regulatory barriers severely restrict intentional CRC research. Minuscule sample sizes. Confounding factors within NHP colonies (pathogens, diet, stress). Very long timelines. Public scrutiny of primate experiments	[28,29]

**Table 3:** Comparative analysis of emerging integrated models for colorectal cancer research.

Dimension	Humanized Mouse Models	Microfluidic Tumor-on-Chip Systems	AI-Augmented Computational Frameworks	Cross-Scale Integrated Models/Hybrid Models
**Scientific Rationale**	Reconstitution of human immune system in immunodeficient mice to study tumor–immune dynamics.	Recapitulation of 3D tumor microenvironment with perfusion, multicellularity, and biomechanical forces.	Simulation and analysis of CRC using large-scale multi-omics, clinical, and imaging datasets.	Hybrid platforms combining models at various scales (subcellular to systemic) to study CRC progression, crosstalk, metastasis, and treatment response
**Model Design**	NSG or IL2rγ^−^/^−^ mice engrafted with CD34^+^ HSCs or PBMCs and implanted with CRC PDXs.	Microfabricated chambers lined with vasculature, seeded with CRC organoids/spheroids under flow conditions.	Ensemble of ML/DL models trained on genomic, proteomic, imaging, and clinical datasets for predictive tasks.	Integrates 3D organoid cultures, microfluidic (organ-on-chip) systems, and AI-based simulations (multi-omics, ML optimization)
**Experimental Utility**	Enables immunotherapy evaluation, adoptive T cell transfer, and immune profiling in vivo.	Captures real-time cell behavior, drug penetration, immune–tumor interaction, and metastasis simulation.	Predicts drug response, treatment outcomes, metastasis risk, and reveals resistance mechanisms.	Enables real-time tracking, modulation of tumor progression, immune-microbiome-tumor crosstalk, and treatment responses. Captures cell behavior, drug penetration, immune-tumor interaction, and metastasis simulation.
**Therapeutic Applications**	Assessment of anti–PD-1, CAR-T, bispecific antibodies in a human immune context.	Personalized ex vivo drug screens, immune cell infiltration modeling, metastatic colonization studies.	Digital twins for therapy selection, biomarker discovery, radiogenomic mapping, immune profiling.	Personalized ex vivo drug screens and therapy optimization; digital twins for treatment prediction; biomarker discovery; guidance for patient treatment schedules
**Immunologic Relevance**	High; allows analysis of human T/NK cell infiltration, checkpoint dynamics, and immune editing.	Moderate; immune cells (e.g., NK, T cells) can be perfused, but lacks full immune system integration.	Indirect; infers immune responses via modeling TME composition and immune evasion signatures.	High; allows incorporation of immune cells (T cells, dendritic cells) and microbiome-immune-tumor interactions, revealing immune evasion and drug resistance.
**Molecular Insights**	Tumor adaptation to immune attack (e.g., PD-L1 induction), pathway modulation under pressure.	Mechano-transduction, EMT induction under shear stress, metabolic heterogeneity under flow.	Signaling rewiring post-mutation, pathway convergence, synthetic lethality prediction.	Reveals mechano-transduction, stemness changes, EMT, metabolic heterogeneity, and signaling rewiring post-mutation. Identifies pathway convergence and synthetic lethality predictions.
**Translational Value**	Closely mimics patient response in immune-targeted therapies; suitable for preclinical validation.	High predictive accuracy for patient drug responses and potential for therapy optimization.	Highly scalable; facilitates hypothesis generation, patient stratification, and trial design.	High predictive accuracy for patient drug responses and therapy optimization; patient-representative; closely resembles patient tumors at transcriptomic level; facilitates patient stratification and trial design
**Limitations**	High cost, ethical issues, incomplete immune system, GvHD risk, short experimental window.	Fabrication complexity, lack of full systemic integration, low throughput, validation hurdles.	Requires massive, curated data, risk of overfitting, black-box nature, limited interpretability.	Technical complexity and low throughput. Lack of standardized protocols and reproducibility challenges. Incomplete TME. Complex data integration and limited data for AI training leading to overfitting or black-box issues
**Strategies to Overcome Limitations**	Use of iPSC-derived or autologous immune cells; HLA knock-ins; gene-editing to improve engraftment.	3D printing for reproducibility; immune-on-chip integration; automated fluidics; AI analytics.	Transfer learning, explainable AI, federated learning; integration with organoids, chips, and in vivo models.	High-density arrays and modular designs for throughput. Enhanced reproducibility. Standardized protocols and shared datasets for AI validation. Integration of immune/stromal elements and microbiome components. Augmented AI for interpretability.
**Integration with Other Models**	Organoid co-cultures for immune screening; AI for prediction; chip platforms for validation.	Used with PDOs, humanized mouse immune cells; feedback with in vivo and in silico models.	Connects data from organoids, PDXs, humanized mice, and patients; guides validation and next steps.	Integrates PDOs, microfluidics, and AI. Connects data from organoids, PDXs, humanized mice, and patients for validation and next steps. Future integration includes multi-organ networks.

**Table 4:** Molecular and phenotypic characterization of commonly used colorectal cancer cell lines.

Cell Line	Origin (Patient)	Tissue Origin	Stage	Karyotype	MSI Status	CMS	Key Driver Mutations	Functional Traits	Suitability	Limitations
**HT29**	44 y F, Caucasian	Primary Colon Adenoca.	Carcinoma	Hypertriploid (~71)	MSS (Likely)	CMS2/4	APC, BRAF V600E, TP53, PIK3CA, SMAD4	Enterocyte-like, GI barrier, MAPK/PI3K study	BRAF therapy, EGFR resistance, GI barrier	Aneuploidy, drift, TP53 mutant
**HCT116**	Adult M, Caucasian	Primary Colon Carcinoma	Carcinoma	Near-diploid (~45)	MSI-H	CMS1	KRAS G13D, PIK3CA H1047R, CTNNB1, TGFBR2, ACVR2A	MSI-H model, p53 WT/KO pair, Wnt activation	KRAS, PI3K, MSI-H, p53 function	EGFR resistance (KRAS mut), lacks stroma
**SW480**	50 y M, Caucasian	Primary Colon Adenoca.	Carcinoma (Dukes B/C)	Hypotriploid (~58)	MSS (Likely)	CMS2/3	KRAS G12V, APC, TP53	KRAS/APC hom mut, transfection model	KRAS-targeting, oxaliplatin resistance	Needs L-15 medium, CO_2_-free
**LoVo**	56 y M, Caucasian	Metastatic Colon Adenoca. (LN)	Metastatic (Stage IV equiv.)	Hyperdiploid (~48/49)	MSI-H	CMS1	KRAS G13D, APC, TGFBR2, ACVR2A, B2M, TP53 WT	Metastatic, CEA+, MHC-I loss (B2M)	Immune evasion, MSI-H therapy, metastatic model	B2M confers immune escape, complex CIN
**DLD-1**	Adult M, Caucasian	Primary Colon Adenoca.	Carcinoma	Pseudodiploid (~46)	MSI-H	CMS1	KRAS G13D, PIK3CA, TP53, APC, TGFBR2, ACVR2A, B2M	MSI-H, MHC-I loss, fast growth, shared origin	PI3K, MSI-H drug resistance, immune escape	Zygosity unclear, pseudodiploid, cross-contamination risks
**Caco-2**	72 y M, Caucasian	Primary Colon Adenoca.	Carcinoma	Hypertetraploid (~96)	MSS	CMS2/3	APC, TP53, SMAD4, CTNNB1	Drug permeability and differentiation model	Drug absorption, EGFR sensitivity model	Very slow growth, heterogeneity among stocks
**LS174T**	58 y F, Caucasian	Primary Colon Adenoca.	Carcinoma (Dukes B/Stage II)	Near-diploid (45,X)	MSI-H	CMS1	KRAS G12D, CTNNB1, PIK3CA, TP53 WT	Mucin producer, mucus barrier model	Mucus-targeted therapy, MSI-H	Less common CTNNB1 mutation, historical TP53 ambiguity

**Table 5:** Genetic alterations and targeting strategies in common colorectal cancer cell lines.

Cell Line	Mutation Profile	Gain of Function Mutations	Loss of Function Mutations	siRNA Targets	CRISPR Targets	Biological Context and Implications
**SW620**	KRAS mutant, TP53 mutant	KRAS	TP53	ISCA2, RNF135, NDUFA4L2	PPWD1, TCF7L2, RRAD	SW620 cells, due to KRAS and TP53 mutations, model metastatic CRC. CRISPR hits like TCF7L2 (Wnt signaling) and siRNA targets like NDUFA4L2 (hypoxia-linked) highlight oxidative stress and Wnt dependence in late-stage disease.
**Caco-2**	APC mutant, P53 wild-type	-	-	CYFIP1, BCAS2, CALD1	-	Caco-2, an epithelial model, is P53 wild-type. siRNA targeting CYFIP1 (cytoskeletal regulation) and BCAS2 (mRNA splicing) reflects epithelial barrier and differentiation control, critical in transport and absorption studies.
**RKO**	BRAF mutant, MLH1 hypermethylation	PIK3CA, BRAF	-	OGDH, ALDH18A1, POLR1G	WRN, ATP6V0E1, CAD	RKO is MSI-H and BRAF mutant, making it ideal for studying epigenetically driven CRC. CRISPR hits like WRN and ATP6V0E1, and siRNA targets like ALDH18A1, link to metabolic stress and replication stress in MSI-H tumors.
**SW480**	KRAS mutant, APC mutant	KRAS	TP53	MED30, EML4, RASGEF1C	-	SW480 harbors KRAS and APC mutations and is used in canonical CIN modeling. siRNA hits like MED30 (transcription regulation) and CRISPR knockouts affecting none reflect stability under transcriptional disruption.
**HT8**	Unknown	-	-	-	-	HT8’s unknown profile limits current application; its use remains restricted until further profiling is available.
**HT29**	BRAF mutant, P53 mutant	PIK3CA, BRAF	TP53	GINS2, AHCTF1, RAB6A	PPP2CA, SCD, PCYT1A	HT29, with BRAF and PIK3CA gain and TP53 loss, reflects CIN and MAPK pathway activation. CRISPR targets like SCD (lipid metabolism) and PPP2CA (phosphatase control) indicate signaling and metabolic vulnerabilities.
**HCT116**	KRAS mutant, MLH1 mutant	KRAS, CTNNB1, PIK3CA	-	DDX27, WRN, SAFB	WRN, TIMM17A, TEX10	HCT116, an MSI-H model with wild-type TP53, allows for functional dissection of MMR loss and KRAS-driven tumorigenesis. CRISPR and siRNA targets converge on WRN and ribosomal biogenesis genes, linking DNA repair to proliferation.
**LoVo**	KRAS mutant, BRAF wild-type	KRAS, PIK3CA	-	RAE1, TACC3, WRN	ACTR8, RPL22L1, PIK3CB	LoVo, metastatic and MSI-H, with KRAS and PIK3CA mutations, shows CRISPR/siRNA hits tied to ribosomal biosynthesis and chromosomal segregation (ACTR8, TACC3), ideal for stress response and metastasis research.
**LS174T**	KRAS mutant, APC mutant	-	-	-	-	LS174T remains under-characterized for gene editing applications but is useful for mucin biology and CMS1-like CRC phenotypes.

**Table 6:** Comparative analysis of murine models for colorectal cancer research.

Model Type	Tumor Induction Method	Immune Competence	Time to Tumor Formation	Molecular Fidelity to Human CRC	Use Cases	Limitations	References
**Chemically Induced e.g., AOM (±DSS) model**	Carcinogen (AOM) often with DSS to induce colitis	Intact immune system	~8–20 weeks	Moderate; random mutations (β-catenin, Kras), mimics colitis-associated CRC	- Studying environmental risk factors and inflammation-driven tumorigenesis - Chemoprevention trials - Modeling colitis-associated cancer	- Requires prolonged treatment and can be variable - Tumors are often non-metastatic and oligoclonal - Mutation spectrum differs from typical human CRC - Not ideal for testing targeted therapies	[125,126]
**Genetically Engineered (GEMM) e.g., APC^Min/+^, Villin-Cre; Apc^fl/fl^, etc.**	Germline or conditional mutations in tumor suppressors/oncogenes.	Intact immune system	Varies: 2–6 months for polyps (APCMin); 8–12+ months for invasive carcinoma	High for initiating events; can capture stepwise progression. Some mirror human histology	- Investigating specific gene roles in CRC initiation/progression (Wnt, TP53, KRAS, MMR genes, etc.) - Studying tumor evolution and microenvironment in native setting - Preclinical testing in defined genetic contexts	- Time- and resource-intensive - Many models yield mainly benign tumors or few invasive cancers without additional hits - Tumor location may differ from humans - Limited tumor heterogeneity; all tumors driven by same engineered mutation(s)	[127,128,129]
**Xenograft—Cell Line (CDX)**	Injection of human CRC cell line into immunodeficient mouse	Lacks adaptive immunity	Fast: 2–6 weeks	Moderate; uses human cancer cells but clonal and culture-adapted	- Rapid in vivo drug efficacy screening - Mechanistic studies (easy to modify tumor cells genetically before injection) - Radiation therapy and imaging research	- No immune context - Heterotopic growth (subcutaneous) lacks normal colon environment - Cell lines often from late-stage cancers - Murine stroma and vasculature supporting human cells can alter drug responses	[130,131]
**Xenograft—Patient-Derived (PDX)**	Implantation of human CRC tissue fragment into immunodeficient mouse	Lacks adaptive immunity	Moderate: 2–4 months initial engraftment; 1–3 months for subsequent passages	High; preserves original tumor architecture, heterogeneity, and molecular profile	- Co-clinical trials and personalized medicine models - Studying drug resistance mechanisms - Biomarker discovery for therapy response	- Requires specialized infrastructure and biobanking; not high-throughput - Engraftment bias - Still lacks human immunity; mouse stromal replacement occurs over passage	[132,133]
**Syngeneic (Mouse Allograft) e.g., CT26 in BALB/c, MC38 in C57BL/6**	Transplant of murine CRC cells/tumor chunks into genetically identical mouse	Fully immunocompetent.	Very fast: 1–3 weeks (subQ); 3–6 weeks (orthotopic/metastatic)	Low-Moderate; mouse origin, often high mutation burden	- Immuno-oncology studies: evaluate checkpoint inhibitors, cancer vaccines, CAR-T, etc., in vivo with native immunity. - Testing of combination therapies (chemo/radiation + immunotherapy) - Tumor microenvironment research.	- Not human-derived, so findings need validation in human cells. - Limited number of available models - Some models are extremely aggressive or carry artificial mutations, - Subcutaneous syngeneic tumors rarely metastasize spontaneously	[134,135]
**Orthotopic Implantation (applied to CDX, PDX, or syngeneic models)**	Surgical or injection-based placement into mouse colon/caecal wall or rectum	Varies (immunocompetent for syngeneic, immunodeficient for human).	Moderate: 2–4 weeks establishment; 6–12 weeks for invasive growth/metastases	High tissue fidelity; molecular fidelity depends on source (high for PDX). Often recapitulates organ-specific metastatic cascade	- Metastasis research: spontaneous spread to liver/lymph nodes enables study of metastatic colonization and dormancy. - Evaluation of locoregional therapies in a realistic setting. - Studying tumor–microbiome and tumor–stroma interactions unique to the colonic niche	- Surgical skill required, higher variability and animal morbidity. - Monitoring is difficult: tumor is internal - requires imaging or periodic sacrifices to assess tumor progression - Lower throughput - If using human tissue, lacks immune component; if using mouse tissue, lacks human tumor genetics	[136,137]

**Table 7:** Summary of representative studies in advanced colorectal cancer models.

Research Focus	Model System	Key Findings	Reference
***CRISPR-Edited Organoids***			
Identifying Novel Genetic Dependencies	Genome-wide CRISPR screen in CRC organoids	Identified 13 novel therapeutic targets unique to the 3D organoid model, not apparent in 2D screens.	[350]
Overcoming Chemoresistance	Oral CRISPR-nanoparticle therapy in CRC organoids and mouse models	CRISPR-mediated knockout of *TRAP1* re-sensitized CRC models to chemotherapy and boosted anti-tumor immunity.	[351]
Clinical Translation of Gene Editing	First-in-human trial of CRISPR-edited TILs	Ex vivo CRISPR knockout of the intracellular checkpoint *CISH* in TILs was safe and led to durable complete response in a CRC patient.	[352]
***Humanized Mice***			
Validating Immunotherapy for “Cold” Tumors	HLA-A2.1-matched humanized mouse with *KRAS*-mutant CRC	Combination immunotherapy induced potent, tumor-specific, and HLA-restricted T-cell responses and tumor control only in the fully HLA-matched model.	[334]
Testing Novel Epigenetic mRNA Therapy	Humanized mouse model with CRC xenografts	An mRNA therapy delivering a peptide inhibitor (mSTELLA) of the oncogene *UHRF1* activated tumor suppressors and impaired tumor growth in vivo.	[353]

**Table 8:** Key limitations and corresponding mitigation strategies.

Model Type	Limitation	Mitigation Strategy/Future Direction	Key Reference
***CRISPR-Edited Organoids***	Absence of a complete Tumor Microenvironment (TME) (no stroma, vasculature, or immune cells).	Development of advanced co-culture systems with fibroblasts (CAFs) and immune cells; integration into Organ-on-a-Chip devices to introduce flow and multi-tissue interfaces.	[354]
	Culture variability and lack of standardization (reliance on Matrigel).	Development of chemically defined, synthetic hydrogels to improve reproducibility and reduce batch-to-batch variability.	[355]
	CRISPR off-target effects and editing inefficiencies.	Use of high-fidelity Cas9 nucleases, optimized gRNA design (bioinformatics, chemical modifications), transient RNP delivery, and unbiased off-target detection methods (e.g., GUIDE-seq).	[355]
***Humanized Mice***	Graft-versus-Host Disease (GvHD), especially in PBMC models.	Use of immunodeficient mouse strains lacking host MHC class I and II molecules (e.g., NSG-MHC DKO) to abrogate T-cell reactivity against mouse tissues.	[356]
	Incomplete or skewed immune reconstitution (poor myeloid/NK development, suboptimal B-cell function).	Engineering of “next-generation” mice expressing human cytokine (e.g., NSG-SGM3, NSG-IL15) and HLA transgenes to support broader and more functional immune development.	[327]
	Confounding immunological effects (e.g., Graft-versus-Tumor).	Use of autologous models (patient tumor + patient immune cells) to minimize alloreactivity; careful monitoring and inclusion of appropriate control groups.	[357]
	High cost, complexity, and ethical constraints.	Streamlining protocols, improving engraftment efficiency, and developing alternatives to ethically sensitive tissue sources (e.g., fetal tissue for BLT models).	[14]

## Data Availability

The datasets generated and/or analyzed during the current study are not publicly available but are accessible from the corresponding author upon reasonable request. Interested researchers may contact the corresponding author for data access inquiries, subject to compliance with any applicable privacy or confidentiality obligations.

## Abbreviations

2D	Two-Dimensional (cell culture)
3D	Three-Dimensional (cell culture)
5-FU	5-Fluorouracil
ACS	American Cancer Society
AI	Artificial Intelligence
ALI	Air-Liquid Interface
AOM	Azoxymethane
APC	Adenomatous Polyposis Coli (gene)
ASR	Age-Standardized Incidence Rate
ATM	Ataxia Telangiectasia Mutated (gene)
ATR	Ataxia Telangiectasia and Rad3-Related (gene)
AUROC	Area Under the Receiver Operating Characteristic Curve
BRAF	B-Raf Proto-Oncogene (gene)
CAF	Cancer-Associated Fibroblast
CAR-T	Chimeric Antigen Receptor T-cell
CD34+	Hematopoietic Stem Cell Marker
CDX	Cell Line-Derived Xenograft
CEA	Carcinoembryonic Antigen
CIMP-high	High CpG Island Methylator Phenotype
CIMP	CpG Island Methylator Phenotype
CIN	Chromosomal Instability
CMS	Consensus Molecular Subtype
COX-2	Cyclooxygenase-2
CpG	Cytosine-Guanine Dinucleotide (DNA methylation site)
CRC	Colorectal Cancer
Cre-lox	Site-Specific Recombinase System
CRISPR	Clustered Regularly Interspaced Short Palindromic Repeats
CT26	Murine Colon Carcinoma Cell Line
ctDNA	Circulating Tumor DNA
CTLA-4	Cytotoxic T-Lymphocyte-Associated Protein 4
DL	Deep Learning
dMMR	Deficient Mismatch Repair
DSS	Dextran Sulfate Sodium
ECM	Extracellular Matrix
EGFR	Epidermal Growth Factor Receptor
EMT	Epithelial–Mesenchymal Transition
EO-CRC	Early-Onset Colorectal Cancer
ERBB2	Erythroblastic Oncogene B2
F0/F1/F2	Generations in PDX Models
FAP	Familial Adenomatous Polyposis
FAP	Fibroblast Activation Protein
FGFR2	Fibroblast Growth Factor Receptor 2
FOLFIRI	Folinic Acid, Fluorouracil, Irinotecan
FOLFOX	Folinic Acid, Fluorouracil, Oxaliplatin
GEMM	Genetically Engineered Mouse Model
GREM1	Gremlin-1 (BMP antagonist)
GWAS	Genome-Wide Association Study
HCT116	Human CRC Cell Line (MSI-high, KRAS mutant)
HER2	Human Epidermal Growth Factor Receptor 2
HIF	Hypoxia-Inducible Factor
HLA	Human Leukocyte Antigen
HNPCC	Hereditary Nonpolyposis Colorectal Cancer (Lynch Syndrome)
HPFS	Health Professionals Follow-Up Study
HT29	Human CRC Cell Line (MSS, BRAF mutant)
IACUC	Institutional Animal Care and Use Committee
ICI	Immune Checkpoint Inhibitor
ICIs	Immune Checkpoint Inhibitors
IGF-1	Insulin-Like Growth Factor 1
IL-6/IL-8	Interleukin-6/Interleukin-8
IVIS	In Vivo Imaging System
KRAS	Kirsten Rat Sarcoma Viral Oncogene Homolog (gene)
LGR5	Leucine-Rich Repeat-Containing G-Protein Coupled Receptor 5 (stem cell marker)
LoVo	Human CRC Cell Line (MSI-high, derived from metastasis)
LS	Lynch Syndrome
MAPK	Mitogen-Activated Protein Kinase
MC38	Murine Colon Adenocarcinoma Cell Line
MCP-1	Monocyte Chemoattractant Protein-1
MEK	Mitogen-Activated Protein Kinase Kinase
ML	Machine Learning
MLH1	MutL Homolog 1 (MMR gene)
MLH1/MSH2/MSH6/PMS2	Mismatch Repair Genes
MMR	Mismatch Repair
MSI-H	Microsatellite Instability-High
MSI	Microsatellite Instability
MSS	Microsatellite Stable
NF-κB	Nuclear Factor Kappa-Light-Chain-Enhancer of Activated B Cells
NHP	Non-Human Primate
NHS	Nurses’ Health Study
NK	Natural Killer (cell)
NOD-SCID	Non-Obese Diabetic-Severe Combined Immunodeficiency (mice)
NSAIDs	Non-Steroidal Anti-Inflammatory Drugs
NSG	NOD-SCID-IL2Rγnull (immunodeficient mice)
OLFM4	Olfactomedin-4 (stem cell marker)
OoC	Organ-on-a-Chip
PAR-2	Protease-Activated Receptor-2
PBMC	Peripheral Blood Mononuclear Cell
PD-1/PD-L1	Programmed Death-1/Programmed Death-Ligand 1
PDMS	Polydimethylsiloxane (microfluidic material)
PDO	Patient-Derived Organoid
PDOX	Patient-Derived Organoid Xenograft
PDX	Patient-Derived Xenograft
PI3K	Phosphoinositide 3-Kinase
PIK3CA	Phosphatidylinositol-4,5-Bisphosphate 3-Kinase Catalytic Subunit Alpha (gene)
R-spondin	Wnt Pathway Agonist
RAS	Rat Sarcoma (gene family)
RAS	Rat Sarcoma Virus
RRR (3Rs)	Replacement, Reduction and Refinement
SCFA	Short-Chain Fatty Acid
SHAP	SHapley Additive exPlanations (AI interpretability tool)
SMAD4	Mothers Against Decapentaplegic Homolog 4 (TGF-β pathway)
SNP	Single Nucleotide Polymorphism
STAT3	Signal Transducer and Activator of Transcription 3
SW480	Human CRC Cell Line (MSS, KRAS mutant)
TCGA	The Cancer Genome Atlas
TCR-T	T-Cell Receptor-Engineered T-cell
TGF-β	Transforming Growth Factor Beta
TME	Tumor Microenvironment
TNBS	Trinitrobenzene Sulfonic Acid (colitis inducer)
TNF-α	Tumor Necrosis Factor-Alpha
TP53	Tumor Protein p53 (gene)
VEGF	Vascular Endothelial Growth Factor
WHO	World Health Organization
WNT	Wingless-Type MMTV Integration Site Family (signaling pathway)
WRN	Werner Syndrome Helicase (synthetic lethal target in MSI CRC)
β-catenin	Key Wnt Pathway Effector
